# Repositioning HDAC Inhibitors for Glioma Treatment: Synthesis and Biological Evaluation

**DOI:** 10.1021/acsomega.5c11083

**Published:** 2026-02-03

**Authors:** Luciana Costa Furtado, Karoline de Barros Waitman, Nuno A. T. F. Silva, Leticia Marcelino Gouvea, Thales Kronenberger, Mônica Franco Zannini Junqueira Toledo, Elthon Gois Ferreira, João Agostinho Machado-Neto, Frank A. E. Kruyt, Roberto Parise Filho, Letícia V. Costa-Lotufo

**Affiliations:** † Department of Pharmacology, Institute of Biomedical Sciences, 28133 University of São Paulo, Avenida Professor Lineu Prestes 1524, 05508-000 São Paulo, Brazil; ‡ Department of Medical Oncology, University Medical Center Groningen, University of Groningen, DA11, Postbus 30.001, 9700 RB Groningen, The Netherlands; § Department of Pharmacy, Faculty of Pharmaceutical Sciences, University of São Paulo, Avenida Professor Lineu Prestes, 580, 05508-000 São Paulo, Brazil; ∥ Interfaculty Institute of Microbiology and Infection Medicine (IMIT), 27203 University of Tübingen, 72076 Tübingen, Germany; ⊥ Partner-site Tübingen, German Center for Infection Research (DZIF), 72076 Tübingen, Germany; # School of Pharmacy, Faculty of Health Sciences, University of Eastern Finland, P.O. Box 1627, FI-70211 Kuopio, Finland

## Abstract

Gliomas are a type of brain tumor associated with poor patient prognosis, with current treatment, surgical resection when feasible, followed by radiotherapy and chemotherapy (Temozolomide), yielding a median survival of approximately 15 months. In light of the urgent need for more effective therapies, histone deacetylases (HDACs) have emerged as promising targets, given their differential expression across tumor types and disease grades. Although HDAC inhibitors are well established in the treatment of hematological malignancies, their potential is now being explored in solid tumors, including glioblastoma (GBM). In this study, hydroxamate-based (**3a**) and benzamide-based (**6a**) HDAC inhibitors were synthesized and evaluated in glioma cell lines and glioblastoma stem cells (GSC). Treatment with these inhibitors resulted in cell cycle alterations, increased SubG1 populations, and enhanced apoptosis, particularly with compound **3a**. Notably, **6a** demonstrated greater potency in GSCs. The observed cytotoxic effects were linked to selective inhibition of HDAC6 by **3a** and HDAC1/3 by **6a**, as confirmed through enzymatic assays and further supported by molecular docking and molecular dynamics (MD) simulations. *In silico* analyses suggest that both compounds possess favorable pharmacokinetic profiles, underscoring their potential as promising candidates for glioma therapy and paving the way for future drug development in this field.

## Graphical abstract

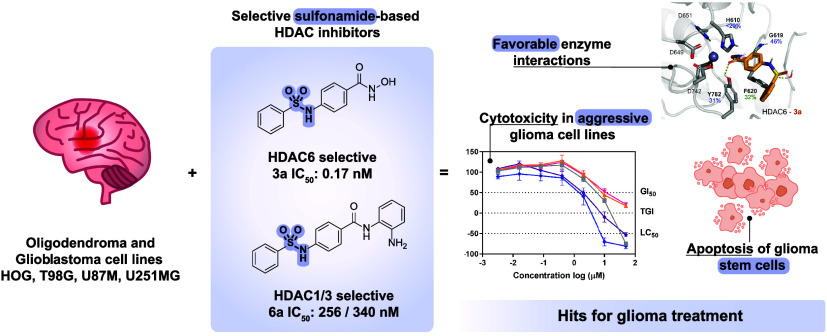

## 1 Introduction

Gliomas are the most common type of cancer affecting the central nervous system (CNS),^1^ accounting for 80% of malignant brain tumors and most of the associated demises.^2,3^ The diagnosis and treatment options for these tumors are scarce due to their localization and diffuse nature, with most patients relying on safe surgical resection, radiotherapy, and Temozolomide chemotherapy (Figure 1A).^3^ A portion of gliomas are associated with aberrant isocitrate dehydrogenase (IDH) activity and epigenetic alterations, with histone 3 (H3) alterations being considered essential diagnostic markers of the disease as well as methylation alterations.^4,5^ However, the most aggressive and resistant grade IV gliomas (glioblastomas) usually do not bear IDH mutations,^2^ rendering IDH inhibitors, such as vorasidenib (Figure 1A), noneffective, urging the need for novel targeted therapies.

**Figure 1:**
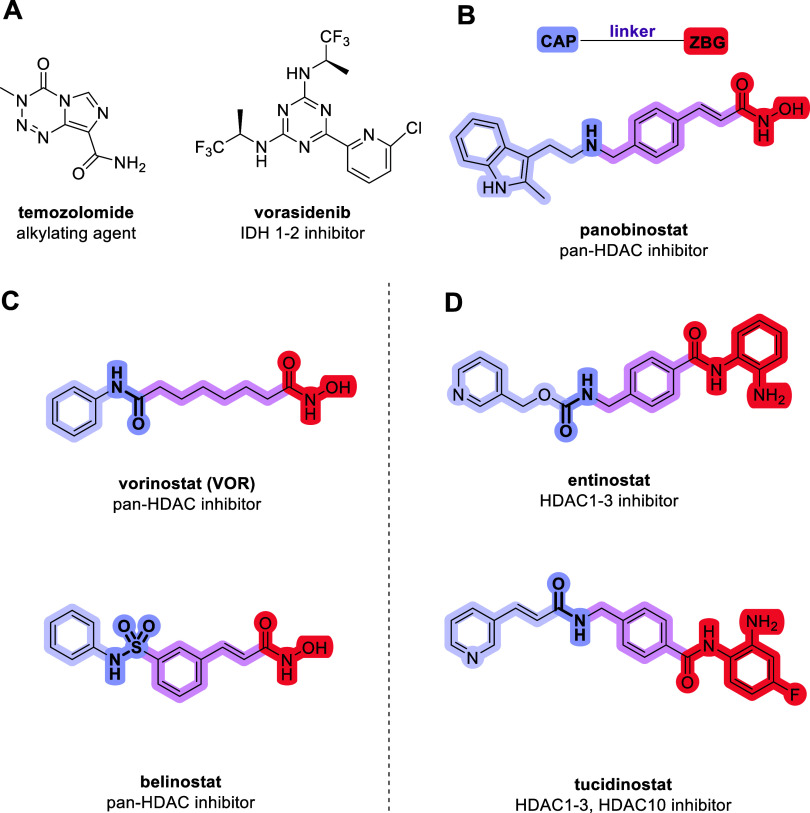
Current and novel experimental strategies to treat glioma. (A) Current drugs employed in glioma treatment. Novel strategies to treat glioma include (B) the HDAC inhibitor panobinostat, with its pharmacophore drawn above. (C, D) Examples of pan-HDAC inhibitors. Highlighted in purple is the cap group with the connecting unit depicted in bold and in higher saturation. In pink, the linker portion, and in red, the ZBG.

Histone deacetylases (HDACs) are epigenetic mediators responsible for the removal of acetyl functional groups from the lysine residues of histone proteins, thereby silencing the affected genes.^6^ HDAC enzymes are classified in four big classes based on their primary homology to yeast HDACs – I, II, III, and IV – and can be Zn^2+^ or NAD^+^-dependent.^7^ Among the 11 known zinc-dependent HDAC isoforms, Class I HDACs (HDAC1, HDAC2, HDAC3, and HDAC8) are frequently upregulated in glioma cell lines and have been associated with tumor progression, poor prognosis, and Temozolomide resistance in patients.^8^ Knockdown of HDAC1 expression in tumor xenografts in mice promoted apoptosis and reduced infiltration of glioma cell lines,^9^ and although HDACs class IIb (HDAC6 and HDA10) are usually less associated with the disease,^9,10^ a recent study pointed out that HDAC6 knockdown can also reduce invasiveness *in vivo*, driving the growth of IDH mutant gliomas,^11^ and therefore, HDACs can be relevant targets for treat this cancer.

Drug screening efforts in gliomasphere cell lines (IDH1 mutant, and IDH1 wildtype) identified HDAC inhibitors, in particular, panobinostat (Figure 1B), as a promising approach for glioma treatment, either as a monotherapy in IDH mutant cell lines,^11^ or in combined therapies,^12^ by restoring H3 methylation patterns and blocking the transcription of oncogenes.^13^ Given the potential of HDAC inhibition in glioma treatment, several HDAC inhibitors were enrolled in clinical trials.^5,14^ Vorinostat (VOR) (Figure 1C) has shown good tolerability in phase II trials of glioblastomas, even prompting a combination therapy with other antitumoral agents; however, no increases in efficacy have been noted so far.^5^

Although HDAC inhibitors have yet to be approved as a monotherapy for solid tumors, many have been successful in the treatment of hematological malignancies,^15^ and show promise in combined therapy efforts (Figure 1C,D).^16^ The FDA-approved drug belinostat, for example, is currently enrolled in five different clinical trials for the treatment of solid neoplasias (Clinical Trial IDs: NCT05154994, NCT05170334, NCT04315233, NCT02137759, and NCT04340843),^17^ including newly diagnosed glioblastomas, in combination with standard radiation therapy and Temozolomide (NCT02137759). However, most trials so far explored only classic hydroxamate pan-inhibitors, which could be problematic due to their high toxicity and off-target cardiotoxic side effects,^18^ urging the need to examine novel isoform-selective counterparts as potential therapeutic approaches for high-grade gliomas. Here, we designed a series of novel HDAC inhibitors bearing different zinc-binding groups (ZBGs) and a phenyl-sulfonamide capping group, resembling belinostat, to toggle selectivity for different HDAC isoforms, aiming to improve the treatment options for a disease whose survival has not increased in the last decades.

## 2 Results and Discussion

### 2.1 Design and Synthesis of Novel HDAC Inhibitors

The design of novel HDACi compounds started with phenylsulfonamides as a capping group, mimicking belinostat (Figure 2). Sulfonamides were not extensively explored as HDAC inhibitors and provide increased water solubility compared to amides,^19^ and are present in a wide range of approved drugs.^20^ To that, a phenylic linker was attached either in *para* (Series A) or *meta* (Series B) position, followed by different chelating groups to act as ZBGs described in the literature to provide chemical diversity.^15,21–24^

**Figure 2:**
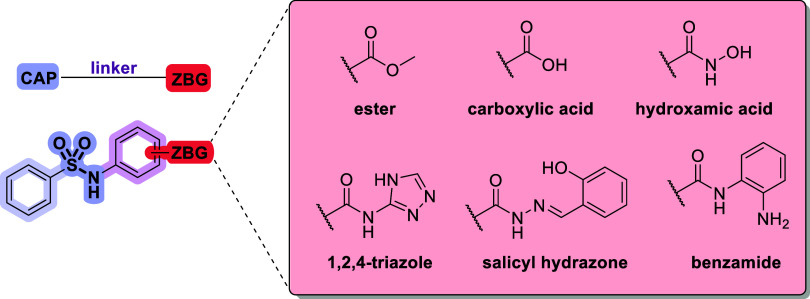
General pharmacophore and chemical structures of novel HDAC inhibitors. Depicted are the cap in light purple, the linker in pink, and the zinc-binding group (ZBG) in red. The box showcases the different ZBGs chosen for HDAC inhibition.

Figure 3 depicts the synthetic route employed to prepare the designed compounds. Methyl aminobenzoates were reacted with phenylsulfonyl chloride, yielding either *meta* or *para N*-sulfonated esters **1a**–**b**. They were subsequently hydrolyzed under basic conditions to make carboxylic acids **2a**–**b**. The acids **2a**–**b** were either condensed with 3-amino-1,2,4-triazole to generate triazoles **5a**–**b** or coupled with 1,2-diaminobenzene to yield the corresponding benzamides **6a**–**b**. Alternatively, **1a**–**b** were directly reacted with hydroxylamine to produce hydroxamic acids **3a**–**b** or subjected to hydrazinolysis, followed by condensation with salicylic aldehyde to give salicyl hydrazones **4a**–**b**.

**Figure 3:**
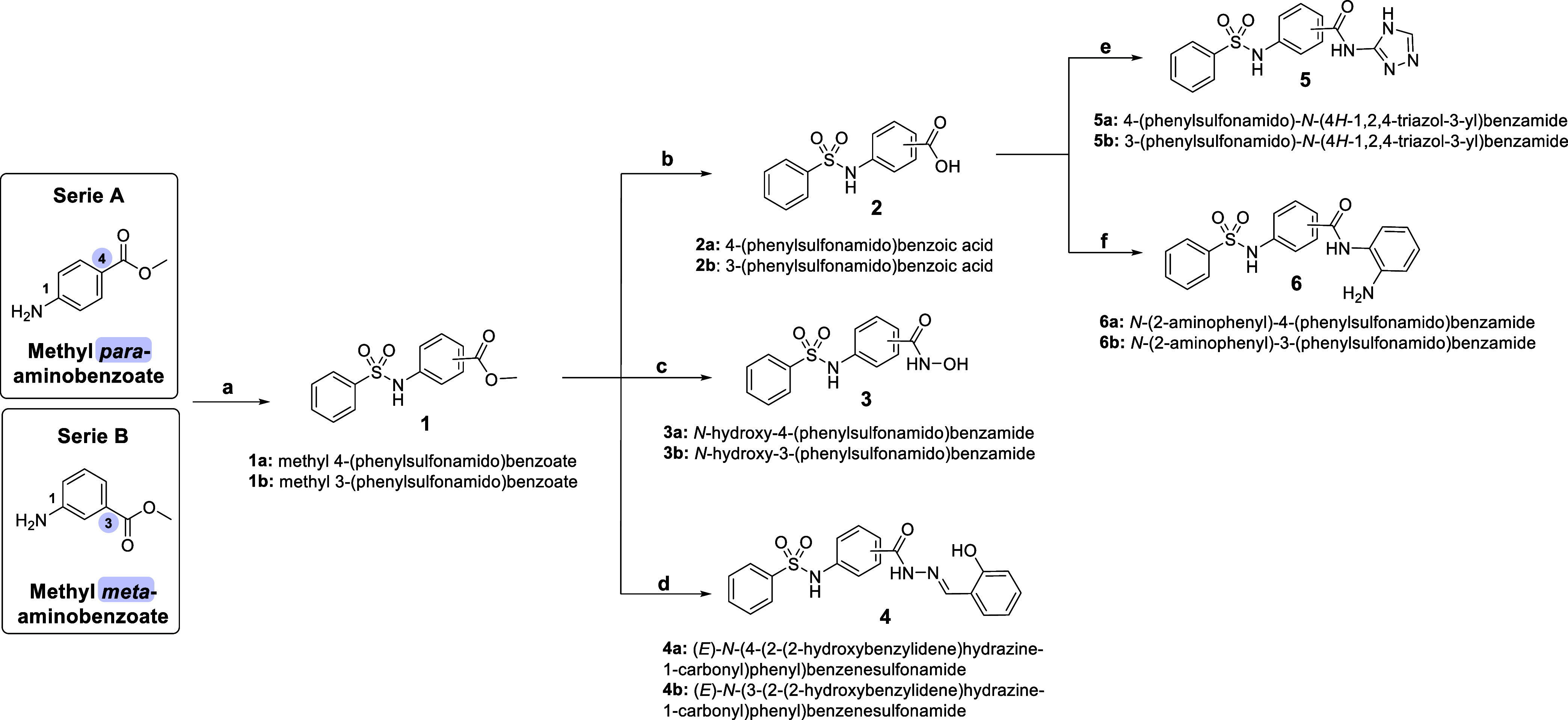
Synthesis of HDACi test compounds **1**–**6**. Reactants and conditions: (a) Methyl aminobenzoate (1.2 equiv), PhSO_2_Cl (1 equiv), Na_2_CO_3_ (sat.), THF, H_2_O, r.t.;^25^ (b) **1** (1 equiv), KOH (sat.), H_2_O, reflux;^26^ (c) **1** (1 equiv), NH_2_OH (aq.; 8 equiv), NaOH (50%), THF, MeOH, r.t.;^27^ (d) 1. **1** (1 equiv), NH_2_NH_2_ (80%), MeOH, reflux; 2. Salicylic aldehyde (1.2 equiv), AcOH (cat.), EtOH, r.t.;^28,29^ (e) **2** (1 equiv), 3-amino-1,2,4-triazole (1.1 equiv), EDC (1.2 equiv), DMAP (cat.), DCM, r.t.;^30,31^ and (f) **2** (1 equiv), 1,2-diaminobenzene (1.1 equiv), EDC (1.2 equiv), DMAP (cat.), DCM, r.t.^32^

### 2.2 **3a** and **6a** inhibited the proliferation of oligodendroglioma and glioblastoma cells

An initial screening of the synthesized compounds (at 50 μM) against the glioma cell lines HOG and T98G identified **3a**, **4a**, **4b**, and **6a** as hits, inhibiting at least 75% of the cell proliferation in both cell lines (Figure S1). Except for **4b**, all these compounds have their ZBG attached in the *para* position of the linker, which can favor a less constrained binding geometry in the rim of the HDACs.^21^ The choice of different ZBG also affected the compounds’ potency, with esters **1a**–**b**, carboxylic acids **2a**–**b**, and 1,2,4-triazoles **5a**–**b** being detrimental to activity. The salicyl acyl-hydrazones **4a**–**b** were potent inhibitors of HOG cells but presented moderate activity against the more aggressive glioblastoma cell line, T98G. Compounds **3a** and **6a**, bearing traditional ZBGs hydroxamic acid and benzamide, respectively, completely inhibited the cell growth in both glioma cell lines (Figure S1), and together with **4a**–**b**, they were chosen for further analysis.

Subsequently, the selected compounds (**3a**, **4a**–**b**, **6a**) were evaluated in four glioma cell lines, the two previous ones (HOG, T98G), and more proliferative and invasive glioblastoma cell models (U87MG, and U251MG, respectively)^33^ at three different treatment time points (24, 48, and 72 h) (Figure 4). Compounds **3a** and **6a** were more active than **4a**–**b** in all cell lines, with **6a** being slightly more active than **3a** for most cells except for T98G (Table 1). Their activities were time-dependent since most GI_50_ values were lower after 72 h of incubation, while after 24 h of incubation, only HOG cells were sensitive to the tested compounds, except for compound **6a**. The obtained GI_50_ values after 72 h of incubation ranged from 0.8 μM in T98G to 5.3 μM in U87MG for compound **3a**, and from 0.6 μM in U251MG to 1.6 μM in T98G for compound **6a**.

**Figure 4:**
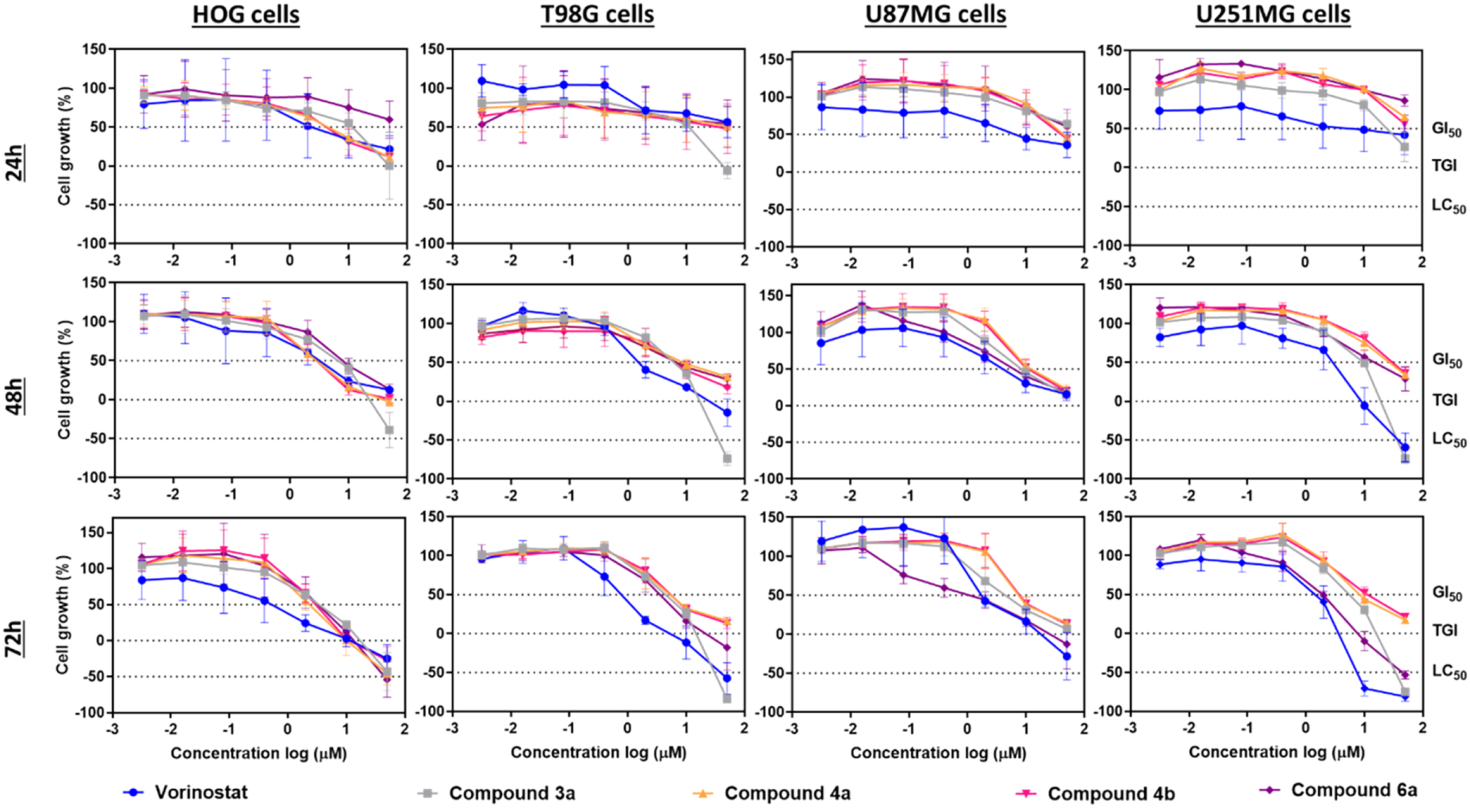
Cytotoxic effects of the newly synthesized compounds. Cell growth curves of HOG, T98G, U87MG, and U251MG were exposed to compounds **3a** (gray), **4a** (orange), **4b** (pink), **6a** (purple), and vorinostat (blue) at three different treatments (24–72 h) measured by the Sulforhodamine B (SRB) method. Mean growth inhibitory concentration (GI_50_), total growth inhibition (TGI), and mean lethal concentration (LC_50_) values were obtained to indicate the potency of the compounds and cytostatic and cytotoxic effects. Data corresponds to the mean ± standard error of the mean from 3 independent experiments.

**Table 1:** Cytostatic and cytotoxic effects concentrations of HDAC inhibitors on glioma cell lines^a^

	HOG			T98G			U87MG			U251MG		
	GI_50_	TGI	LC_50_	GI_50_	TGI	LC_50_	GI_50_	TGI	LC_50_	GI_50_	TGI	LC_50_
GI_50_, TGI, and LC_50_ values (μM) 24 h												
vorinostat	2.3	>50	>50	>50	>50	>50	11.4	>50	>50	10.2	>50	>50
compound **3a**	3.2	>50	>50	2.9	>50	>50	>50	>50	>50	>50	>50	>50
compound **4a**	2.6	>50	>50	>50	>50	>50	>50	>50	>50	>50	>50	>50
compound **4b**	2.4	>50	>50	>50	>50	>50	>50	>50	>50	>50	>50	>50
compound **6a**	>50	>50	>50	>50	>50	>50	>50	>50	>50	>50	>50	>50
GI_50_, TGI, and LC_50_ values (μM) 48 h												
vorinostat	2.5	>50	>50	1.4	48.6	>50	4.6	>50	>50	0.4	13.2	>50
compound **3a**	1.8	>50	>50	1.1	30.6	>50	20.3	>50	>50	1.5	44.8	>50
compound **4a**	2.3	>50	>50	22.9	>50	>50	>50	>50	>50	>50	>50	>50
compound **4b**	2.2	>50	>50	12.6	>50	>50	>50	>50	>50	>50	>50	>50
compound **6a**	10.2	>50	>50	7.7	>50	>50	8.7	>50	>50	31.6	>50	>50
GI_50_, TGI, and LC_50_ values (μM) 72 h												
vorinostat	0.2	14.2	>50	0.4	5.8	>50	2.0	37.2	>50	0.2	2.4	30.4
compound **3a**	1.1	34.0	>50	0.8	16.2	>50	5.3	>50	>50	1.3	28.9	>50
compound **4a**	1.0	20.2	>50	7.2	>50	>50	18.1	>50	>50	23.4	>50	>50
compound **4b**	1.7	42.8	>50	6.9	>50	>50	19.5	>50	>50	35.3	>50	>50
compound **6a**	1.2	21.9	>50	1.6	>50	>50	0.7	35.8	>50	0.6	10.8	>50

a Growth inhibition (GI_50_), total growth inhibition (TGI), and lethal concentration (LC_50_) values of compounds **3a**, **4a,b**, **6a**, and vorinostat in HOG, T98G, U87MG, and U251MG at 24, 48, and 72 h.

Although the onset of vorinostat activity was earlier (after 24 h in 3/4 cell lines, Figure 4) compared to the tested compounds **3a** and **6a**, with GI_50_ values ranging from 2.3 μM in HOG to 11.4 μM in U87MG (Table 1), the GI_50_ values of the tested compounds became comparable to vorinostat after 72 h of incubation (Figure 4). These results guided the selection of compounds **3a** and **6a** for further evaluation.

### 2.3 The antiproliferative effects of **3a** and **6a** are linked to HDACs inhibition

Subsequently, compounds **3a** and **6a** were evaluated *in vitro* for the inhibition of different HDAC isoforms in dose–response experiments. Both compounds were able to inhibit more than 50% HDACs activity, though with different selectivities (Table 2 and Figure S2). The hydroxamic acid derivative (**3a**) was a potent and selective HDAC6 inhibitor in the nanomolar range (IC_50_: 0.17 nM), while inhibiting class I HDACs (HDAC1, HDAC2, HDAC3, and HDAC8) with over 1000-fold less potency. For comparison, vorinostat inhibited HDAC6 with an IC_50_: 21.7 nM and showed only a 5-fold selectivity over HDAC1 (IC_50_: 113 nM).^34^ Furthermore, compound **3a** was, in fact, twice as potent in HDAC6 than the positive control trichostatin A, and presented a selectivity index of more than 17000-fold over HDAC10 (Table 2). Interestingly, compound **3a** presented a higher affinity for HDACs class I than HDAC10, which might be an atypical affinity profile or can be related to the substrate of choice for the assays, since HDAC6 and HDAC10 are both class IIb HDACs, and usually compounds which inhibit HDAC6 also present off-target activity in HDAC10.^35^ The benzamide derivative (**6a**), on the other hand, inhibited only class I HDACs with IC_50_ values in the micromolar range, being inactive on class IIb HDACs even at highest tested concentration (0.1 mM), which agrees with the literature.^36^ Besides the off-class selectivity observed, compound **6a** also presented in-class selectivity, inhibiting HDAC1 and HDAC3 with similar nanomolar potency (IC_50_: 256–340 nM), while presenting micromolar activity in HDAC2, and HDAC8 (Table 2). This inhibition profile aligns with the literature, where phenyl hydroxamates have selectivity for class IIb HDACs, while benzamides show class I selectivity.^13,14^

**Table 2:**
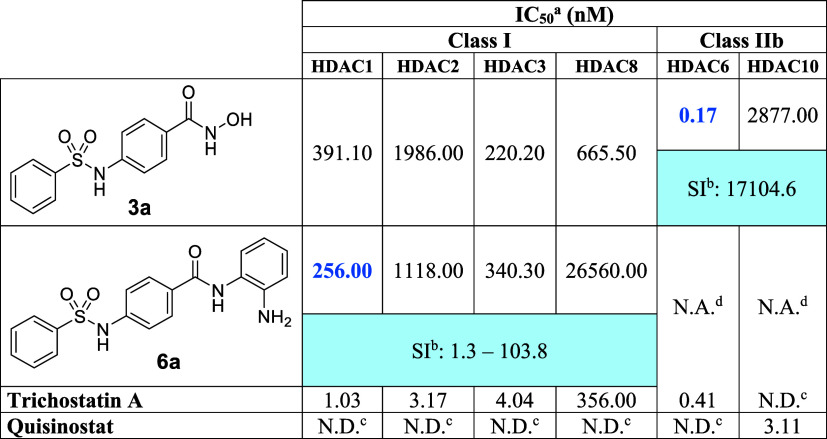
Inhibitory activities of 3a and 6a in selected HDAC isoforms

a IC_50_ values are the mean of two experiments obtained from curve-fitting of a 10-point enzymatic assay starting from 100 μM with 3-fold serial dilution against HDAC1 and HDAC6 (Reaction Biology Corp, Malvern, PA).

b SI (Selectivity Index) was calculated as the ratio between IC_50_ values of less sensitive HDAC isoforms (indicated in black) and the IC_50_ value of the most potently inhibited isoform (highlighted in blue). When more than one comparison was applicable, the SI is expressed as a range from the lowest to the highest calculated SI value.

c N.D.: not determined;

d N.A.: not active. Highlighted in blue: best IC_50_ observed for the compound.

To link the observed enzymatic inhibition with acetylated protein modulation in cells, the four glioma cell lines were exposed to the HDAC inhibitors **3a**, **6a**, and vorinostat at the tumor growth inhibition (TGI, 72 h) concentrations shown in Table 1 for 24 h. The accumulation of both acetylated-H3 and acetylated α-tubulin was determined as readout for HDAC activity. For inhibitors whose TGI concentration could not be determined, a concentration of 50 μM was applied. Vorinostat and **3a**, but not **6a**, significantly promoted the accumulation of acetyl-α-tubulin in three of the four cell lines tested, HOG, T98G, and U87MG, and only **3a** showed this effect also for U251MG cells (Figures 5 and S3C). It is important to emphasize that HDAC6 is the main player of α-tubulin deacetylation^37–39^ which is consistent with the potent HDAC6 inhibition by **3a**. However, at the concentrations used in the cellular assays, concomitant inhibition of class I HDACs is also expected, indicating that HDAC6 inhibition contributes to but does not solely determine the observed cellular effects.

**Figure 5:**
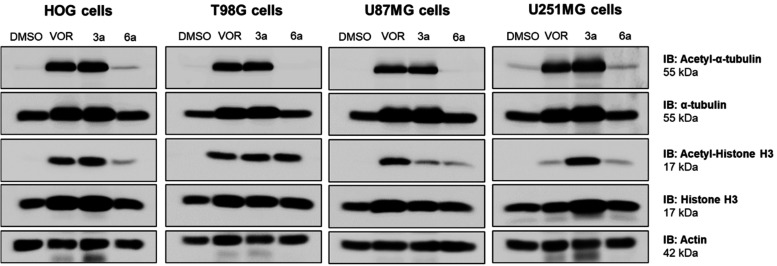
Effect of the HDAC inhibitors on specific acetylated substrates. Immunoblotting of acetyl α-tubulin, α-tubulin, acetyl-histone H3, and histone H3 proteins in glioma cells (HOG, T98G, U87MG, and U251MG) exposed to the HDAC inhibitors vorinostat (VOR), **3a**, and **6a** at TGI concentrations (72 h) (Table 1) for 24 h. For undetermined TGIs, a concentration of 50 μM was used.

Regarding acetyl-histone H3, all three compounds were significantly active, inhibiting histone H3 deacetylation in both HOG and T98G cells (Figures 5 and S3F). In U87MG cells, the pan-inhibitor was more effective in promoting acetyl-histone H3 accumulation, whereas the synthesized compounds showed greater activity in U251MG cells. Among them, **3a** and vorinostat were particularly effective in HOG and T98G, while vorinostat showed superior activity in U87MG, and **3a** showed superior activity in U251MG (Figure S3F).

Compound **6a** induced the accumulation of acetyl-histone H3 in HOG, T98G, and U251MG cell lines, with comparable effects of **3a** and vorinostat for these cells (Figures 5 and S3F). Studies indicated that HDAC2 activity may control H3K9ac levels during mitosis, suggesting its role in deacetylating this specific histone mark.^40^ During the remodeling process of chromatin, HDAC3 has an important role in removing acetyl groups on lysine 27 of histone H3, in which acetylation of 9 and 18 lysine residues can be observed with the knockdown of this HDAC.^6^ The similar accumulation profiles of acetylated histone H3 induced by **3a** and **6a** may be associated with their inhibition of HDAC2 and HDAC3 (Table 2 and Figure S2). Despite their distinct enzymatic selectivity profiles, these results indicate that overall cytotoxic activity at longer exposure times is largely associated with class I HDAC inhibition.

The expression level of different HDAC isoforms, available at the Human Protein Atlas^41^ for T98G, U87MG, and U251MG, shows the highest expression for HDAC1, and HDAC2 (Figure S4). Though the number of transcripts of HDAC1 and HDAC2 was higher in U251MG than in the two other cell lines (Figure S4A). Considering the heterogeneous HDACs expression profile and compound sensitivity among these cell lines, we hypothesize that there is a direct link between the observed antiproliferative effects and HDAC inhibition. Consistent with this hypothesis, the activity of compounds **3a** and **6a** on cells correlates with their inhibitory effects on HDACs 1–3.

Previous studies have shown that H3′s acetylation state is a marker for various malignancies. In the brain, H3 modification has a strong association with neurodegenerative and neuropsychiatric disorders,^42^ as well as gliomas of different grades.^43^ In brain cancer, both high and low levels of acetylated histone H3 can act as distinct biomarkers. For instance, low levels of H3K9ac were associated with worse prognosis in glioma patients and, conversely, increased H3K18ac acetylation correlated with better patient survival.^43,44^

In general, class I HDACs are overexpressed in various types of tumors, including gliomas, and are associated with the efficiency of the DNA repair process, making them an attractive target for chemotherapy.^45^ The analysis of HDAC expression levels from TCGA samples showed that HDAC1 and HDAC3 are more highly expressed in glioblastoma patients than in the normal tissues (Figure S4B). Nonetheless, HDAC6 can also play a relevant role in glioma patients, where this isoform is overexpressed.^46^ Recent studies highlight its importance in the proliferation of gliomas with isocitrate dehydrogenase 1 (IDH1) mutant isoform,^11^ as well as increased invasion and resistance to chemotherapy due to cells being more adapted to DNA damage.^47^

### 2.4 Compounds **3a** and **6a** promote cell cycle arrest and induce apoptosis in aggressive glioma cell lines

To understand the cytotoxic mechanisms behind the HDAC inhibition in the different glioma cell lines, **3a** and **6a** were subjected to further phenotypic analysis in glioma cells. Compounds **3a** and **6a** increased the percentage of cells in the subG1 phase in the T98G and U251MG cell lines, indicating DNA fragmentation and apoptosis, where **3a** (16.2 μM) was 3-fold more potent than **6a** (50 μM) in T98G (Figure 6 and Table 1). The subG1 population was not affected by any of the treatments in the HOG cell line. In addition to the increase in the number of cells in SubG1, vorinostat, **3a**, and **6a** also modulated the distribution of cells among the different phases of the cell cycle. The inhibitors preferentially modulated the G0/G1 and G2/M phases, except **6a**, which also modulated the S phase in U87MG cells.

**Figure 6:**
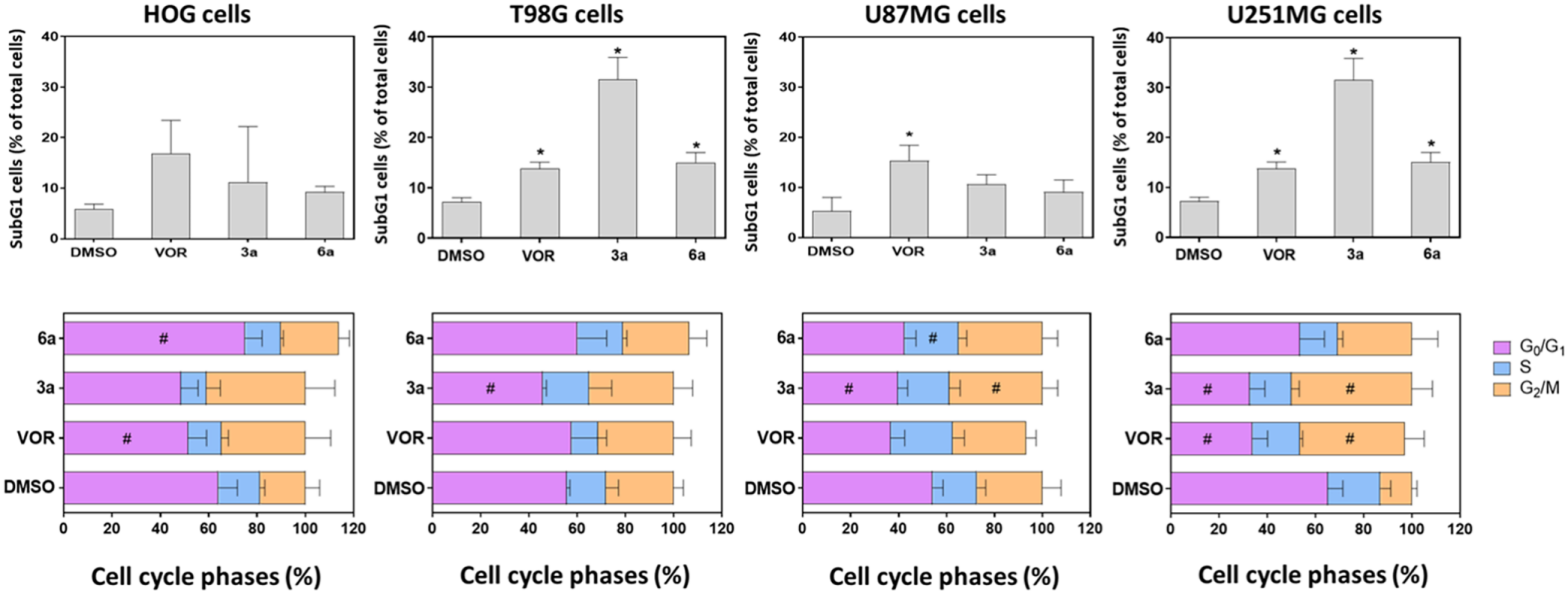
Effect of the different HDAC inhibitors on cell death and cell cycle progression. Comparative analysis of the percentage of cells in the SubG1 phase after treatment with the HDAC inhibitors vorinostat (VOR), **3a** and **6a** at TGI concentrations 72 h (Table 1), top panel, and cell cycle phase distribution of glioma cells treated with inhibitors for 24 h, bottom panel. ANOVA, Dunnett test, *, # *p* < 0.05, *n* = 3.

The cell cycle phases were also modulated by the inhibitors. In the U87MG and U251MG cell lines, treatment with compound **3a** resulted in a decrease in the G0/G1 and an increase in the G2/M phases, suggesting a shift in cell cycle progression. These observations suggest that a reduced number of cells are prepared for DNA synthesis but a larger population is blocked in the phase preceding cell division, leading to an accumulation of duplicated genetic material.

In contrast, treatment with compounds **3a** and **6a** in the HOG cell line resulted in the opposite effects. The first compound decreased the percentage of cells in the G0/G1 preparation phase, while the second compound increased the percentage of cells in this phase. Vorinostat modulated the G0/G1 phase in T98G and U251 cells and the G2/M phase only in U251MG cells.

In line with previous studies, vorinostat promotes G2/M phase arrest through the upregulation of p21 (CDKN1A) and the downregulation of cyclin D1 (CCND1) in breast cancer cells.^48^ The modulation of p21 post-treatment with vorinostat can be directly related to the decrease in the expression of this protein in cells that overexpress HDAC1,^49^ targeted by all three tested compounds.

To investigate the interference on apoptosis induction, Annexin V-positive cells were evaluated after vorinostat, **3a**, and **6a** treatments (Figure 7). Vorinostat and **3a** decreased the cell viability of all glioma cell lines. In HOG and U251 cells, an increase in apoptotic and necrotic cells was observed after treatment with **3a**, indicating an increased cytotoxicity of the compound. This might be related to the metabolism of the hydroxamic acid moiety, known to produce some toxic metabolites,^18^ which might drive the necrotic effects observed. Previous studies pointed out a link between HDAC4 and *p21* and *SHARP1* gene expression, suggesting that inhibiting this histone deacetylase can upregulate these genes, ultimately inducing apoptosis.^50–52^

**Figure 7:**
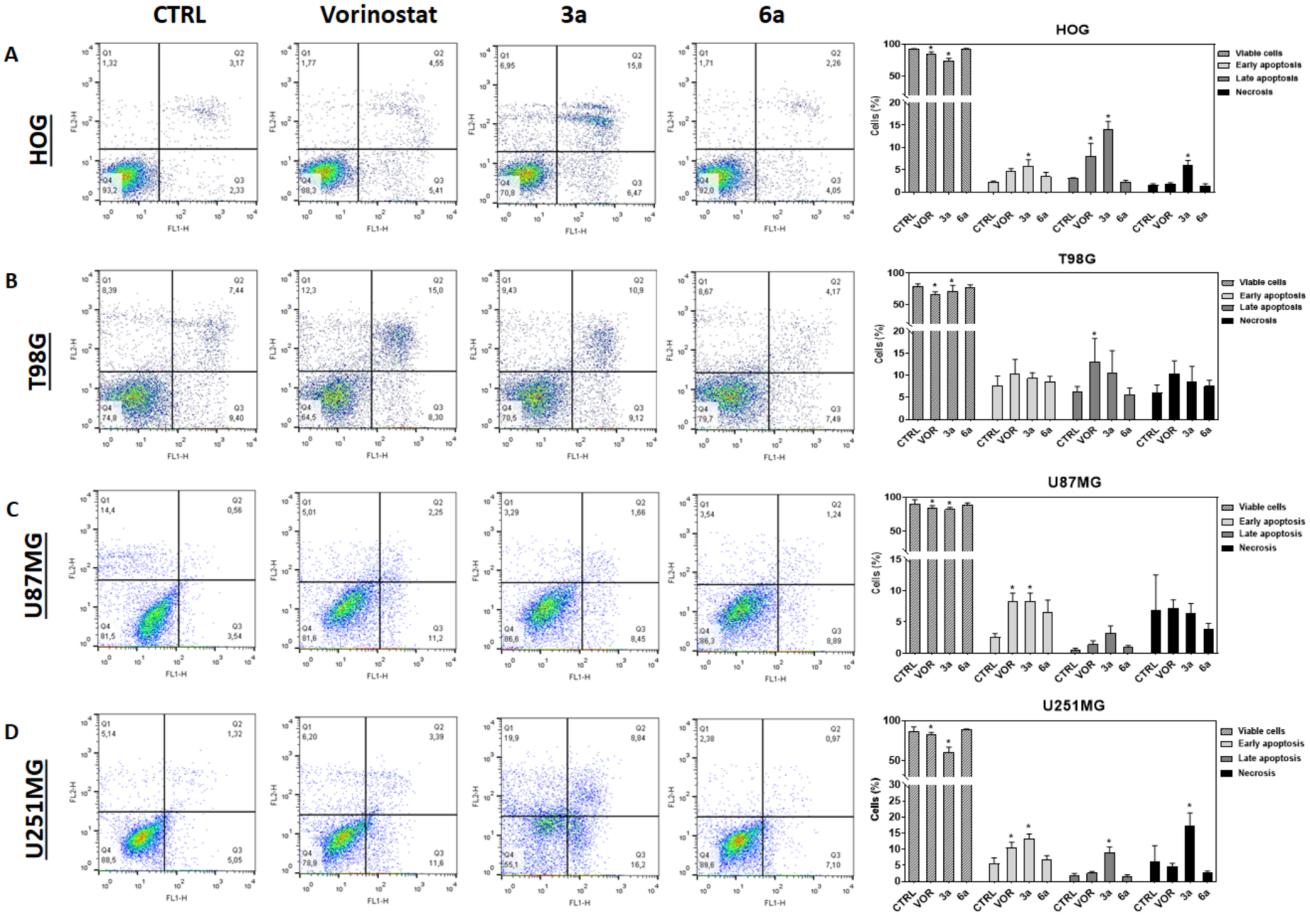
Apoptosis activation by the different HDAC inhibitors. Apoptosis detection was promoted by the HDAC inhibitors vorinostat, **3a**, and **6a** in glioma cells HOG (A), T98G (B), U87MG (C), and U251MG (D), treated with TGI concentrations 72 h (Table 1) for 24 h, followed by annexin V/propidium iodide (PI) staining. In the representative plots, apoptotic cells are annexin V-positive in quadrants 2 and 3 (Q2 and Q3). The bar graphs represent the mean ± SD of the quantification of viable, early apoptosis, late apoptosis, and necrosis cells. The *p*-values indicate *p < 0.05; ANOVA test and Bonferroni post-test were applied. *n* = 3.

### 2.5 Assessment of the cytotoxic potential of compounds **3a** and **6a** in glioblastoma stem cells (GSCs)

Glioblastoma stem cells (GSCs) play a relevant role in therapy resistance and lethality in this type of cancer.^53^ The two glioblastoma (GBM) subtypes, mesenchymal (MES) and proneural (PN), contain cells with distinct gene expression profiles, which allow for further subclassification of the tumor. In addition, the two subtypes also differ in proliferation rate and invasiveness, with mesenchymal cells exhibiting higher levels of both.^54^

The HDAC inhibitors were evaluated in previously generated GSC models^55^ in order to determine the cytotoxic potential of these compounds in cells considered to be the most aggressive and resistant cells in glioblastoma. Overall, the compounds exhibited very similar cytotoxicity in PN GSC23, with compound **6a** (1.8 μM) being slightly more potent than **3a** (3.7 μM). In contrast, in MES GG16, compounds **6a** and vorinostat showed comparable IC_50_ values (2.0 and 1.8 μM, respectively) and presented a potency more than 6 times higher than **3a** (IC_50_ 12.9 μM) (Figure 8 and Table 3), which leads us to hypothesize that HDAC1 can be an important player for this cell (Table 2).

**Figure 8:**
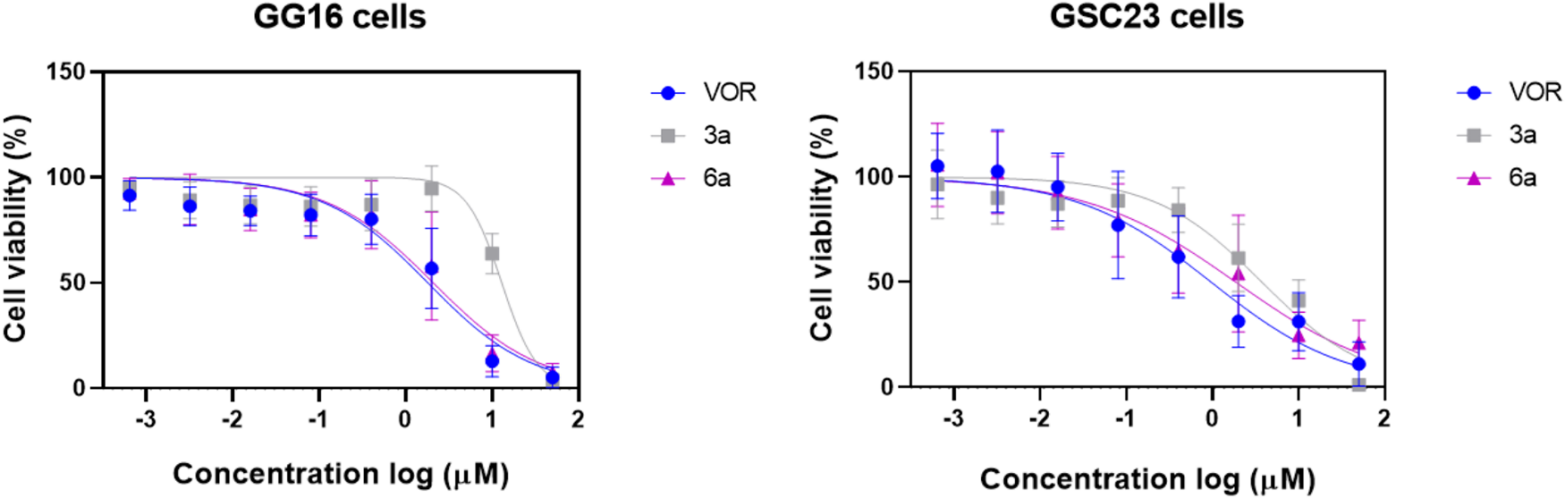
Cytotoxic effects of HDAC inhibitors on glioblastoma stem cells. Graphs of cell viability of GG16 and GSC23 (glioblastoma stem cells) exposed to compounds **3a** (gray), **6a** (purple), and vorinostat (VOR) (blue) at 72 h were measured by the MTS assay. Data correspond to the mean ± standard error of the mean from 3 independent experiments.

**Table 3:** Cytotoxic potential of HDAC inhibitors in glioblastoma stem cells^a^

	GG16 cells		GSC23 cells	
compounds	IC_50_	95% CI	IC_50_	95% CI
vorinostat	1.801	1.319–2.411	0.958	0.584–1.596
**3a**	12.90	N.D.–15.71	3.725	2.755–4.998
**6a**	2.079	1.478–2.870	1.851	1.142–3.046

a IC_50_ values and 95% confidence intervals (μM) of the HDAC inhibitors vorinostat, **3a**, and **6a** were tested in different subtypes of glioblastoma stem cells, GG16 and GSC23, for 72 h. N.D.: not determined.

Previous studies have shown that the expression of HDACs in GBM is associated with poor prognosis, different glioma grades,^8^ and resistance to anticancer therapy, particularly HDAC4, HDAC6, and HDAC8.^56,57^ Additionally, silencing of HDAC1 and HDAC2 has been shown to induce antiglioma effects.^9^ These findings indicate that distinct HDAC isoforms contribute differently to glioma biology, supporting the relevance of both class I HDACs and HDAC6 as complementary rather than hierarchical therapeutic targets. In this context, considering the possibility of targeting GSCs, which possess self-renewal, tumor-initiating potential, and resistance, HDAC inhibitors may be relevant drugs.^58–60^ They can promote the transcription of genes with apoptotic activity and also render DNA more vulnerable to damage since histone acetylation keeps the chromatin in a less condensed state.^61–63^

### 2.6 Compounds **3a** and **6a** present favorable interactions in their target HDAC isoforms

A combination of docking and molecular dynamics simulations was used to generate the proposed binding mode of compounds **3a** and **6a** in relevant HDAC enzymes (Figures 9A–E and S5–S7 and Tables S1 and S2). The simulations were conducted with **3a** and **6a** in the deprotonated form (Figures S8 and S9). Additionally, to rationalize the difference in potency among enzymes, the predicted binding energy using MM/GBSA (Figure 9G) was calculated, as well as the frequency of protein–ligand interactions along the simulation trajectory. The differences in binding energy against trichostatin A as reference ligand due to the availability of crystal (**TSA**, depicted in gray in these panels) and a benzamide representative (BNZ, Table S2) were also compared.

**Figure 9:**
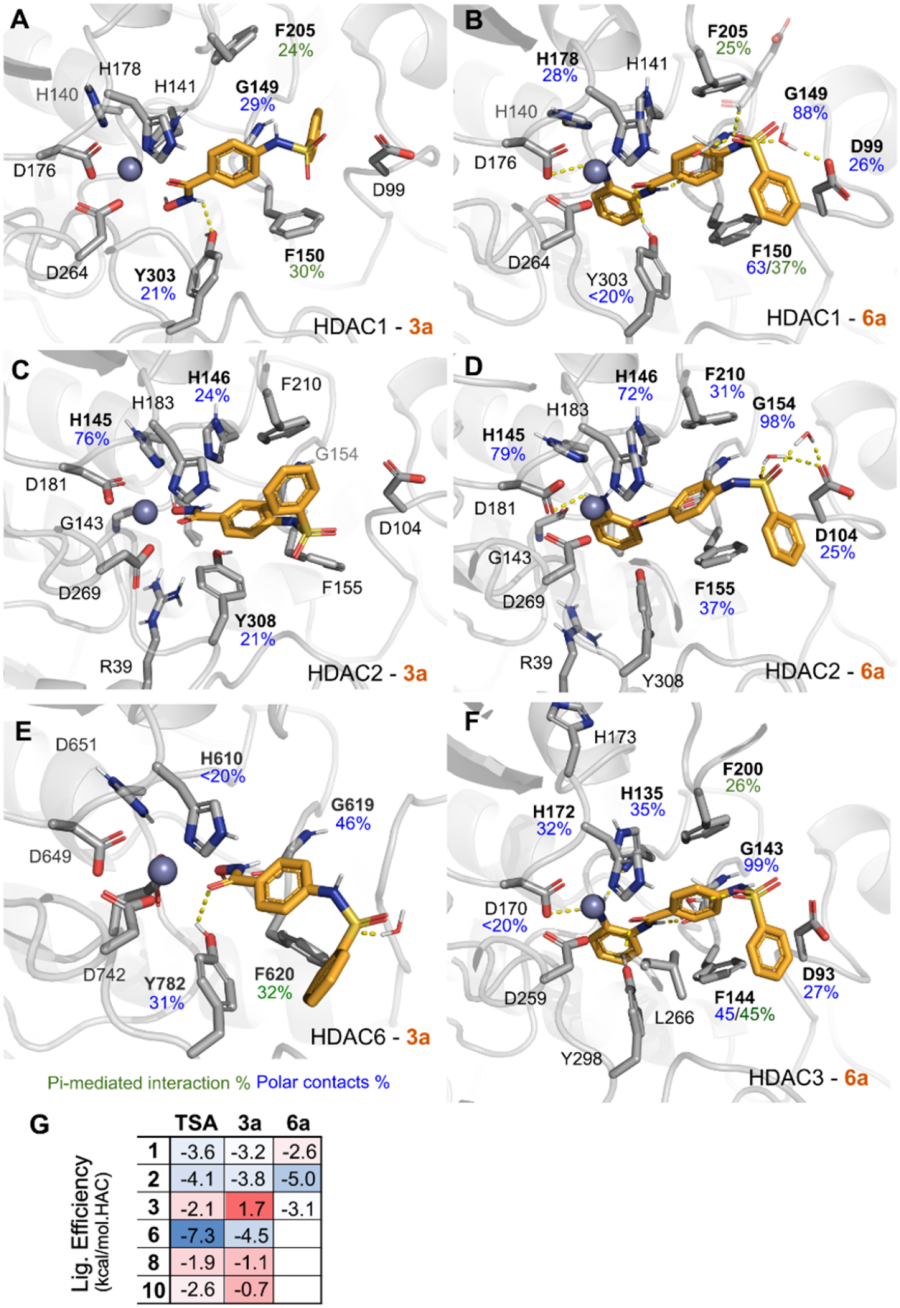
Proposed binding mode of compounds **3a** and **6a** in HDAC1, 2, 3 and 6. (A–F) Binding mode derived from a relevant frame of the MD simulations. Interaction frequencies (%) observed along the analyzed MD trajectories (5 × 100 ns) are indicated as numbers below their labels and colored as depicted in the figure legend. (G) Mean ligand efficiency prediction values for the HDAC simulations displayed as a heatmap. Ligand efficiency was calculated using MM/GBSA’s predicted binding energy (see Methods) and is represented by its mean + standard deviation (Table S2). HAC: heavy atom count, i.e. non-hydrogen atoms in the ligand. HAC can be used to normalize MM/GBSA calculations, accounting for differences in the molecular sizes. Data on other homologues are available as Supporting Information (Table S1 and Figures S5–S7).

HDAC6-selective phenyl hydroxamate inhibitors often display monodentate Zn^2+^ coordination modes,^64^ while HDAC6–**TSA** can have either bidentate (70% of the population) or monodentate (30%). For the purposes of comparability, we used bidentate conformations for all HDAC-ligands combinations. Docking of **3a** generated stable poses in all modeled HDACs, while docking of **6a** in HDAC6, 8, and 10 yielded no poses, most likely due to sterical constraints, due to the lack of a lower pocket or foot pocket to accommodate the benzamide moiety.^21^ Upon short simulations, ligand’s flexibility within the pocket had little changes (RMSD_ligand_ < 1 Å, Table S1) with the compound’s binding supported by stable interactions between their ZBG and intermittent contacts with the cap moieties.

A few exceptions, however, were observed. Although HDAC3-**3a** displayed multiple contacts, it exhibited a relatively poor predicted binding energy (i.e., dG: −6.7 kcal/mol). Also, HDAC8’s simulation revealed high ligand RMSD values, in particular, HDAC8-**3a** simulation had >2.20 Å, which together with the low interaction frequencies (only a transient interaction with G151 ∼ 27%) proposes a weak inhibition. In addition, HDAC10-**3a** simulations also showed poor binding (dG: −2.9 kcal/mol), which may explain the lower inhibitory potency of **3a** toward HDAC10 compared to HDACs class I.

Our hit compounds is stabilized by hydrophobic and pi-mediated interactions with the phenylamine residues along the binding channel (Figure 9). Comparing **3a** and **6a**’s interaction pattern on the different HDACs shows a slight advantage for the benzamide analogue, in regard to the number of Hbonds and binding energy, which can be expected since, this ZBG is able to explore the foot-pocket of these enzymes, while the hydroxamic derivative **3a**, lacks these contacts.^21^ On HDAC1–3, **6a** overcomes **3a** by 3–4 Hbonds with significantly lower binding energy for HDAC2 and 3 (Figure S7). This lower energy and higher H-bond count in **6a** was translated into a lower IC_50_ value for HDAC1 in comparison to **3a**. Compound **6a** benzamide’s amino group has stable Hbonds with the ZBG’s glycine (HDAC2: G154 and HDAC3: G143) for over 90% of the analyzed simulation time for most HDACs. Its cap group is also well positioned to interact with the surface aspartate residues (HDAC1: D99, HDAC2: D104, and HDAC3: D93). Both interactions are absent on **3a**’s simulations.

Benzamide ZBG interactions support **6a**’s HDAC class I selectivity, but other interactions are key to confer isoform specificity.^21^ Although similar interactions were observed between **6a** and HDAC1–3, it seems there is a difference between how a glycine interacts with the inhibitor in the isoforms. In HDAC2–3, G154/143 interacted with the ZBG, while in HDAC1, wherein the lowest IC_50_ was observed, this interaction occurred with the linker moiety, which could account for the increased affinity observed.

Of note, the time scale of our simulations is adequate for HDAC descriptions, as relevant interactions are consistently reproducible with control ligand’s simulations and literature.^27^ However, our pipeline’s major limitation is the extensive use of classical force-fields, which disregard the change in bond orders and ligand’s polarizability, for the energy prediction. This could be the reason for the poor precise correlation between predicted binding energies and biochemical activity.

### 2.7 In silico analysis suggests **3a** and **6a** present favorable pharmacokinetic properties

Although *in silico* modeling techniques still face limitations, due to the quality and structural diversity of the data set where the models were trained, they still are valuable tools to guide optimization efforts of the drug design programs.^65^ In this sense, to further explore the potential of compounds **3a** and **6a** as glioma treatment candidates, the preliminary pharmacokinetic properties were assessed *in silico* using the PhaKinPro tool.^66^ Both compounds were predicted to present central nervous system (CNS) activity, Caco-2 permeability, and subcellular half-lives above 30 min, all with greater than 69% confidence (Table 4), indicating a favorable pharmacokinetic profile. Other parameters were predicted with lower statistical confidence, but were consistent with trends reported in the literature.^15^ Specifically, the benzamide **6a** was predicted to be stable in plasma (*t*_1/2_ > 12 h), and to exhibit minimal plasma protein binding, while the hydroxamic acid derivative **3a** was predicted to bind to plasma proteins, and to present low plasmatic stability (*t*_1/2_ < 1 h) (Table 4).

**Table 4:** Preliminary pharmacokinetic properties predicted for **3a** and **6a**

	compounds	
pharmacokinetic property	**3a**	**6a**
hepatic stability	no prediction (out of applicability domain)	>50% at 60 min
		confidence: 54.53%
microsomal *t*_1/2_ tissue	>30 min	≤30 min
	confidence: 53.0%	confidence: 55.0%
microsomal *t*_1/2_ subcellular	>30 min	>30 min
	confidence: 85.0%	confidence: 79.0%
microsomal intrinsic clearance	<12 μL/min/mg	<12 μL/min/mg
	confidence: 72.4%	confidence: 58.0%
renal clearance	between 0.50 and 1.00 mL/min/kg	no prediction (out of applicability domain)
	confidence: 60.8%	
plasma *t*_1/2_	half-life below 1 h	>12 h
	confidence: 51.2%	confidence: 50.1%
plasma protein binding	plasma protein binder	poor protein binder
	confidence: 68.4%	confidence: 62.4%
oral bioavailability	between 0.5 and 0.8 F	between 0.5 and 0.8 F
	confidence: 52.27%	confidence: 51.47%
Caco-2	does permeate Caco-2	does permeate Caco-2
	confidence: 69.2%	confidence: 70.8%
BBB^a^ permeability	does not permeate BBB	does not permeate BBB
	confidence: 52.8%	confidence: 55.2%
CNS^b^ activity	does exhibit CNS activity	does exhibit CNS activity
	confidence: 94.0%	confidence: 91.2%

a BBB: blood–brain barrier.

b CNS: central nervous system.

Both compounds were predicted not to permeate the blood–brain barrier (BBB) with 55% confidence. Fragment-based contribution maps indicate that the phenyl-sulfonamide cap region does not significantly contribute to permeability (Figure 10A), suggesting that structural modifications at this position could be explored to improve BBB passage. In particular, the introduction of small and moderately lipophilic substituents, such as short alkyl chains on the sulfonamide nitrogen, could increase lipophilicity while maintaining an acceptable log *D* range and is expected to preserve key interactions with the HDAC active site, as the cap region primarily mediates surface recognition rather than zinc coordination. In addition, the hydroxamic acid moiety of compound **3a** showed negative contributions to permeability in both BBB and Caco-2 cell models, whereas the benzamide scaffold of compound **6a** displayed neutral or favorable contributions (Figure 10A,B). This observation suggests that, beyond contributing to isoform selectivity, the increased lipophilicity of the benzamide moiety may also favor permeability and could partially account for the higher cytotoxic activity of compound **6a** in glioma stem cells compared to **3a**.

**Figure 10:**
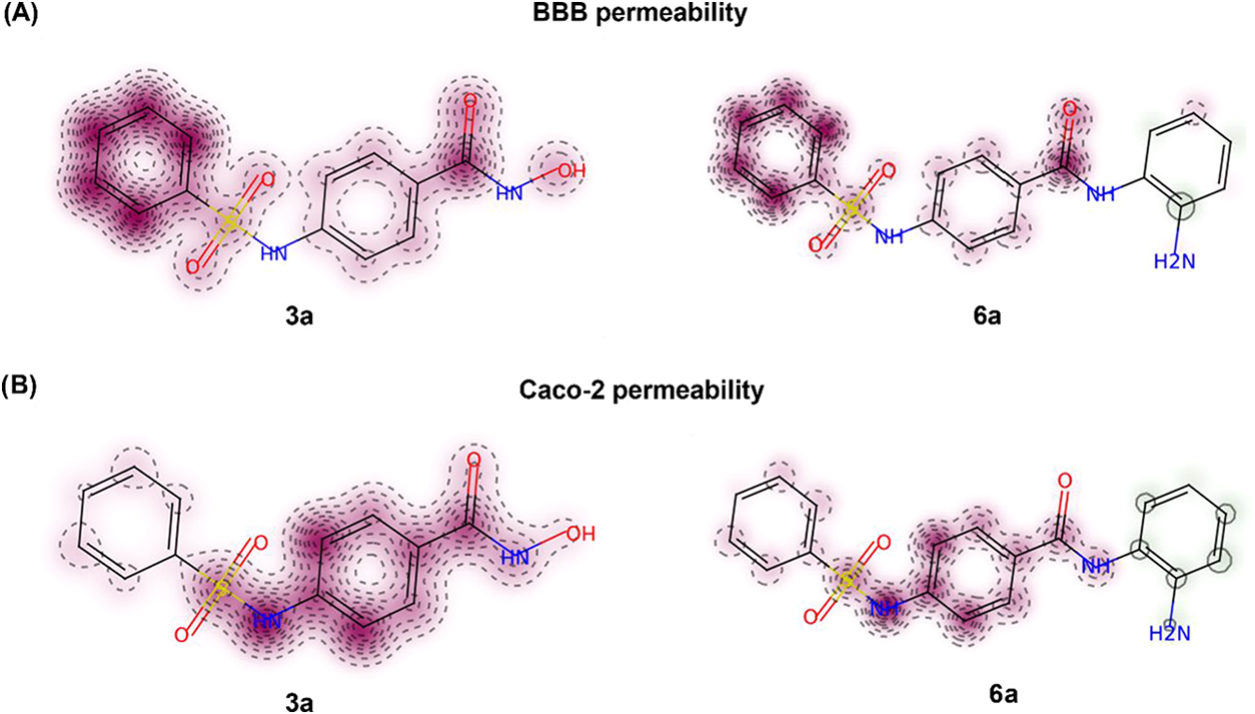
Fragment-based contribution maps of **3a** and **6b** for permeability predictions in BBB (A) and Caco-2 cells (B). Red indicates areas that contribute to low permeability predictions, while green shows areas that contribute to increased permeability predictions.

## 3 Conclusions

Novel HDAC inhibitors for glioma treatment bearing a phenyl-sulfonamide cap and different ZBGs were designed and synthesized. Compounds with *para* linker substitution presented higher cytotoxicity in cancer cells, with hydroxamic acid **3a** and benzamide **6a** being the most promising inhibitors, able to induce apoptosis in four different cell lines. Mechanistically, both compounds induced cell cycle arrest and apoptosis, with **3a** notably increasing the sub-G1 population and promoting late apoptosis and necrosis in aggressive glioma models. Furthermore, both compounds showed efficacy in glioblastoma stem cells, with **6a** displaying superior potency across mesenchymal and proneural subtypes. The observed cytotoxicity was linked to a distinct HDAC inhibition profile, with **3a** inhibiting HDAC6 together with class I HDACs, and **6a** preferentially inhibiting HDAC1/3. Overall, the *in vitro* enzymatic results agreed with pharmacodynamic experiments, which showed that while **3a** inhibits both tubulin and histone deacetylation, **6a** selectively inhibits the deacetylation of histone H3 in T98G. These results were further supported by molecular docking and MD/simulation, which revealed stable profiles consistent with the observed isoform selectivity. Compound **6a** formed persistent hydrogen bonds with amino acid residues in class I HDACs, explaining its higher affinity and selectivity for HDAC1/3. In contrast, compound **3a** exhibited strong binding affinity for HDAC6 through Zn^2+^ coordination and hydrophobic interactions along the activity site, while showing limited interaction with HDAC10, and class I HDACs. *In silico* models suggest the compounds present favorable pharmacokinetic properties, rendering them hits for the treatment of gliomas, suitable for future developments in the field.

## 4 Materials & Methods

### 4.1 Chemistry

Solvents were purified according to standard procedures. Reagents and solvents were purchased from Synth, Merck, Sigma-Aldrich, and Oakwood Chemicals. Reactions were monitored by TLC on Merck silica gel (60 F 254) by using UV light (λ = 254 nm) and iodine as visualizing agents and ninhydrin, bromocresol green, ferric chloride, or molybdite staining solutions. The compounds were purified by recrystallization, precipitation, or column chromatography, either traditional or with the automated chromatography system BIOTAGE, Isolera Prime model, or SNAP Ultra C18 BIOTAGE column. ^1^H and ^13^C NMR spectra were obtained on a 300/75 MHz Bruker spectrometer, using the solvent residual peak as the internal reference (chemical shifts: DMSO-*d*_6_, 2.50/39.52). Analytical HPLC was carried out on a Shimadzu Proeminence instrument under the following conditions: column, C-18 Gemini (5 μm, 150 × 4.6 mm); mobile phase, 5–100% H_2_O/CH_3_CN containing 0.1% TFA at a flow rate of 1.0 mL/min for 25 min; UV detection at 254 nm. The purities of all tested compounds were >95%, as determined by analytical HPLC.

#### 4.1.1 General Procedure A

In a round-bottom flask, 6 mmol of methyl 4-aminobenzoate (0.91 g, 1.2 equiv) was dissolved in 10 mL of THF (2 mL/mmol). To this solution, 5 mL of H_2_O was added (1 mL/mmol), and the pH was adjusted to 9.0 by adding 2 mL of saturated Na_2_CO_3_. The system was placed in an ice bath, and once the temperature reached 0 °C, 5 mmol of benzenesulfonyl chloride (0.631 mL, 1 equiv) was added dropwise. The reaction medium was stirred at room temperature, maintaining the pH at 9.0 until completion. Afterward, the THF was evaporated, and the mixture was dissolved in 30 mL of EtOAc. The pH was adjusted to 1.0, and the organic phase was washed with 5% HCl (3 × 10 mL) and dried over MgSO_4_. The solvent was evaporated, forming either **1a** as a pure solid, or additionally purified by column chromatography with a gradient elution of EtOAc-hexane, obtaining **1b** as a solid.

##### 4.1.1.1 Methyl 4-(Phenylsulfonamido)­benzoate (**1a**)

The test compound was prepared according to General Procedure A from methyl 4-aminobenzoate and phenysulfonyl chloride and isolated as a white solid in 79% yield. ^1^H NMR (300 MHz, DMSO-*d*_6_) δ 10.84 (bs,1H), 7.84–7.80 (m, 4H); 7.60–7.55 (m, 3H), 7.23 (d, *J* = 8.7 Hz, 2H), 3.77 (s, 3H) (Figure S10). ^13^C NMR (75 MHz, DMSO-*d*_6_) δ 165.6, 142.3, 139.2, 133.2, 130.6 (2C), 129.4 (2C), 126.6 (2C), 124.4, 118.2 (2C), 51.9 (Figure S11). Purity: 99% (254 nm) (Figure S36).

##### 4.1.1.2 Methyl 3-(Phenylsulfonamido)­benzoate (**1b**)

The test compound was prepared according to General Procedure A from methyl 3-aminobenzoate and phenysulfonyl chloride, followed by isolation as a yellow solid in 94% yield. ^1^H NMR (300 MHz, DMSO-*d*_6_) δ 10.53 (bs, 1H), 7.77 (d, *J* = 7.1 Hz, 2H), 7.71 (s, 1H), 7.61–7.54 (m, 4H), 7.38–7.37 (m, 2H), 3.81 (s, 3H) (Figure S12). ^13^C NMR (75 MHz, DMSO-*d*_6_) δ 166.1, 139.7, 138.7, 133.5, 131.0, 130.2, 129.8 (2C), 127.1 (2C), 125.2, 124.9, 120.8, 52.7 (Figure S13). Purity: 97% (254 nm) (Figure S37).

#### 4.1.2 General procedure B

In a round-bottom flask, 3 mmol of the esters **1a**–**b** (1 equiv) and 10 mL of a solution of KOH (sat.) were added (3.3 mL KOH/mmol of ester, 6.6 equiv). The system was placed under agitation and reflux (100 °C). After the reaction was concluded, the reaction mixture was cooled at room temperature and placed in an ice bath. The pH was then adjusted to 1.0, with HCl 2 M. The suspension was then transferred to a beaker, heated, and stirred until all of the precipitate was dissolved, adding the minimum amount of H_2_O if necessary to aid solubility. The solution was cooled to room temperature and then set in the freezer for 1h. The crystals were filtered under vacuum, washed with cold H_2_O (3 × 5 mL), and dried under vacuum to obtain products **2a**–**b** as solids.

##### 4.1.2.1 4-(Phenylsulfonamido)­benzoic Acid (**2a**)

The test compound was prepared according to General Procedure B from **1a** as a white solid in 85% yield. ^1^H NMR (300 MHz, DMSO-*d*_6_) δ 12.70 (bs, 1H), 10.78 (bs, 1H), 7.84–7.79 (m, 4H), 7.65–7.54 (m, 3H), 7.20 (d, *J* = 8.7 Hz, 2H) (Figure S14). ^13^C NMR (75 MHz, DMSO-*d*_6_) δ 166.7, 141.9, 139.3, 133.1, 130.7, 129.4 (2C), 126.6 (2C), 125.7, 118.2 (2C) (Figure S15). Purity: 99% (254 nm) (Figure S38).

##### 4.1.2.2 3-(Phenylsulfonamido)­benzoic Acid (**2b**)

The test compound was prepared according to General Procedure B from **1b** as a white solid in 95% yield. ^1^H NMR (300 MHz, DMSO-*d*_6_) δ 12.99 (bs, 1H), 10.49 (bs, 1H), 7.76 (dt, *J* = 7.9; 1.1 Hz, 2H), 7.70 (s, 1H), 7.63–7.52 (m, 4H), 7.36–7.34 (m, 2H) (Figure S16). ^13^C NMR (75 MHz, DMSO-*d*_6_) δ 166.7, 139.3, 138.0, 133.0, 131.7, 129.5, 129.3 (2C), 126.6 (2C), 124.8, 124.1, 120.6 (Figure S17). Purity: 97% (254 nm) (Figure S39).

#### 4.1.3 General Procedure C

In a round-bottom flask containing 8.0 mmol of NaOH (0.32 g, 8.0 equiv) was placed in an ice bath. When the temperature reached 0 °C, 50 mmol of hydroxylamine in an aqueous solution (3.23 mL NH_2_OH 50% p/v, 50.0 equiv) was added to dissolve the NaOH. To that, a solution containing 1.0 mmol of intermediate **1a**–**b** (0.291g, 1.0 equiv) dissolved in 6 mL (6 mL/mmol) of tetrahydrofuran and methanol (THF:MeOH, 1:1) was added dropwise. The mixture was kept under agitation at room temperature until the reaction was completed. Afterward, the mixture was extracted with EtOAc (3 × 15 mL), washed with brine, and evaporated to obtain products **3a**–**b** as solids.

##### 4.1.3.1 *N*-Hydroxy-4-(phenylsulfonamido)­benzamide (**3a**)

The final compound was prepared according to General Procedure C from **1a** as a white solid in 47% yield. ^1^H NMR (300 MHz, DMSO-*d*_6_) δ 11.02 (bs, 1H), 10.63 (bs, 1H), 8.91 (bs, 1H), 7.81 (d, *J* = 6.9 Hz, 2H), 7.64–7.53 (m, 5H), 7.14 (d, *J* = 6.9 Hz, 2H) (Figure S18). ^13^C NMR (75 MHz, DMSO-*d*_6_) δ 164.2, 140.8, 139.9, 133.6, 129.8 (2C), 128.6 (2C), 128.4, 127.1 (2C), 119.0 (2C) (Figure S19). Purity: 99% (254 nm) (Figure S40).

##### 4.1.3.2 *N*-Hydroxy-3-(phenylsulfonamido)­benzamide (**3b**)

^1^H NMR (300 MHz, DMSO-*d*_6_) δ (ppm): 11.14 (br, 1H), 10.43 (br, 1H), 7.76 (d, *J* = 6.9 Hz, 2H), 7.63–7.58 (m, 1H), 7.56–7.53 (m, 2H), 7.52 (m, 1H), 7.36–7.34 (m, 1H), 7.28 (t, *J* = 7.5 Hz, 1H), 7.23–7.21 (m, 1H) (Figure S20). ^13^C NMR (75 MHz, DMSO-*d*_6_) δ (ppm): 163.6, 139.3, 137.9, 133.9, 132.9, 129.2 (2C), 129.1, 126.5 (2C), 122.3, 122.0, 119.0 (Figure S21). Purity: 96% (254 nm).

#### 4.1.4 General Procedure D

In a round-bottom flask, 1.0 mmol of esters **1a**–**b** (0.291 g, 1.0 equiv) was dissolved in 1 mL of MeOH (1 mL/mmol). The system was cooled to 0 °C in an ice bath, followed by the addition of 64.3 mmol of NH_2_NH_2_ in aqueous solution (2.0 mL of NH_2_NH_2_ 50% p/v, 64.3 equiv). After homogenization, the system was placed under agitation and reflux until the reaction was completed. The system was left open for a few minutes to allow partial concentration and then cooled to room temperature. 3 mL of water was added, and the reaction mixture was refrigerated at 0 °C for 2 h. The resulting white precipitate was filtered under a vacuum, washed with H_2_O (3 × 5 mL), and dried under a vacuum for approximately 4 h. The obtained solid was transferred to a dried round-bottom flask and dissolved in 10 mL of anhydrous EtOH (10 mL/mmol). The solution was cooled to 0 °C in an ice bath, and 1.0 mmol of salicylaldehyde (105 μL, 1.0 equiv) was added dropwise, followed by a single drop of glacial acetic acid. The reaction mixture was stirred at room temperature until complete consumption of salicylaldehyde was achieved. The solvent was evaporated, the mixture resuspended in 20 mL of EtOAc, washed with saturated NaHSO_3_ solution (3 × 10 mL), dried over MgSO_4_, filtered, and concentrated under reduced pressure. The product was purified by recrystallization from DCM with a minimum amount of MeOH, yielding **4a**–**b** as solids.

##### 4.1.4.1 (*E*)-*N*-(4-(2-(2-Hydroxybenzylidene)­hydrazine-1-carbonyl)­phenyl)­benzenesulfonamide (**4a**)

The final compound was prepared according to General Procedure D from **1a** as a white solid in 67% yield. ^1^H NMR (300 MHz, DMSO-*d*_6_) δ 11.95 (bs, 1H), 11.28 (bs, 1H), 10.76 (bs, 1H), 8.58 (s, 1H), 7.87–7.81 (m, 4H), 7.64–7.50 (m, 3H), 7.30–7.23 (m, 3H), 6.94–6.91 (m, 2H) (Figure S22). ^13^C NMR (75 MHz, DMSO-*d*_6_) δ 162.2, 157.4, 148.1, 141.1, 139.3, 133.2, 131.3, 129.5, 129.4 (2C), 129.0 (2C), 127.7, 126.7 (2C), 119.3, 118.6, 118.4 (2C), 116.4 (Figure S23). Purity: 95% (254 nm) (Figure S41).

##### 4.1.4.2 (*E*)-*N*-(3-(2-(2-Hydroxybenzylidene)­hydrazine-1-carbonyl)­phenyl)­benzenesulfonamide (**4b**)

The final compound was prepared according to General Procedure D from **1b** as a white solid in 56% yield. ^1^H NMR (300 MHz, DMSO-*d*_6_) δ 12.04 (bs, 1H), 11.22 (bs, 1H), 10.52 (bs, 1H), 8.61 (s, 1H), 7.80–7.78 (m, 2H), 7.67 (s, 1H), 7.60–7.55 (m, 5H), 7.42–7.37 (t, *J* = 7.8 Hz, 1H), 7.32–7.27 (m, 2H), 6.95–6.89 (m, 2H) (Figure S24). ^13^C NMR (75 MHz, DMSO-*d*_6_) δ 162.3, 157.4, 148.6, 139.3, 138.2, 133.9, 133.0, 131.4, 129.5, 129.3 (2C), 126.6 (2C), 123.1, 122.8, 119.6, 119.3 (2C), 118.6, 116.4 (Figure S25). Purity: 96% (254 nm) (Figure S42).

#### 4.1.5 General Procedure E

In a dried round-bottom flask, 1.0 mmol of the carboxylic acid intermediate **2a**–**b** (0.277g, 1 equiv) and 1.2 mmol of EDC (0.230 g, 1.2 equiv) were dissolved in 10 mL of dry DCM. To this solution, 0.5 mmol of DMAP (61 mg, 0.5 equiv) and 1 mmol of 3-amino-1,2,4-triazole (84 mg, 1.0 equiv) were added, and the reaction mixture was stirred at room temperature until the starting materials were consumed. The solvent was evaporated, and the mixture was dissolved in 20 mL of EtOAc, washed with a buffered solution (NaOAc/AcOH) at pH 5.4 (3 × 10 mL), dried over MgSO_4_, and concentrated. The desired product was isolated by reverse-phase column chromatography in a gradient of elution of MeOH-H_2_O with 1%, yielding **5a**–**b** as solids.

##### 4.1.5.1 4-(Phenylsulfonamido)-*N*-(4*H*-1,2,4-triazol-3-yl)­benzamide (**5a**)

The final compound was prepared according to General Procedure E from **2a** as a white solid in 42% yield. ^1^H NMR (300 MHz, DMSO-*d*_6_) δ 10.94 (bs, 1H), 8.00 (d, *J* = 8.8 Hz, 2H), 7.87 (d, *J* = 7.7 Hz, 2H), 7.66–7.56 (m, 5H), 7.24 (d, *J* = 8.8 Hz, 2H) (Figure S26). ^13^C NMR (75 MHz, DMSO-*d*_6_) δ 166.6, 158.3, 151.2, 142.2, 139.3, 133.2, 132.6 (2C), 129.5 (2C), 126.7 (2C), 126.3, 117.2 (2C) (Figure S27). ESI HRMS calc. for C_15_H_13_N_5_O_3_S: [M + H]^+^, *m*/*z* 344.080. Value found 344.081. Purity: 97% (254 nm) (Figure S43). The ^1^H and ^13^C NMR signal assignments were supported by ^1^H–^13^C HSQC (HETCOR) and HMBC experiments (Figures S28 and S29).

##### 4.1.5.2 3-(Phenylsulfonamido)-*N*-(4*H*-1,2,4-triazol-3-yl)­benzamide (**5b**)

The final compound was prepared according to General Procedure E from **2b** as a white solid in 40% yield. ^1^H NMR (300 MHz, DMSO-*d*_6_) δ 10.40 (bs, 1H), 7.75 (d, *J* = 7.6 Hz, 2H), 7.60–7.51 (m, 4H), 7.31–7.34 (m, 2H), 7.14 (d, *J =* 8.4 Hz, 1H), 7.03 (s, 1H) (Figure S30). ^13^C NMR (75 MHz, DMSO-*d*_6_) δ 169.3, 139.2, 137.6, 137.3, 135.4, 133.0, 129.3 (2C), 128.9, 126.6 (2C), 122.5, 120.1, 119.7, 118.3 (Figure S31). Purity: 95% (254 nm) (Figure S44).

#### 4.1.6 General Procedure F

In a dried round-bottom flask, 1.0 mmol of the carboxylic acid intermediate **2a**–**b** (0.277g, 1 equiv) and 1.2 mmol of EDC (0.230 g, 1.2 equiv) were dissolved in 10 mL of dry DCM. To this solution, 0.5 mmol of DMAP (61 mg, 0.5 equiv) and 1 mmol of 1,2-diaminebenzene (0.108g, 1.0 equiv) were added, and the reaction mixture was stirred at room temperature until the starting materials were consumed. The solvent was evaporated, and the mixture was dissolved in 20 mL EtOAc, washed with a buffered solution (NaOAc/AcOH) at pH 5.4 (3 × 10 mL), dried over MgSO_4_, and concentrated. The desired product was isolated by reverse-phase column chromatography in a gradient of elution of MeOH-H_2_O with 1%, yielding **6a**–**b** as solids.

##### 4.1.6.1 *N*-(2-Aminophenyl)-4-(phenylsulfonamido)­benzamide (**6a**)

The final compound was prepared according to General Procedure F from **2a** as a white solid in 64% yield. ^1^H NMR (300 MHz, DMSO-*d*_6_) δ 10.68 (bs, 1H), 9.48 (bs, 1H), 7.86–7.82 (m, 4H), 7.63–7.57 (m, 3H), 7.21 (d, *J* = 8.5 Hz, 2H), 7.11 (d, *J* = 7.6 Hz, 1H), 6.95 (t, *J* = 7.4 Hz, 1H), 6.76 (d, *J* = 7.8 Hz, 1H), 6.57 (t, *J* = 7.4 Hz, 1H), 4.83 (s, 2H) (Figure S32). ^13^C NMR (75 MHz, DMSO-*d*_6_) δ 164.5, 143.1, 140.6, 139.4, 133.1, 129.7, 129.4 (2C), 129.0 (2C), 126.64 (2C), 126.59, 126.4, 123.3, 118.3 (2C), 116.2, 116.0 (Figure S33). Purity: 95% (254 nm) (Figure S45).

##### 4.1.6.2 *N*-(2-Aminophenyl)-3-(phenylsulfonamido)­benzamide (**6b**)

The final compound was prepared according to General Procedure F from **2a** as a white solid in 45% yield. ^1^H NMR (300 MHz, DMSO-*d*_6_) δ 10.50 (bs, 1H), 9.60 (bs, 1H), 7.76 (d, *J* = 6.8 Hz, 2H), 7.70 (s, 1H), 7.63–7.59 (m, 4H), 7.37–7.33 (m, 2H), 7.16 (d, *J* = 7.6 Hz, 1H), 6.98 (t, *J =* 7.6 Hz, 1H), 6.80 (d, *J* = 7.9 Hz, 1H), 6.60 (t, *J* = 7.5 Hz, 1H), 4.85 (s, 2H) (Figure S34). ^13^C NMR (75 MHz, DMSO-*d*_6_) δ 164.8, 143.0, 139.5, 138.1, 135.7, 132.9, 129.3 (2C), 129.0, 126.6 (2C), 126.52, 126.48, 123.2, 122.9, 122.5, 119.7, 116.2, 116.1 (Figure S35). Purity: 96% (254 nm) (Figure S46).

### 4.2 Cell Culture and Compounds

Gliomas cells HOG, T98G, U87MG, and U251MG were cultivated in Roswell Park Memorial Institute (RPMI) 1640 medium supplemented with Fetal Bovine Serum (10% v/v) and Penicillin-Streptomycin (10,000 U/mL). Glioblastoma stem cells (GSCs), GG16, and GSC23, were cultivated in Neurobasal-A Medium supplemented with B-27 (2% v/v), Glutamine (1% v/v), Fibroblast Growth Factor basic (20 ng/mL), and Epidermal Growth Factor human (20 ng/mL). All cells were maintained in a cell incubator at 37 C, 5% CO_2_.

Vorinostat (suberoylanilide hydroxamic acid, SAHA) was acquired from Cayman Chemical, and all tested compounds were diluted in DMSO.

#### 4.2.1 Cytotoxic Assays

Sulforhodamine B assay (SRB)^67^ was applied to evaluate the cytotoxic potential of HDAC inhibitors in HOG, T98G, U87MG, and U251MG. Cells were resuspended in supplemented RPMI medium at a density of 1 × 10^4^ cells/mL, except for U251MG, whose density was 2 × 10^4^ cells/ml. 200 μL of cell solution was seeded per well in 96-well flat-bottom plates for treated and nontreated plates. After 24 h the substances were applied, and nontreated plate had the medium removed; the cells were fixed with trichloroacetic acid (10% w/v), and this plate was maintained at 4 °C until the last exposure time, 72h.

Treated plates received substances in different concentrations, ranging from 0.0032–50 μM for three different times of exposure, 24, 48, and 72 h. After a specific time, media were removed and the plates were maintained at 4 °C at least 4h before next steps. All plates with fixed cells were washed with demi water, stained with SRB acid acetic solution (4% w/v), and incubated at 37 °C, 30 min. The staining was removed, and wells were washed with acid acetic solution of 1%. SRB dye in each well was diluted in Trisbase 10 mM, and absorbance was read in a multimode microplate reader (Agilent Biotek, Santa Clara, CA) at 510 nm.

The cytotoxic evaluation in GSCs was performed by the MTT method,^68^ using MTS solution (CellTiter 96 AQueous One Solution Cell Proliferation Assay, Promega). GSCs cells were plated in 96-well flat-bottom plates at 10 × 10^4^ cells/ml. Vorinostat and compounds **3a** and **6a** were applied in concentrations from 0.0032 to 50 μM. After 72 h of exposure, 20 μL of MTS solution was added in each well, and the plates were left for 2 h in cell incubator (37 °C, 5% CO_2_) protected from light. Wells absorbance was measured at 490 nm in a MultiSkan Go Microplate Spectrophotometer (Thermo Fisher Scientific, Waltham, MS, USA).

### 4.3 In vitro HDACs inhibition assays

Histone deacetylase (HDAC) inhibition assays were conducted by Reaction Biology Corp. (Malvern, PA) using purified, full-length human recombinant HDAC1 and HDAC6 enzymes expressed in Sf9 insect cells via a baculovirus system. The fluorogenic substrate RHKK­(Ac)-AMC, derived from p53 residues 379–382, was used to monitor enzymatic activity. Reactions were performed in a buffer containing 50 mM Tris-HCl (pH 8.0), 127 mM NaCl, 2.7 mM KCl, 1 mM MgCl_2_, 1 mg/mL BSA, and 1% DMSO (final concentration). Test compounds were dissolved in DMSO and preincubated with the enzyme for 5–10 min prior to substrate addition. The reaction mixtures were then incubated for 2 h at 30 °C. The reactions were quenched by the addition of Trichostatin A, followed by a developer solution to induce fluorescence. Dose–response curves were obtained from 10-point, 3-fold serial dilutions starting at 100 μM. IC_50_ values were calculated from these curves and represent the average of duplicate determinations.

### 4.4 Cell cycle and apoptosis analysis

Glioma cells were evaluated through flow cytometry to identify cell cycle changes and apoptotic cells. HOG, T98G, and U87MG were seeded at 1 × 10^4^ cells/mL, and U251MG at 2 × 10^4^ cells/mL in a 60-mm cell culture dish in 10% FBS-containing RPMI-1640 medium in the presence of DMSO or HDAC inhibitors (vorinostat, **3a**, and **6a**). Compounds were applied in the cells according to TGI concentrations of 72 h (Table 1) for 24 h.

For the cell cycle, cells were fixed in EtOH 70% and stained with a buffer containing Triton 0.1%, propidium iodide (10 μg/mL), and RNase A (100 μg/mL). For apoptosis analysis, cells were previously washed with ice-cold phosphate-buffered saline (PBS) and resuspended in a binding buffer containing propidium iodide (1 μg/mL) and APC-labeled annexin V (1 μg/mL) and incubated for 15 min at room temperature in a light-protected area. Ten thousand events were acquired for each sample and analyzed by flow cytometry (FACSCalibur; Becton-Dickinson, San Jose, CA, USA).

### 4.5 Western blot

Protein extraction was performed using a buffer containing 10 mM Na_3_VO_4_, 100 mM NaF, 10 mM Na_4_P_2_O_7_, 100 mM Tris (pH 7.6), 1% Triton X-100, 2 mM PMSF, and 4 mM EDTA. The same amount of protein from each sample was subjected to polyacrylamide gel electrophoresis followed by SDS-PAGE. Proteins were transferred to a nitrocellulose membrane and then subjected to an antibody solution. Antibodies against acetyl-α-tubulin (Lys40) (D20G3) (no. 5335), α-tubulin (DM1A) (no. 3873), acetyl-histone H3 (Lys9/Lys14) (no. 9677), histone H3 (no. 4499), and β-actin (13E5) (no. 4970) were obtained from Cell Signaling Technology (Danvers, MA).

### 4.6 Molecular modeling

#### 4.6.1 Model generation and structure preparation

We modeled the systems with Maestro (Schrödinger Release 2024.4 Maestro, Schrödinger, LLC, New York, NY, 2024) and the OPLS4 force field,^69^ unless otherwise stated. Models were generated individually (as specified in Table S2), and a missing side chain of inserted residues was placed using Prime, followed by loop refinement using the same software.^70^ *N*-terminus, but not the C-terminus of each model, was capped. The proteins were prepared using Protein Preparation Wizard (Schrödinger LLC, New York, NY, 2024). Missing hydrogen atoms were added, bond orders were assigned using the CCD database, and protonation states of amino acids were optimized with PROPKA (Schrödinger, LLC, New York, NY, 2024) at pH 7.4 in the Protein Preparation Wizard tool of Maestro, to select the most likely protonation states and tautomer for the histidine residues. We agreed with the software suggestions, followed by optimizing the generated H-bonding species. Finally, each structure was globally minimized using the steepest descent method (cutoff: 0.5 Å for all atoms). For each combination of HDAC-ligand, one representative model structure was selected for further analysis.

#### 4.6.2 Molecular docking and pose selection

Before docking, ligands were prepared using LigPrep (Schrödinger, LLC, New York, NY, 2024) to assign the protonation state (Epik; at pH 7.4 ± 1.0) and the partial charges. Isomers’ chiral center configurations were retrieved from the literature using their respective CAS numbers. The starting configuration for HDAC-bound systems was generated using docking (Glide v7.7 ^71,72^ ), with default settings. Docking was conducted using standard precision (SP), without any interaction restriction and keeping other options, such as van der Waals interactions, penalties for unsatisfied hydrogen bonds, and grid size as their default values. Redocking results were satisfactory, displaying RMSD values <1.5 Å. Whenever possible, HDAC’s ligands and Zn^2+^ coordination were set as bidentate using half-bonds.

#### 4.6.3 Molecular dynamics simulations

We used the Desmond MD simulation engine^73^ and the OPLS4 force-field.^69^ Ligand charges and parameters were generated for the OPLS4 directly during the system preparation using their respective force-field builder tool (Maestro2024v4). The prepared systems were solvated in a cubic box with the size of the box set as a 13 Å minimum distance from the box edges to any atom of the protein. TIP3P water model^74^ was used to describe the solvent, and the net charge was neutralized using Na^+^ ions. The RESPA integrator timesteps of 2 fs for bonded and near and 6 fs for far were applied. The short-range Coulombic interactions were treated using a cutoff value of 9.0 Å, whereas long-range Coulombic interactions were estimated using the Smooth Particle Mesh Ewald (PME) method.^75^ Before the production simulations, systems were relaxed by using the default Desmond relaxation protocol. Briefly, Maestro′s Desmond implementation has a default relaxation protocol that starts with two stages of energy minimization (backbone restrained and unrestrained) followed by four stages of MD runs with gradually diminishing those restraints, which compose an automated multistage equilibration process. It minimizes solute atoms with restraints, while solvent molecules and ions are relaxed around the solute. Next, it minimizes the full system without restraints to remove residual strain. With the NVT ensemble (constant volume and constant temperature), a short MD simulation runs at low temperature (10 K) with solute restraints. This helps to gradually heat the solvent without disrupting the solute structure. It continues gradual heating to the target temperature (310 K) under an NPT (constant pressure, constant temperature) ensemble, while maintaining positional restraints. This step allows the solvent density to adjust to controlled conditions. Full relaxation under NPT conditions without restraints happens next (see www.deshawresearch.com *’s* Desmond manual for the details). For production, Simulations were run in NPT ensemble with a temperature of 310 K (using the Nosé-Hoover thermostat^76,77^ ) and pressure of 1.01325 bar (Martyna-Tobias-Klein barostat^78^). For each system, five independent simulations of at least 100 ns were carried out, resulting in 500 ns of simulation data for each system. Each replica was generated using the same initial coordinates but randomly generated seed numbers for equilibration and production.

#### 4.6.4 MM/GBSA binding energy calculations

Molecular mechanics with generalized Born and surface area (MM/GBSA) predicts the binding free energy of protein–ligand complexes using Prime.^79^ In this sense, every 10th frame from the simulations was considered for the calculations, meaning 50–60 frames. These were used as input files for the MM/GBSA calculations with the thermal_mmgbsa.py script for the Schrödinger package. Calculated free-binding energies (kcal/mol) are represented by MM/GBSA and normalized by the number of heavy atoms (HAC), according to the following formula: Ligand Efficiency = (binding energy)/(1 + Ln­(HAC)) for ligand efficiency.

#### 4.6.5 Visualization and plotting

Structural data visualization was conducted with PyMOL version 2.5.2 (Schrodinger LLC, New York, NY, USA). Data visualization was also completed by Python 3.7, seaborn (v0.12.2), matplotlib,^80^ and GraphPad Prism (v. 10.3 for Windows, GraphPad Software, San Diego, CA, USA).

#### 4.6.6 Data and Software Availability Statement

All prepared structures, molecular dynamics (MD) trajectories, simulation configuration, and parameter files, as well as raw and processed data related to HDAC–ligand interactions, are available through the Zenodo repository under the DOI: 10.5281/zenodo.15297801 (accessible upon publication). Third-party software used in this study includes: GraphPad Prism version 10.2 (https://www.graphpad.com/), Schrödinger Suite 2024.3–2025.1 (https://www.schrodinger.com), and PyMOL version 2.5.2–3.1 (https://pymol.org/), each distributed under its respective license.

##### 4.6.6.1 p*K*_a_ prediction

The acid–base ionization behavior of compounds **3a** and **6a** was evaluated by *in silico* prediction of p*K*_a_ values and corresponding ionization curves using the Chemicalize^81^ web platform, developed by ChemAxon (https://chemicalize.com). The calculations were performed by using the built-in p*K*_a_ prediction engine, and ionization curves were generated over a physiologically relevant pH range. The resulting p*K*_a_ profiles for compounds **3a** and **6a** are shown in Figures S8 and S9, respectively.

##### 4.6.6.2 PK properties prediction

The pharmacokinetic properties of the test compounds and their associated fragment-contribution maps were assessed using the Pharmacokinetics Profiler (PhaKinPro) web tool, available at https://phakinpro.mml.unc.edu/. The detailed description of the model’s development and validation is described in the literature.^66^

## Supplementary Material



## Data Availability

The data underlying this study are available throughout the manuscript and Supporting Information.

## Notes

Simulation data; HDAC’s chosen structures or models employed and their truncated sequence positions; screening of HDAC inhibitors data; enzymatic inhibition data assay; quantitative protein expression of HDAC inhibition markers, HDACs expression in glioblastoma cells lines and patient samples; p*K*_a_ curves for compounds **3a** and **6a**; potential binding mode of **3a** and **6a** within HDACs1–3; potential binding mode of **3a** within HDACs6, 8 and 10; cumulative distributions of ligand efficiency along the simulations; NMR spectra of compounds; HPLC spectra of purified compounds (PDF)
