# Supplementary material for: Repositioning HDAC Inhibitors for Glioma Treatment: Synthesis and Biological Evaluation
Source: ACS Omega. 2026 Feb 3;11(6):10104–20. doi: 10.1021/acsomega.5c11083 (PMC12917657; doi:10.1021/acsomega.5c11083)
Supplement: Supplementary file 1 [file ao5c11083_si_001.pdf]

Repositioning HDAC Inhibitors for Glioma Treatment: Synthesis and Biological Evaluation

Authors: Luciana Costa Furtado,[a,b] Karoline de Barros Waitman,[c] Nuno Silva,[c] Leticia Marcelino Gouvea,[a] Thales Kronenberger,[d,e] Mônica Franco Zannini Toledo,[c] Elthon Gois Ferreira,[a] João Agostinho Machado-Neto,[a] Frank Kruyt,[b] Roberto Parise Filho,[c*] Letícia V. Costa-Lotufo[a*]

[a]Department of Pharmacology, Institute of Biomedical Sciences, University of São Paulo, Avenida Professor Lineu Prestes 1524, 05508-000, São Paulo, Brazil.

[b]Department of Medical Oncology, University Medical Center Groningen, University of Groningen, DA11, Postbus 30.001, 9700 RB, Groningen, The Netherlands.

[c]Department of Pharmacy, Faculty of Pharmaceutical Sciences, University of São Paulo, Av. Prof. Lineu Prestes, 580, 05508-000, São Paulo, Brazil.

[d]Interfaculty Institute of Microbiology and Infection Medicine (IMIT), University of Tübingen, Tübingen, Germany; Partner-site Tübingen, German Center for Infection Research (DZIF), 72076, Tübingen, Germany

[e]School of Pharmacy, Faculty of Health Sciences, University of Eastern Finland, P.O. Box 1627, FI-70211 Kuopio, Finland.

*costalotufo@usp.br; *roberto.parise@usp.br

Contents

Figure S1 - Screening of HDAC inhibitors against glioma cell lines HOG and T98G. 5

Figure S2 - Evaluation of the potential of the enzymatic inhibition of 3a and 6a against HDACs. ..6

Figure S3 - Quantitative protein expression of HDAC inhibition markers. 7

Figure S4 - HDACs expression in glioblastoma cells lines and patient samples. 8

Figure S5 - Potential binding mode of 3a and 6a within HDACs1-3. 9

Figure S6 - Potential binding mode of 3a within HDACs6, 8 and 10. 10

Figure S7 - Cumulative distributions of ligand efficiency along the simulations. 11

Figure S10 - 1H NMR spectra of final compound 1a 12

Figure S11 - 13C NMR spectra of the final compound 1a 13

Figure S12 - 1H NMR spectra of final compound 1b 14

Figure S13 - 13C NMR spectra of the final compound 1b 15

Figure S14 - 1H NMR spectra of final compound 2a 16

Figure S15 - 13C NMR spectra of the final compound 2a 17

Figure S16 - 1H NMR spectra of final compound 2b 18

Figure S17 - 13C NMR spectra of final compound 2b 19

Figure S18 - 1H NMR spectra of final compound 3a 20

Figure S19. 13C NMR spectra of final compound 3a 21

Figure S20 - 1H NMR spectra of final compound 3b 22

Figure S21 - 13C NMR spectra of final compound 3b 23

Figure S22 - 1H NMR spectra of final compound 4a 24

Figure S23 - 13C NMR spectra of final compound 4a 25

Figure S24 - 1H NMR spectra of final compound 4b 26

Figure S25 - 13C NMR spectra of final compound 4b 27

Figure S26 - 1H NMR spectra of final compound 5a 28

Figure S27 - 13C NMR spectra of final compound 5a 29

Figure S28 - Hetcor 13C / 1H NMR spectra of final compound 5a 30

Figure S29 - HMBC 1H / 13C NMR spectra of final compound 5a 31

Figure S30 - 1H NMR spectra of final compound 5b 32

Figure S31 - 13C NMR spectra of final compound 5b 33

Figure S32 - 1H NMR spectra of final compound 6a 34

Figure S33 - 13C NMR spectra of final compound 6a 35

Figure S34 - 1H NMR spectra of final compound 6b 36

Figure S35 - 13C NMR spectra of final compound 6b 37

Figure S36 - HPLC spectra of the final compound 1a. 38

Figure S37 - HPLC spectra of the final compound 1b. 39

Figure S38 - HPLC spectra of the final compound 2a. 40

Figure S39 - HPLC spectra of the final compound 2b. 41

Figure S40 - HPLC spectra of the final compound 3a. 42

Figure S41 - HPLC spectra of the final compound 4a. 43

Figure S42 - HPLC spectra of the final compound 4b. 44

Figure S43 - HPLC spectra of the final compound 5a. 45

Figure S44 - HPLC spectra of the final compound 5b. 46

Figure S45 - HPLC spectra of the final compound 6a. 47

Figure S46 - HPLC spectra of the final compound 6b. 48

Figure S9 - pKa curves for compounds 6a...50

Figure S8 - pKa curves for compounds 3a...49

Table S1 - Summary of the simulation's data...51

Table S2 - HDAC's chosen structures or models employed in this study and their truncated sequence positions. 53

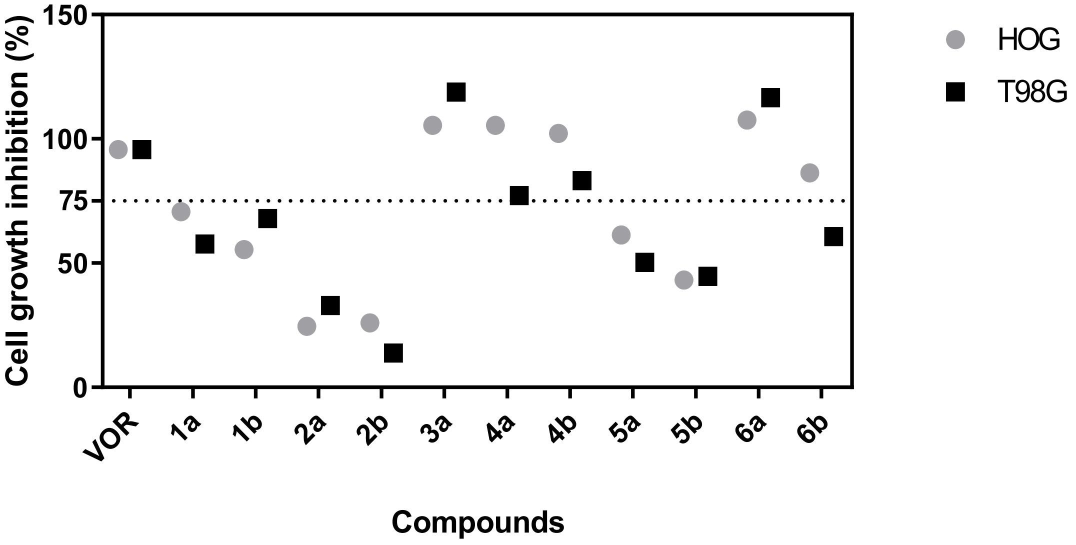

Figure S1 - Screening of HDAC inhibitors against glioma cell lines HOG and T98G. Cells were exposed to the inhibitors for 72 h and their growth was evaluated by the SRB assay to evaluate potential cell growth inhibition of compounds. Cells treated with DMSO and vorinostat (VOR) were used as negative and positive controls, respectively. Results analisis were performed using GraphPad Prism v.10 software from four independent experiments ($n \geq 4$).

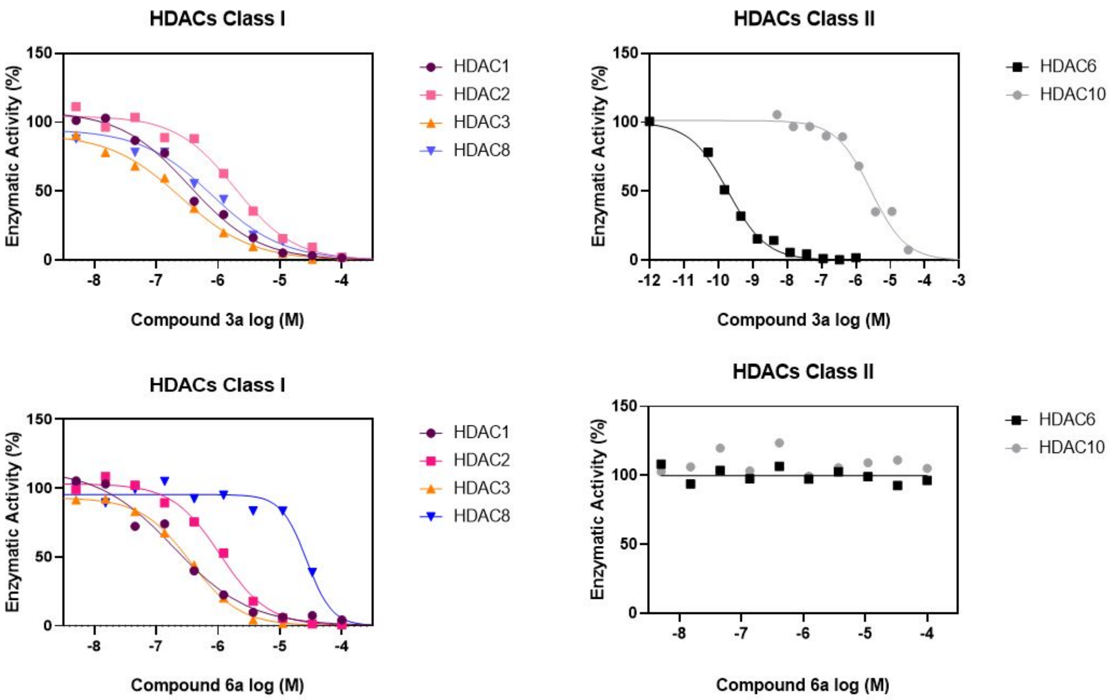

Figure S2 - Evaluation of the potential of the enzymatic inhibition of 3a and 6a against HDACs. Dose response curve of the enzymatic activity (%) of HDACs class II (1, 2, 3, and 8) and HDACs class II (6 and 10) treated with different concentrations, ranging from $5x10^{-11}$ to $1x10^{-4}$, of inhibitors **3a** and **6a** for 2h 30min at 30 °C. Fluorogenic peptide from p53 residues 379-382 (RHKK(Ac)AMC) was used for HDACs 1, 2, 3, and 6, fluorogenic peptide from p53 residues 379-382 (RHK(Ac)K(Ac)AMC) was used for HDAC 8, and Ac-Spermidine-AMC was used for HDAC10.

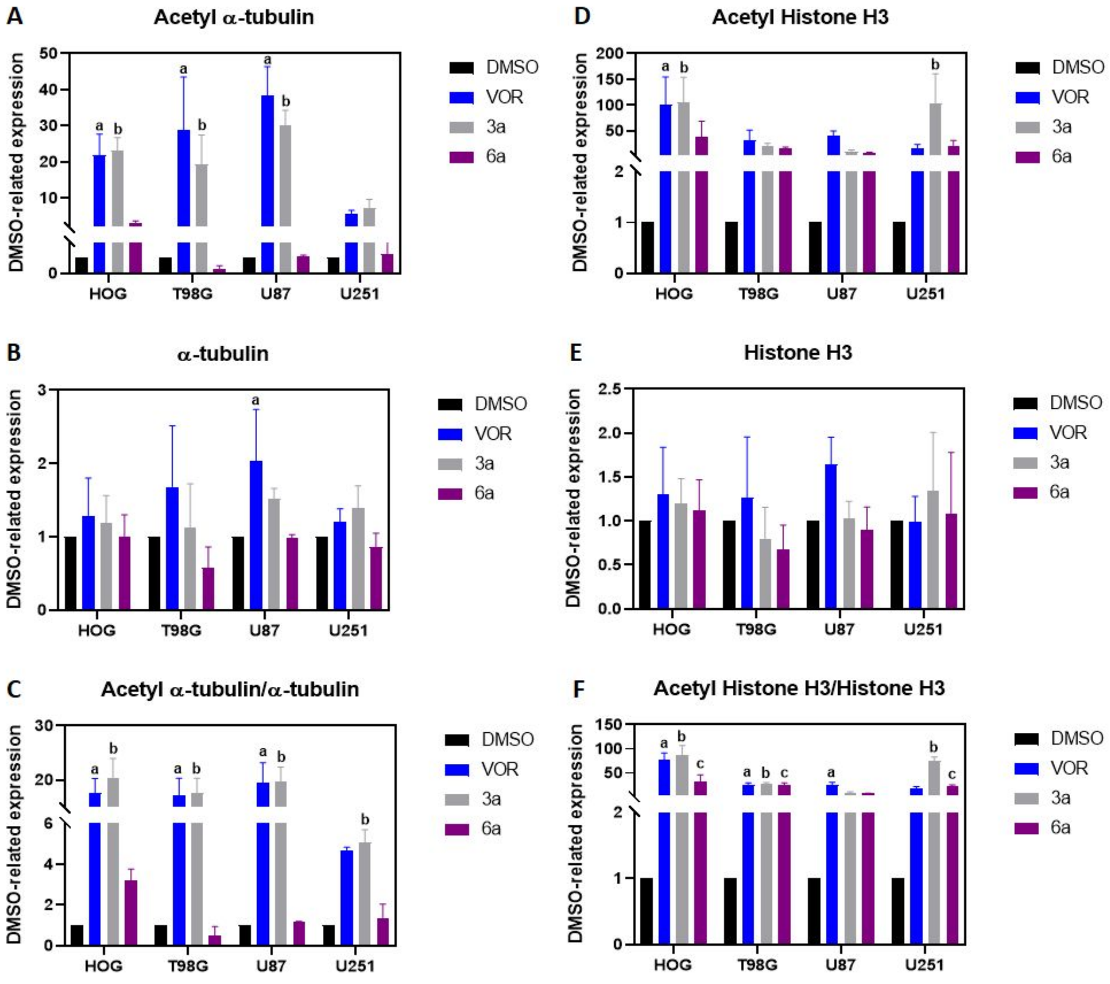

Figure S3 - Quantitative protein expression of HDAC inhibition markers. Comparison of the relative expression of acetyl α-tubulin (**A**), α-tubulin (**B**), acetyl α-tubulin/α-tubulin (**C**), acetyl-histone H3 (**D**), histone H3 (**E**), and acetyl-histone H3/histone H3 (**F**) proteins in glioma cells (HOG, T98G, U87, U251) treated with DMSO (negative control), VOR (vorinostat), 3a, and 6a at TGI concentrations (72 h) (Table 1) for 24 h. Protein quantification was performed using immunoblot images from three independent experiments analyzed with UN-SCAN-IT gel software version 6.1. Analysis of variance (2-way ANOVA) followed by Dunnett's test, with $p \leq 0.05$, represented by a, b, and c when compounds were compared to negative control (DMSO).

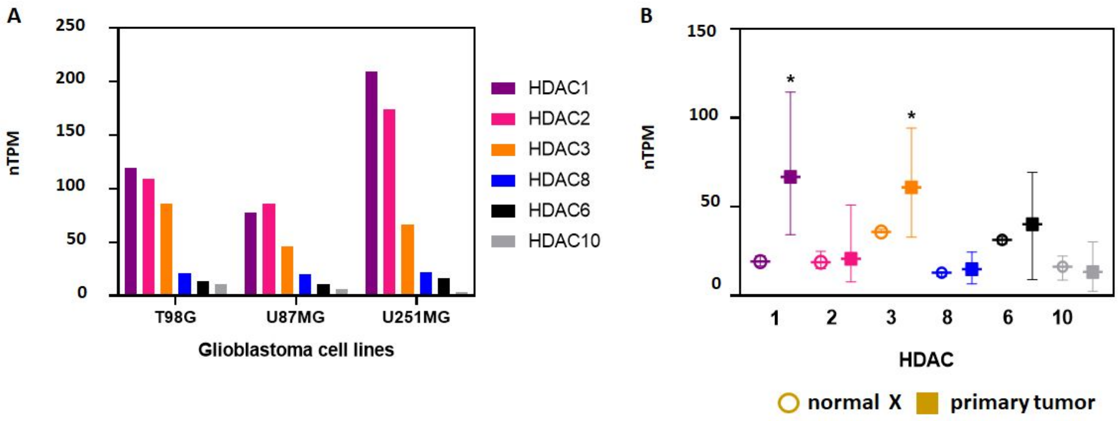

Figure S4 - HDACs expression in glioblastoma cells lines and patient samples. Comparative transcipts analysis of HDACs from glioblastoma cell lines, T98G, U87MG, and U251MG (data collected and adapted from The Human Protein Atlas) (A), and normal and primary tumor, glioblastoma (data collected and adapted from ualcan.path.uab.edu, TCGA databank) (B). nTPM (number of transcripts per million)

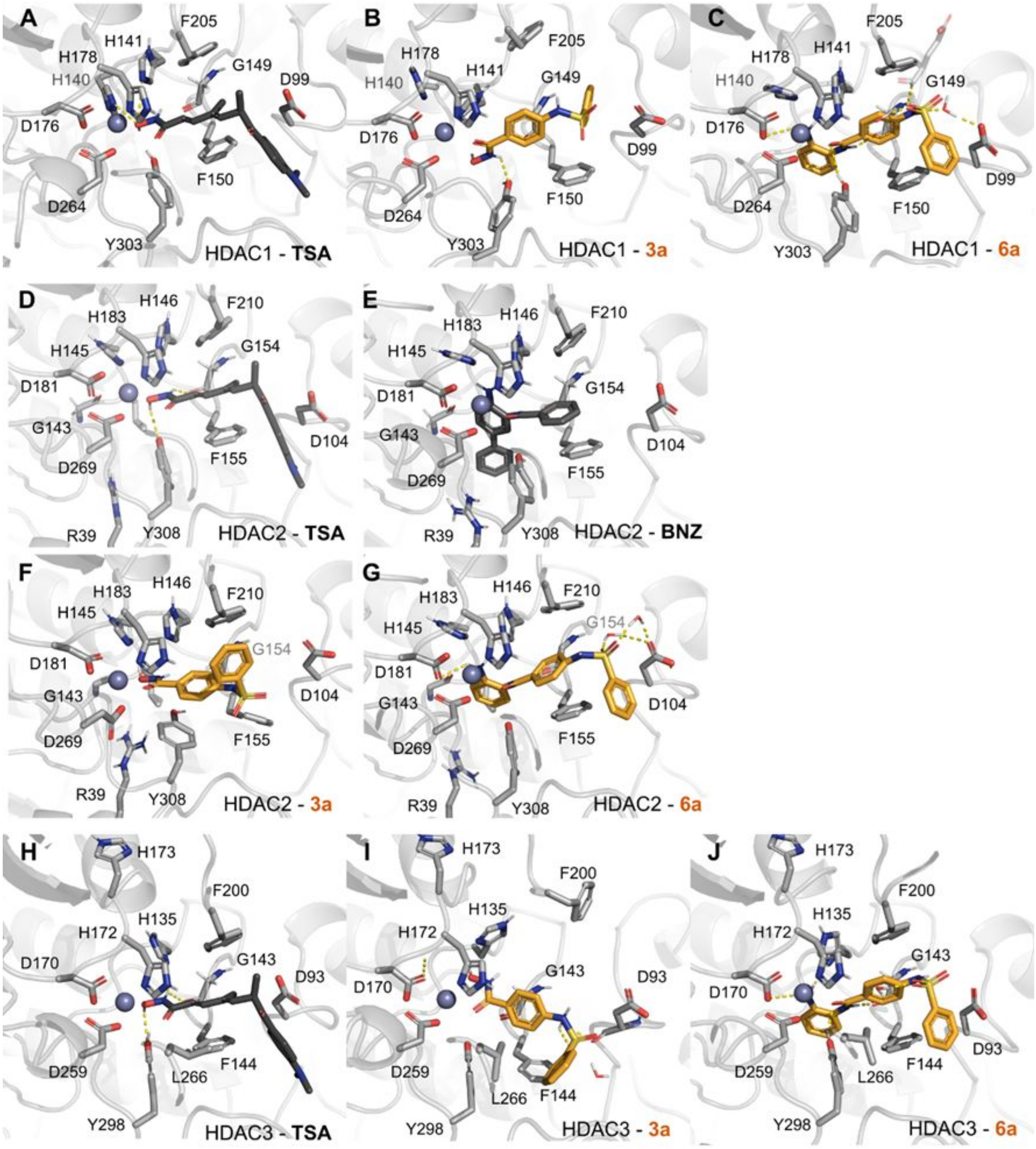

Figure S5 - Potential binding mode of 3a and 6a within HDACs1-3. Representative structures from the simulations obtained from hierarchical clustering of the trajectories for HDAC1 (A-C), HDAC2 (D-G) and HDAC3 (H-J) with the TSA (A,D,I) or BNZ (E) control ligands or our hits: 3a (B, F, I, in orange) or 6a (C, G, J, also in orange). Polar contacts are depicted as yellow dashed lines. Protein-ligand interaction frequency for each residue was quantified and available in Table S2.

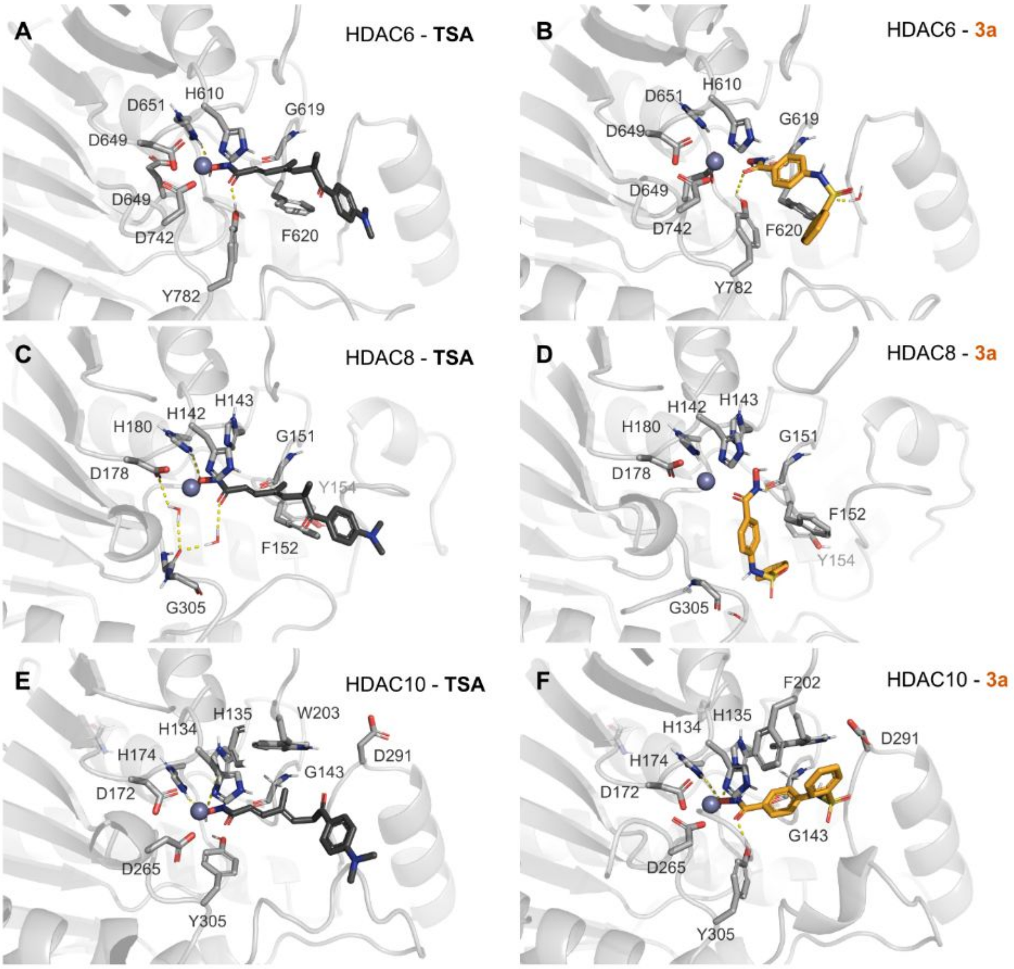

Figure S6 - Potential binding mode of 3a within HDACs6, 8 and 10. Representative structures from the simulations obtained from hierarchical clustering of the trajectories for HDAC6 (A,B), HDAC8 (C,D) and HDAC10 (E,F) with the TSA (A,C,E) control ligand or our hit 3a (B, C, F, in orange). Polar contacts are depicted as yellow dashed lines. Protein-ligand interaction frequency for each residue was quantified and available in **Table S2**

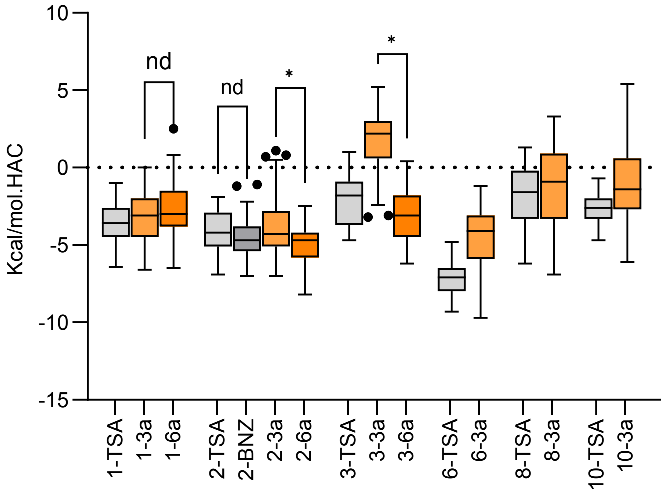

Figure S7 - Cumulative distributions of ligand efficiency along the simulations. Ligand efficiency was calculated using MM/GBSA's predicted binding energy (see methods) and is represented by its mean +standard deviation. HAC: heavy atom count, *i.e.* non-hydrogen atoms in the ligand. HAC can be used to normalize MM/GBSA calculations accounting for difference in the molecular sizes. Cumulative distributions of the two hits were compared using Kruskal-Wallis statistical test, where * represents P-value <0.05.

Figure S8 - 1H NMR spectra of final compound 1a (300 MHz, DMSO-d_6, δ = ppm).

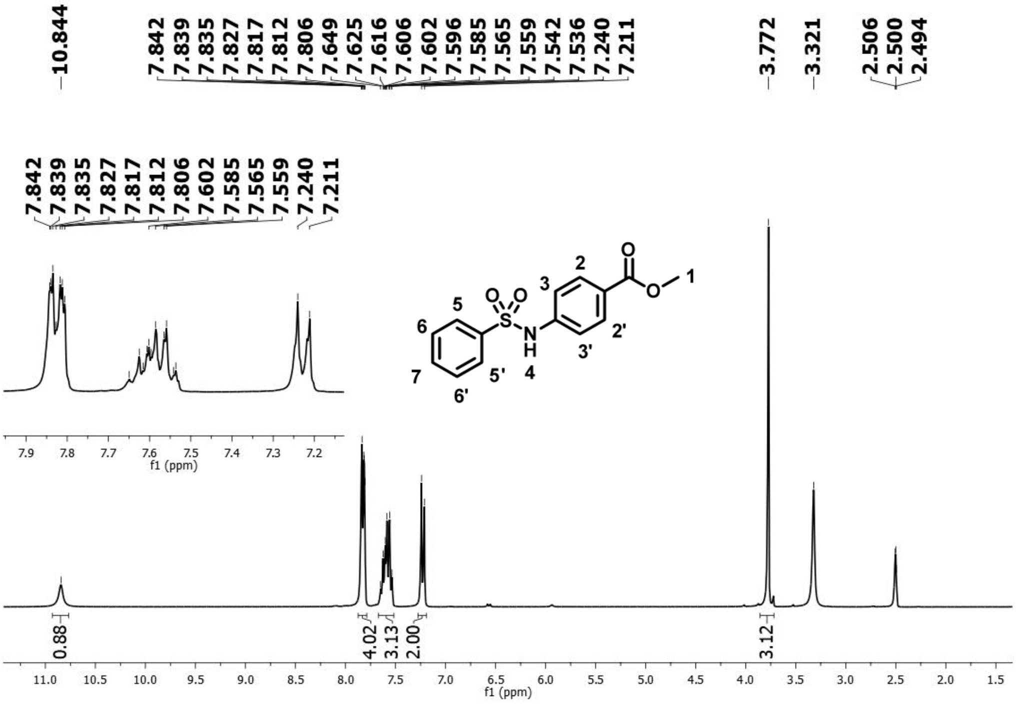

Figure S9 - 13C NMR spectra of the final compound 1a (300 MHz, DMSO-d_6, δ = ppm).

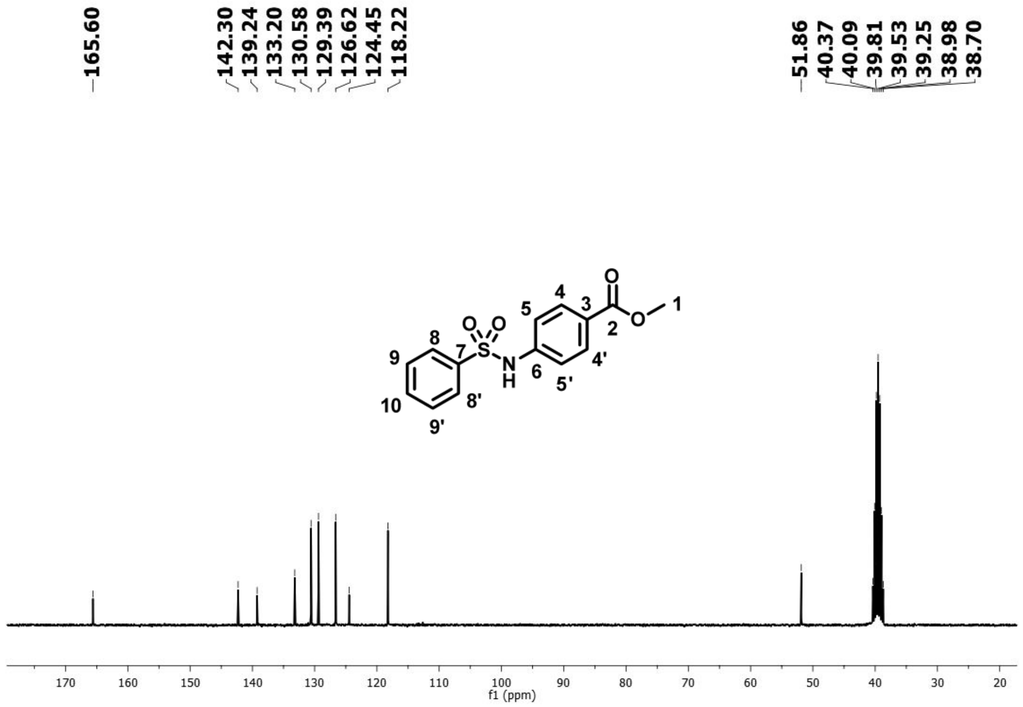

Figure S10 - 1H NMR spectra of final compound 1b (300 MHz, DMSO-d_6, δ = ppm).

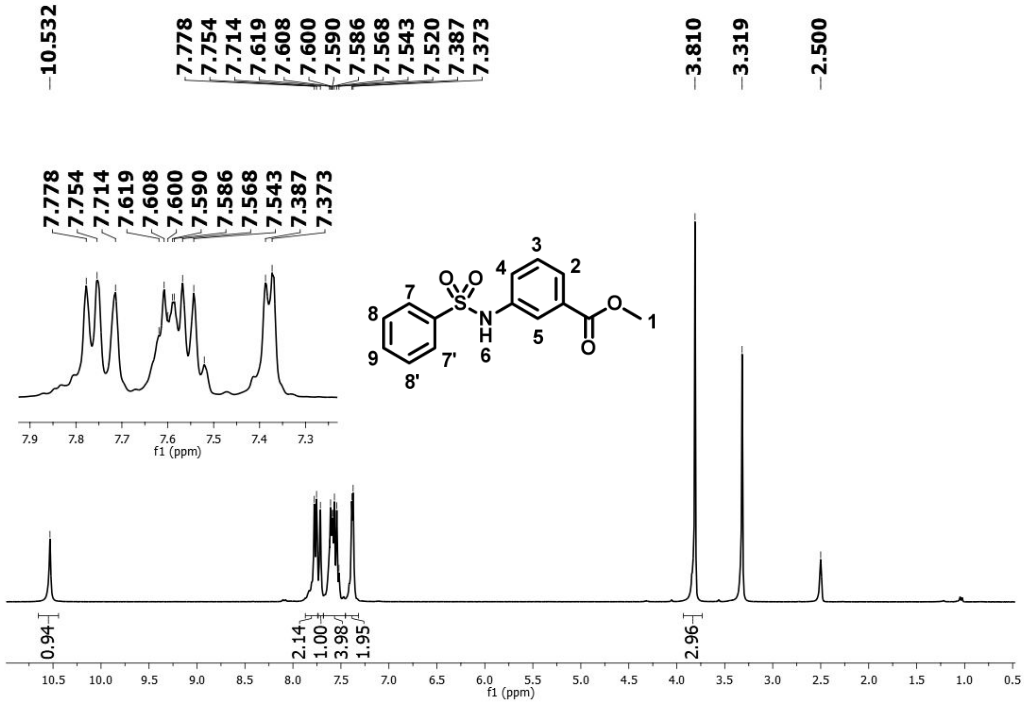

Figure S11 - 13C NMR spectra of the final compound 1b (300 MHz, DMSO-d$_6$, δ = ppm).

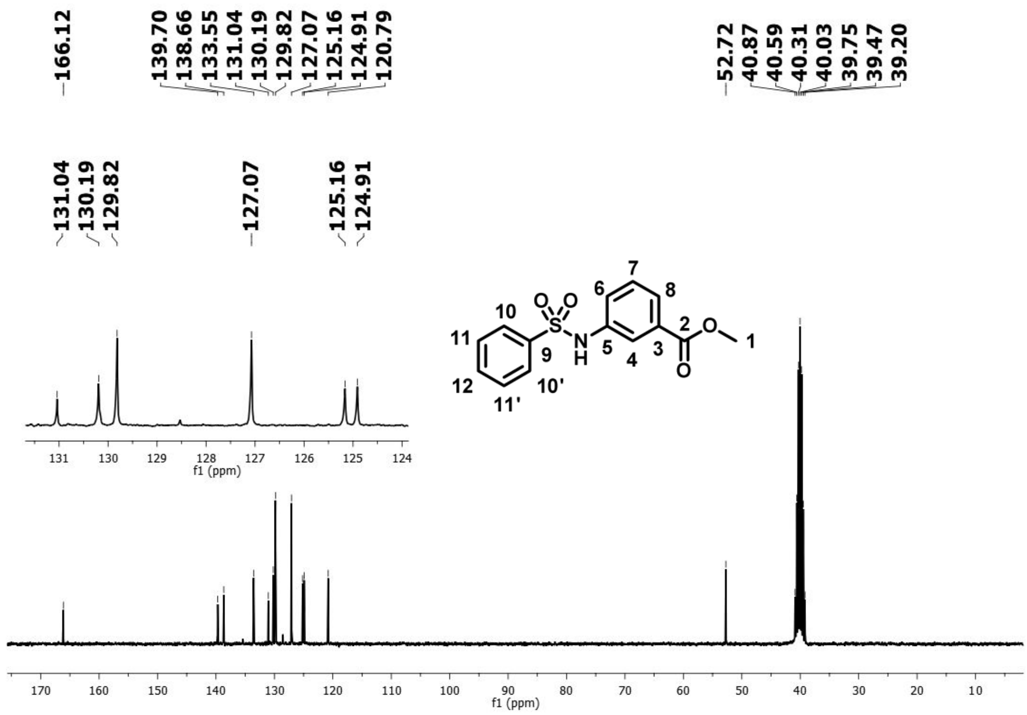

Figure S12 - 1H NMR spectra of final compound 2a (300 MHz, DMSO-d_6, δ = ppm).

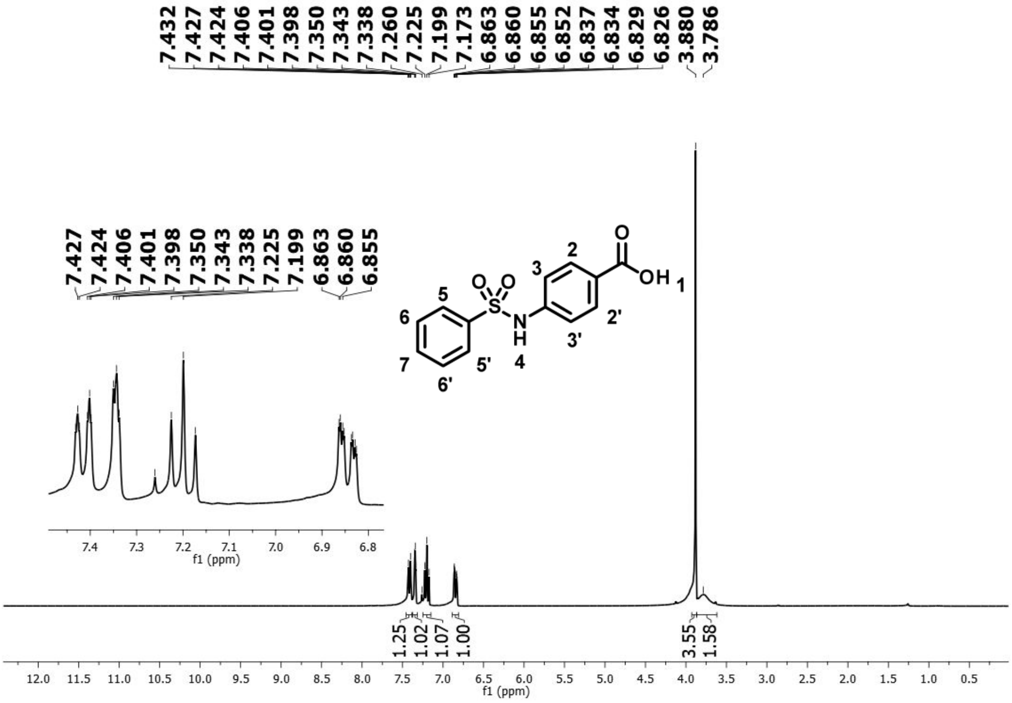

Figure S13 - 13C NMR spectra of the final compound 2a (300 MHz, DMSO-d$_6$, δ = ppm).

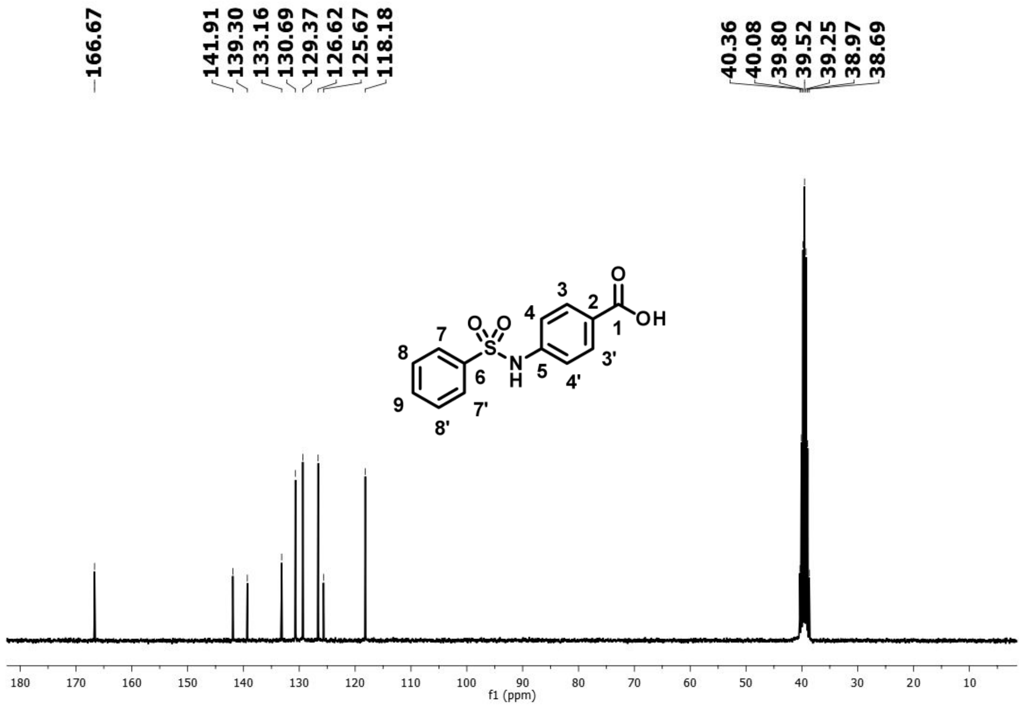

Figure S14 - 1H NMR spectra of final compound 2b (300 MHz, DMSO-d_6, δ = ppm).

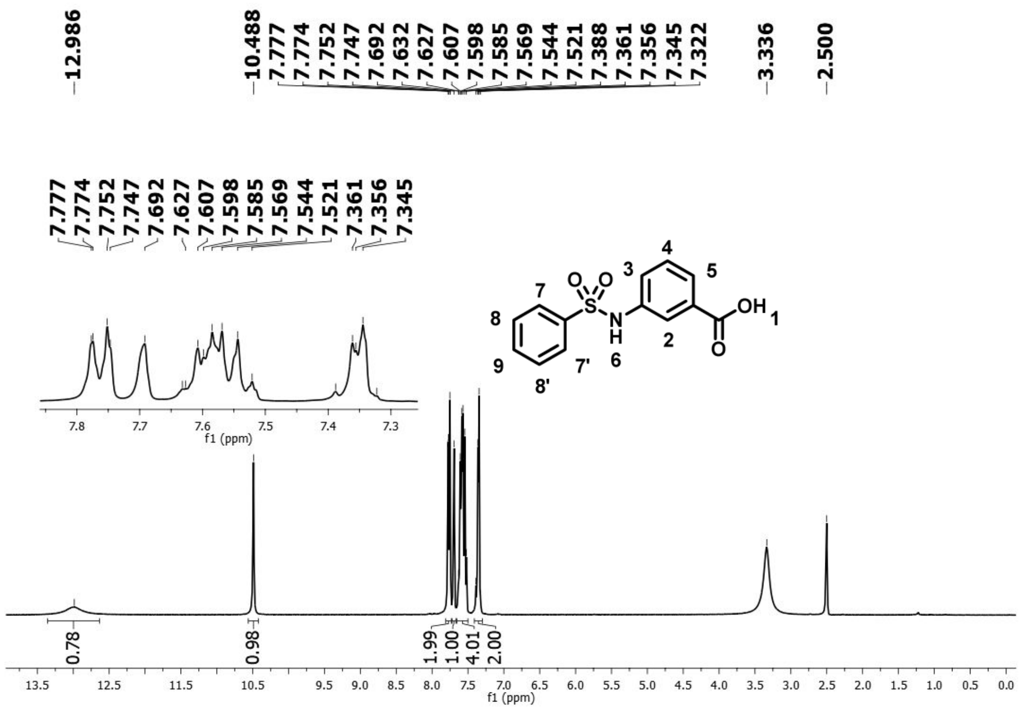

Figure S15 - 13C NMR spectra of final compound 2b (300 MHz, DMSO-d_6, δ = ppm).

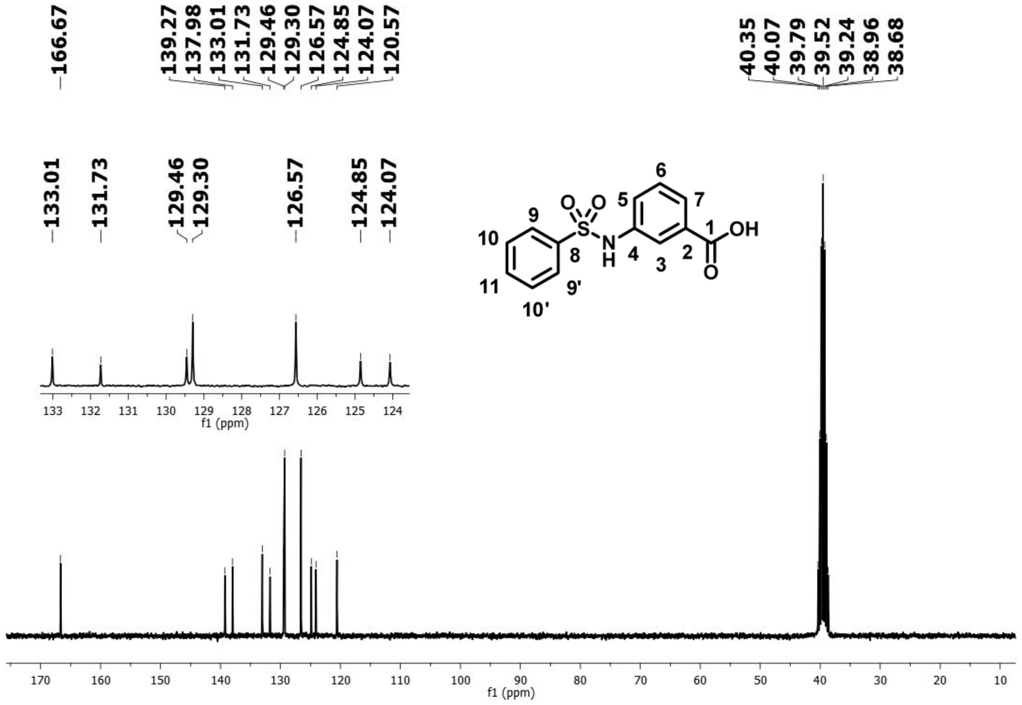

Figure S16 - 1H NMR spectra of final compound 3a (300 MHz, DMSO-d_6, δ = ppm).

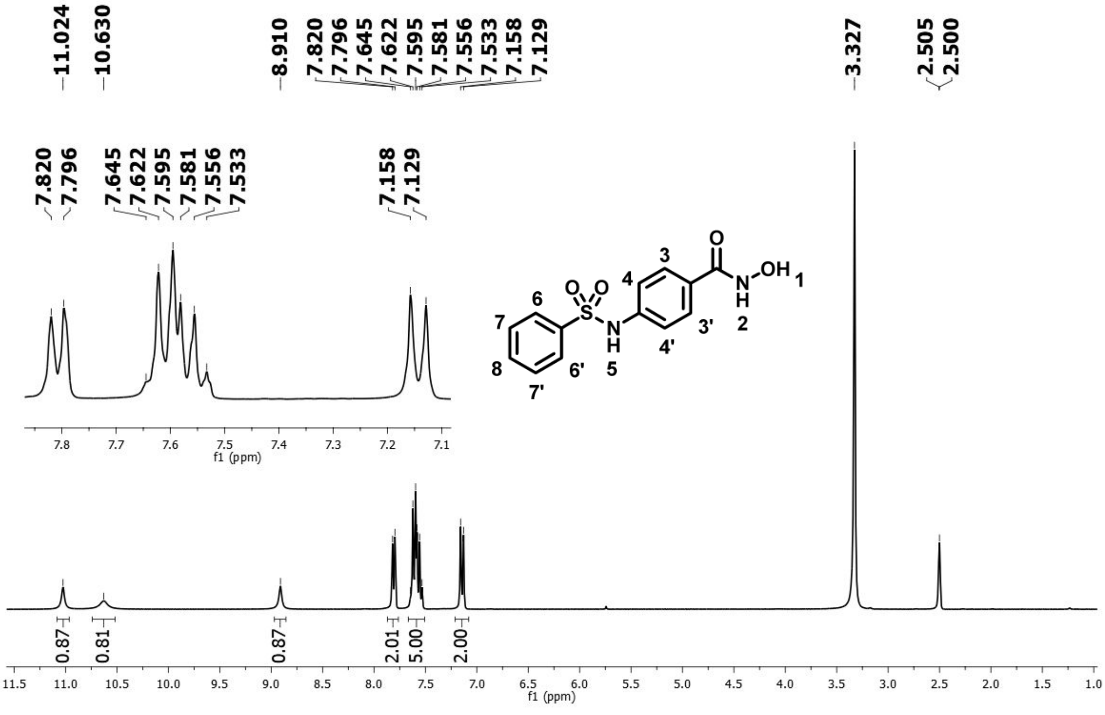

Figure S17. 13C NMR spectra of final compound 3a (300 MHz, DMSO-d_6, δ = ppm).

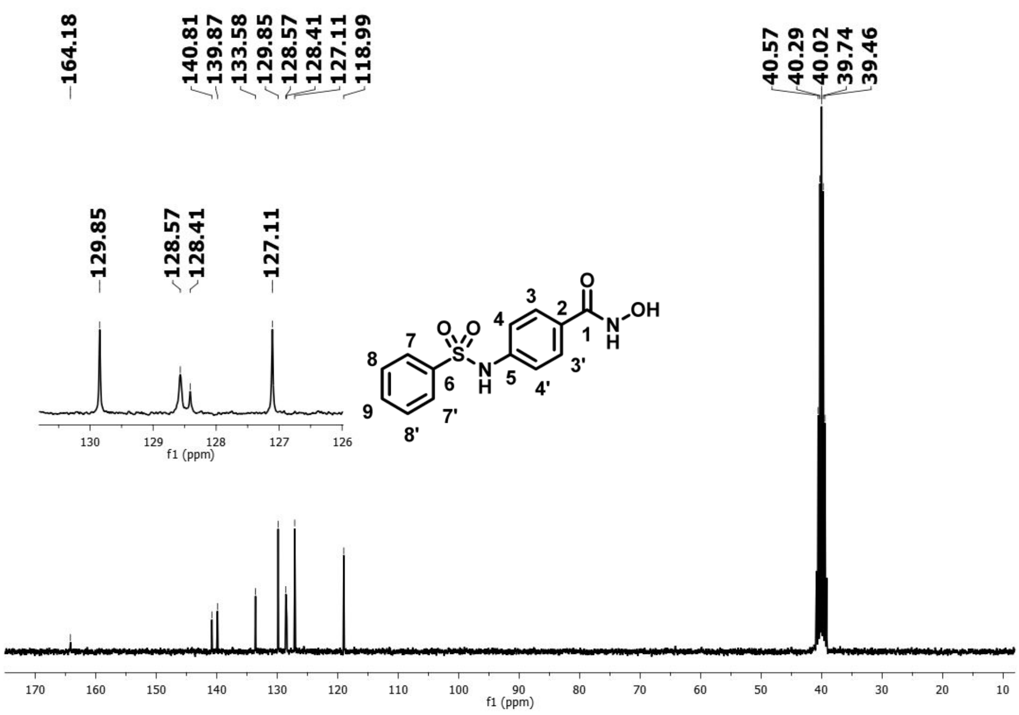

Figure S18 - 1H NMR spectra of final compound 3b (300 MHz, DMSO-d_6, δ = ppm).

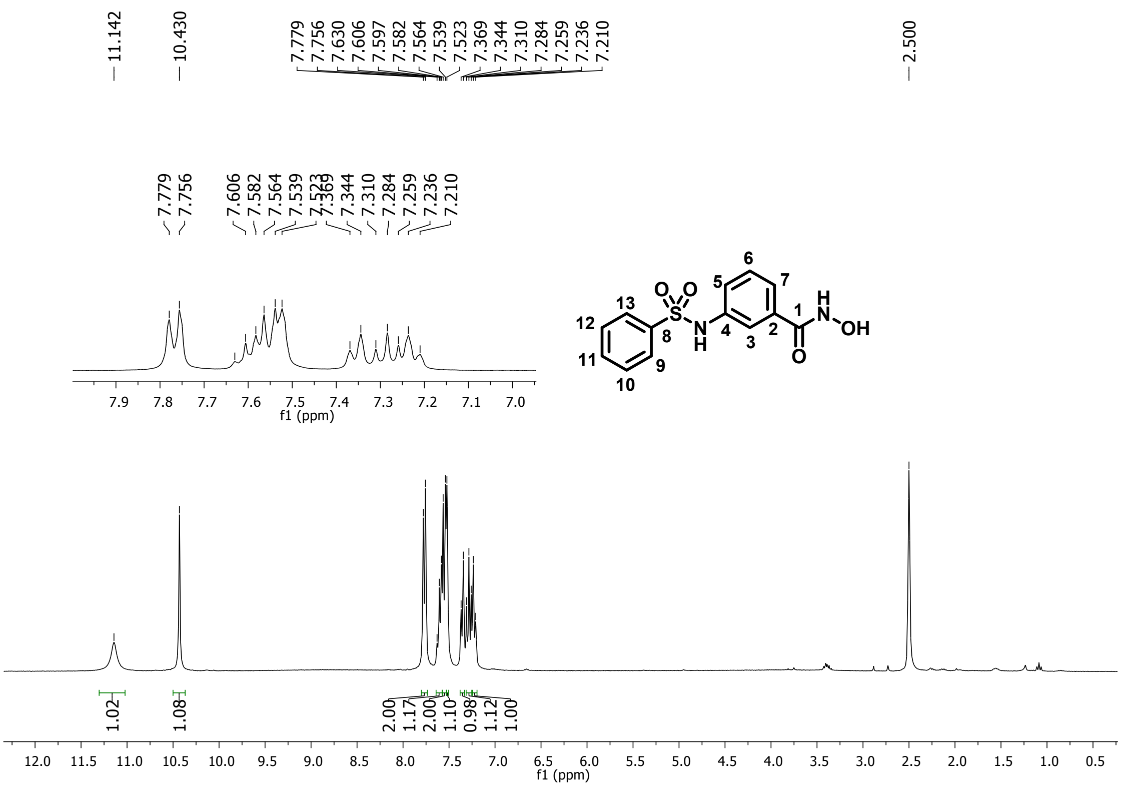

Figure S19 - 13C NMR spectra of final compound 3b (300 MHz, DMSO-d_6, δ = ppm).

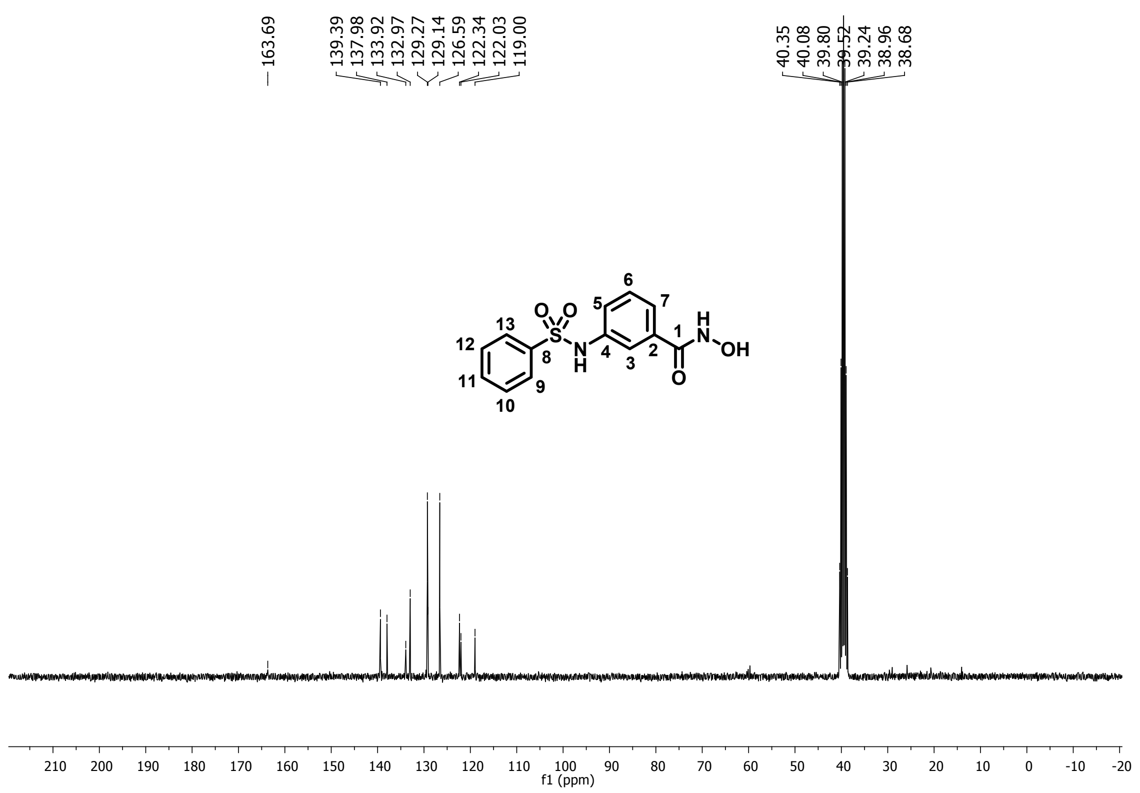

Figure S20 - 1H NMR spectra of final compound 4a (300 MHz, DMSO-d_6, δ = ppm).

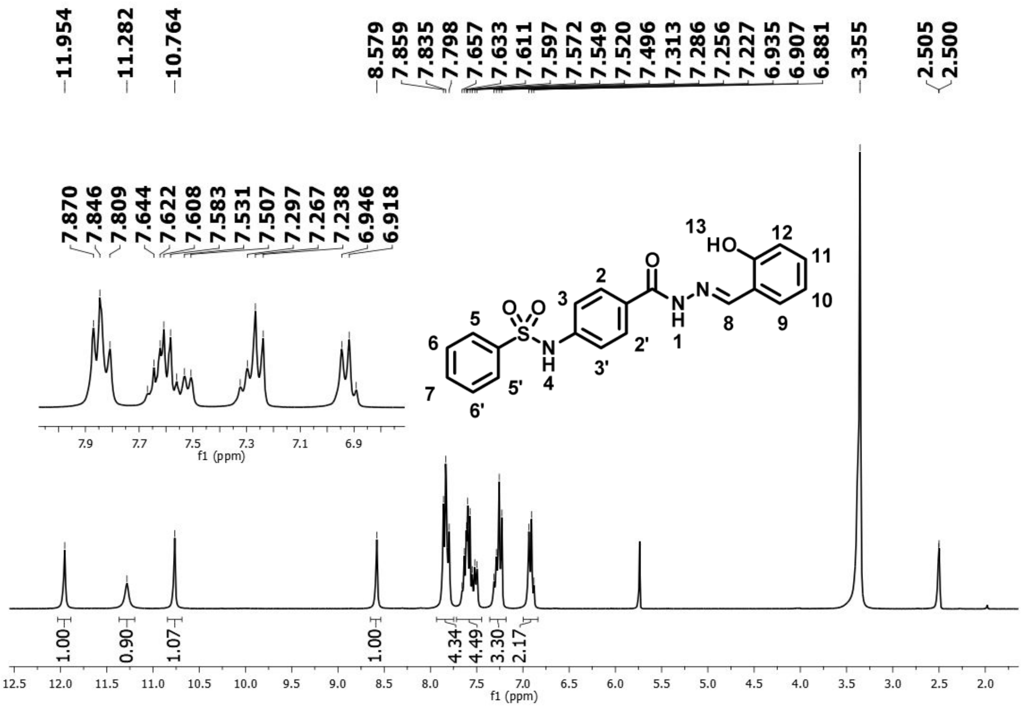

Figure S21 - 13C NMR spectra of final compound 4a (300 MHz, DMSO-d_6, δ = ppm).

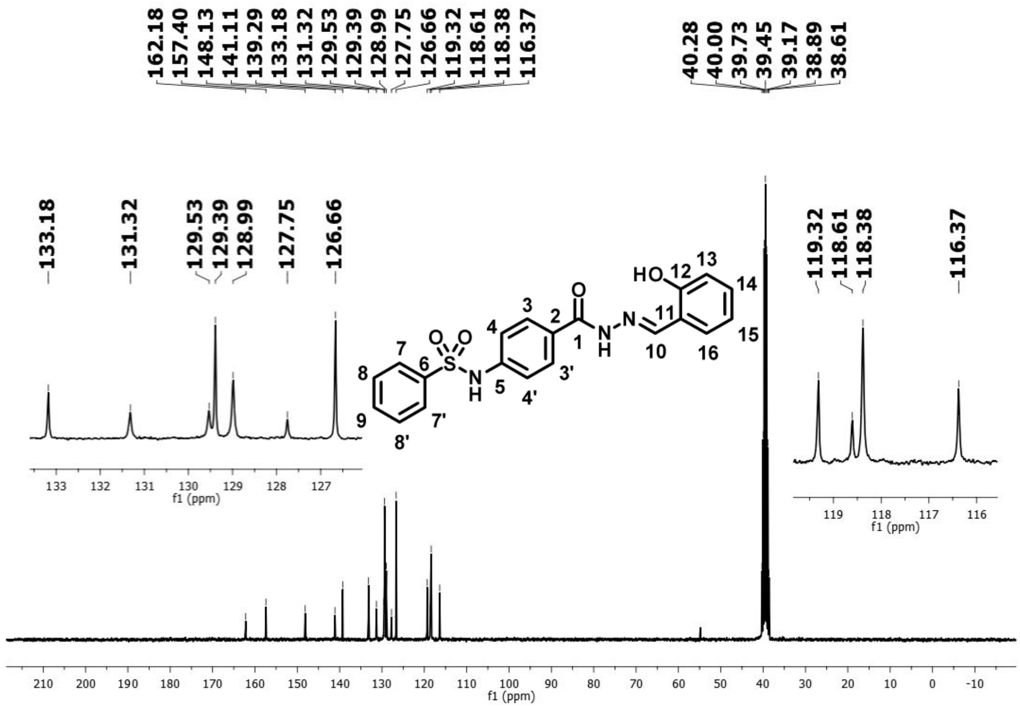

Figure S22 - 1H NMR spectra of final compound 4b (300 MHz, DMSO-d_6, δ = ppm).

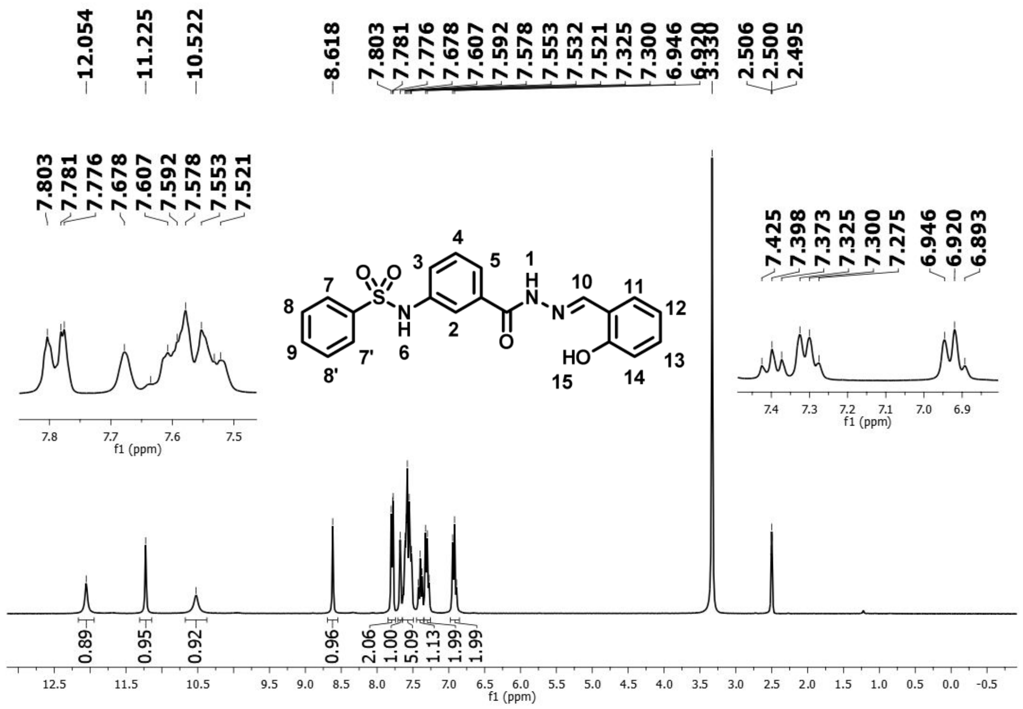

Figure S23 - 13C NMR spectra of final compound 4b (300 MHz, DMSO-d_6, δ = ppm).

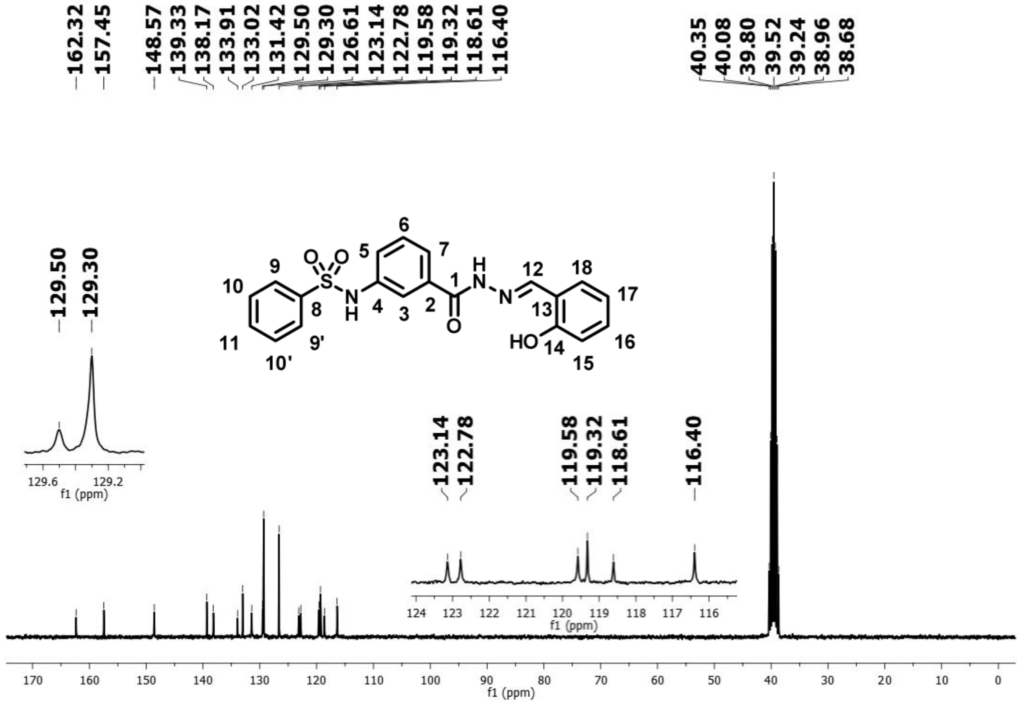

Figure S24 - 1H NMR spectra of final compound 5a (300 MHz, DMSO-d_6, δ = ppm).

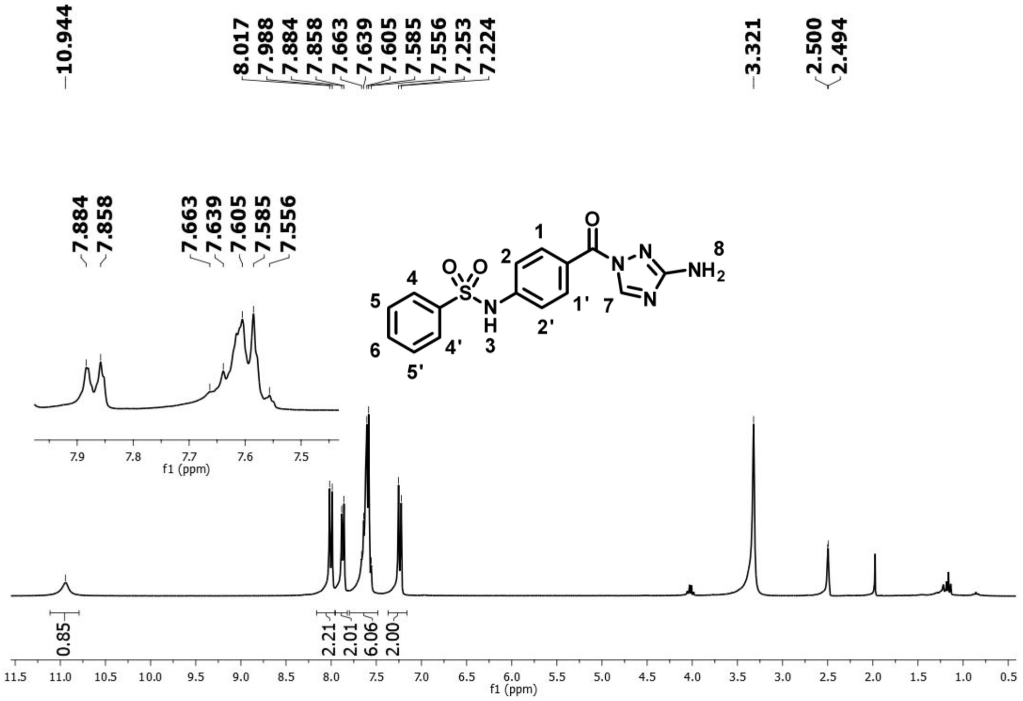

Figure S25 - 13C NMR spectra of final compound 5a (300 MHz, DMSO-d_6, δ = ppm).

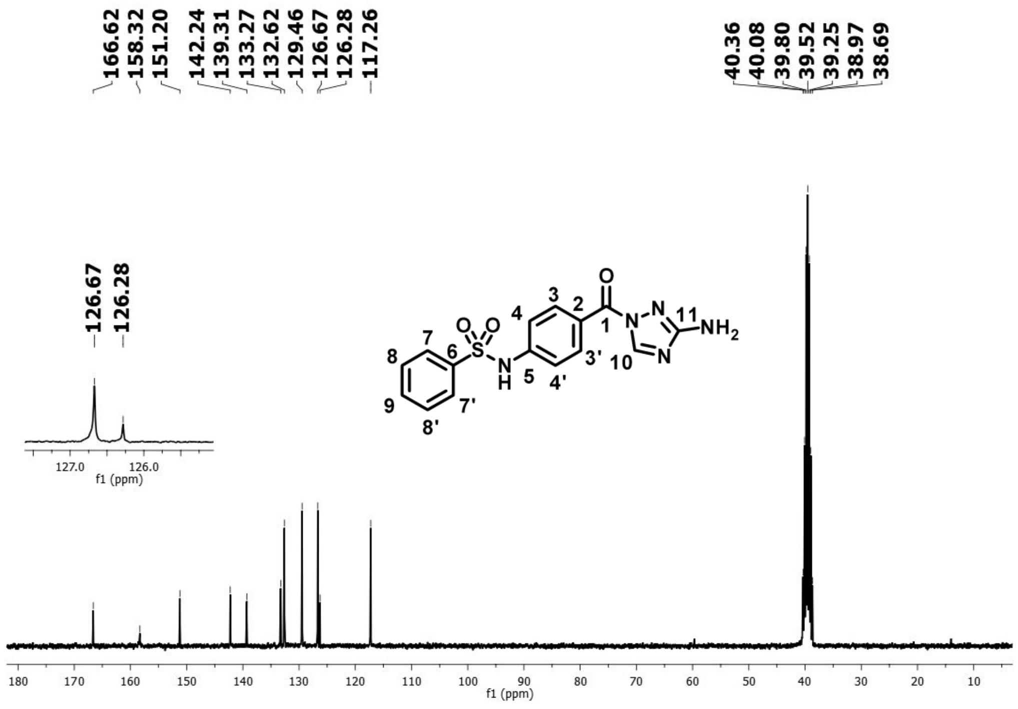

Figure S26 - Hetcor 13C / 1H NMR spectra of final compound 5a (75 /300 MHz, DMSO-d$_6$, δ = ppm).

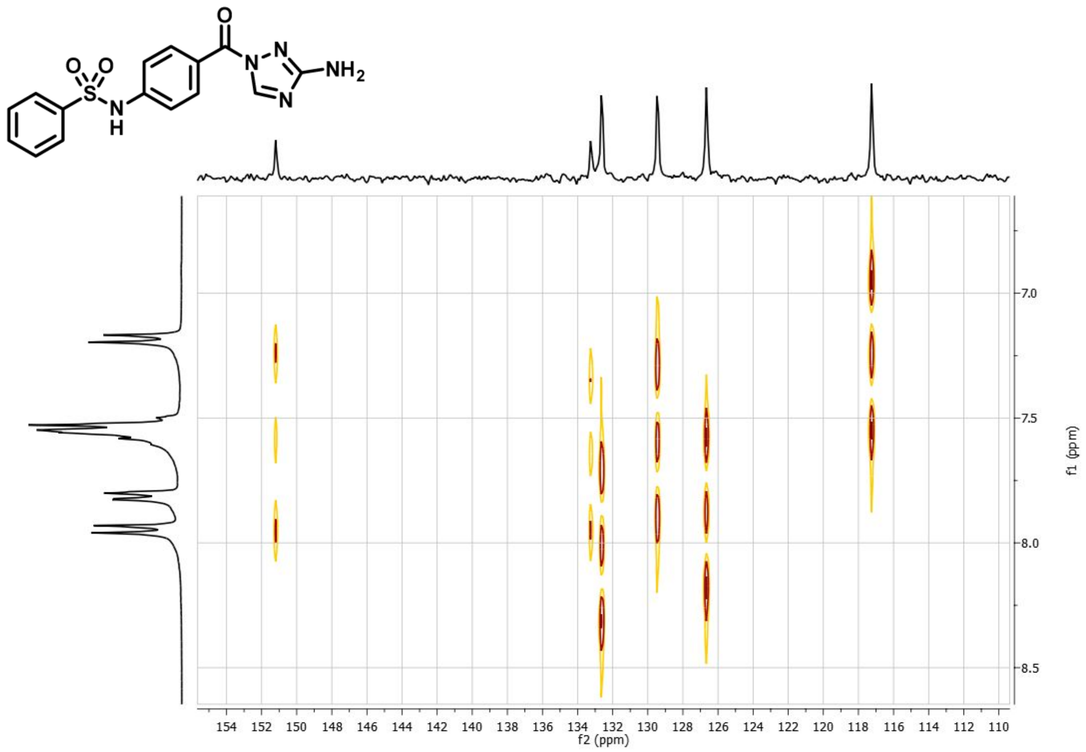

Figure S27 - HMBC 1H / 13C NMR spectra of final compound 5a (300 / 75 MHz, DMSO-d_6, δ = ppm).

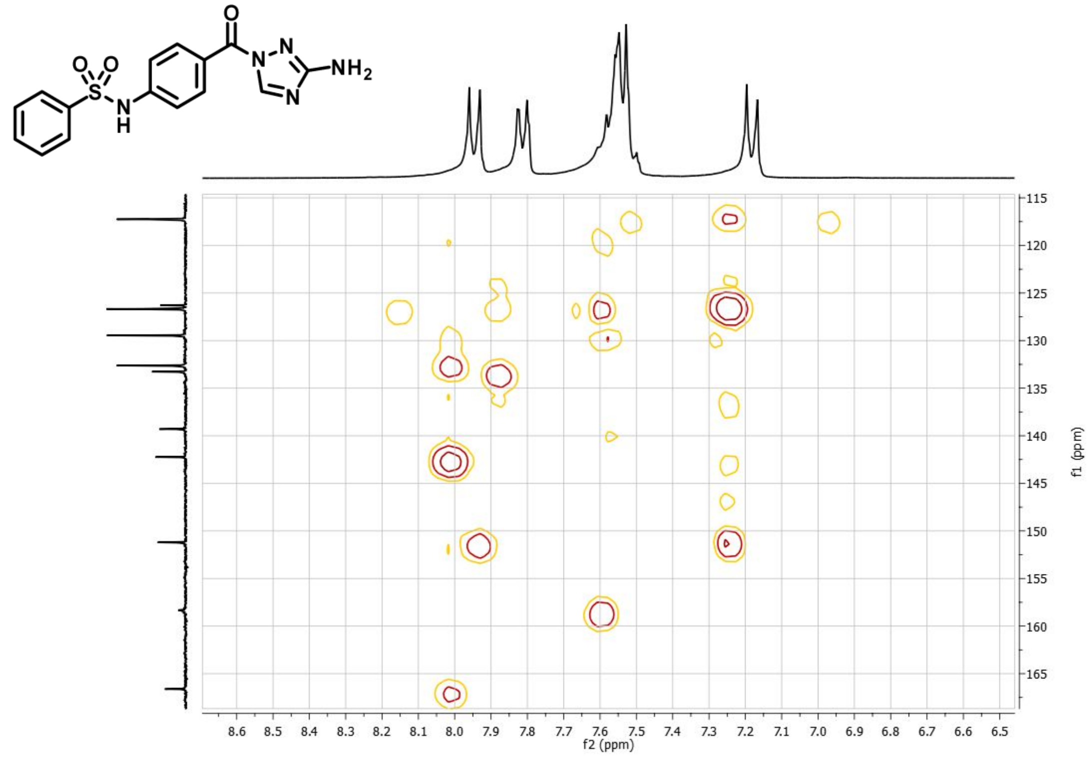

Figure S28 - 1H NMR spectra of final compound 5b (300 MHz, DMSO-d_6, δ = ppm).

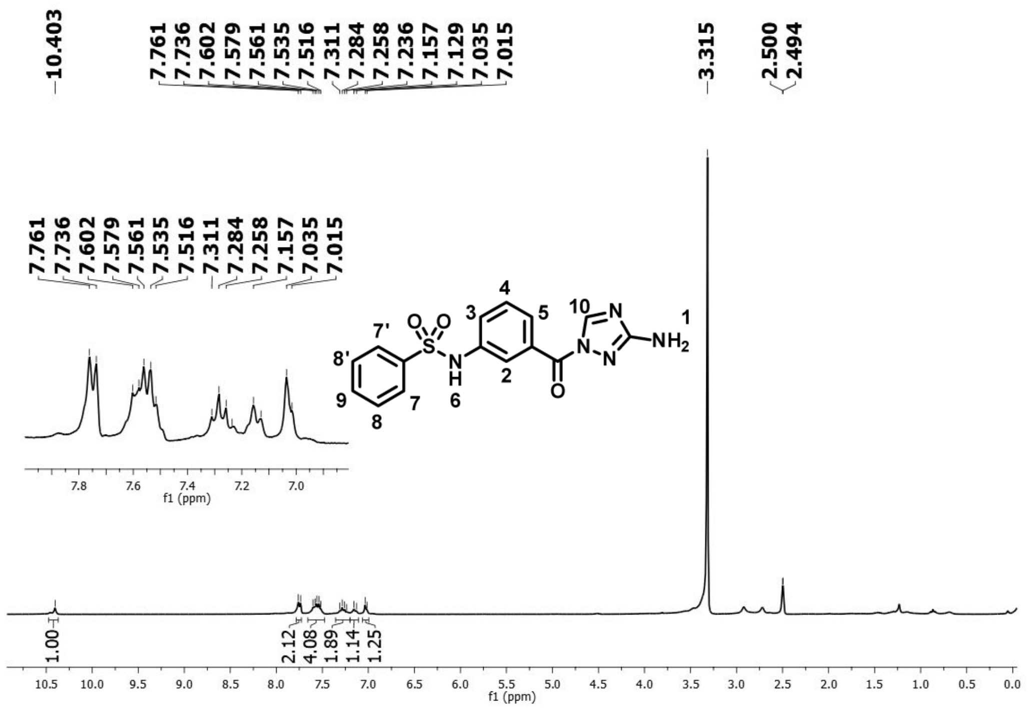

Figure S29 - 13C NMR spectra of final compound 5b (300 MHz, DMSO-d_6, δ = ppm).

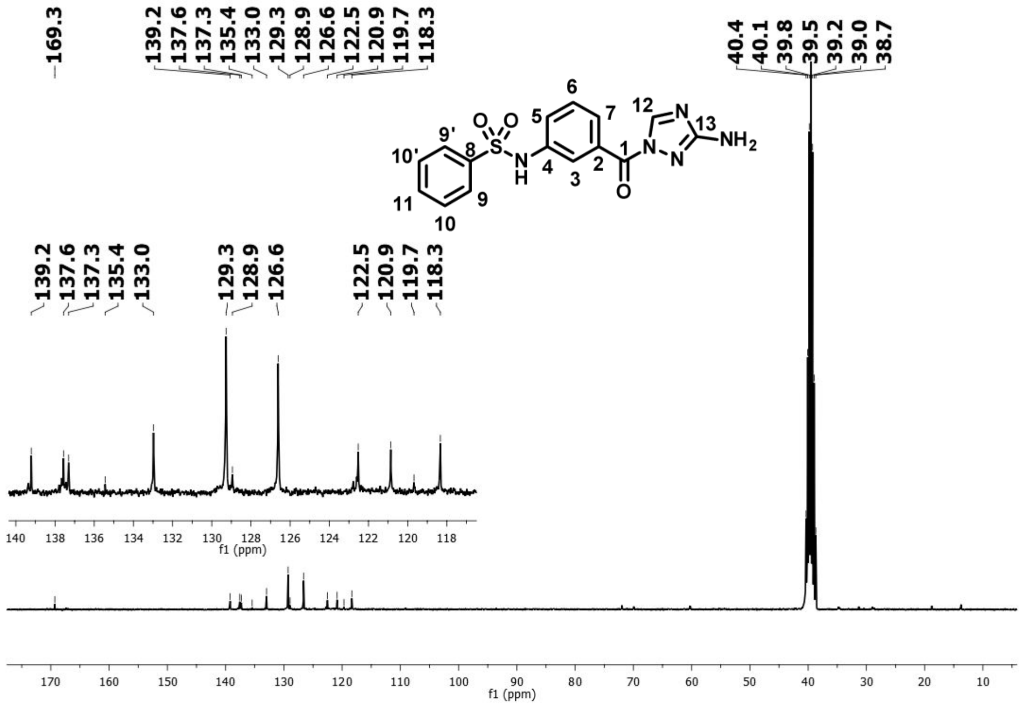

Figure S30 - 1H NMR spectra of final compound 6a (300 MHz, DMSO-d$_6$, δ = ppm).

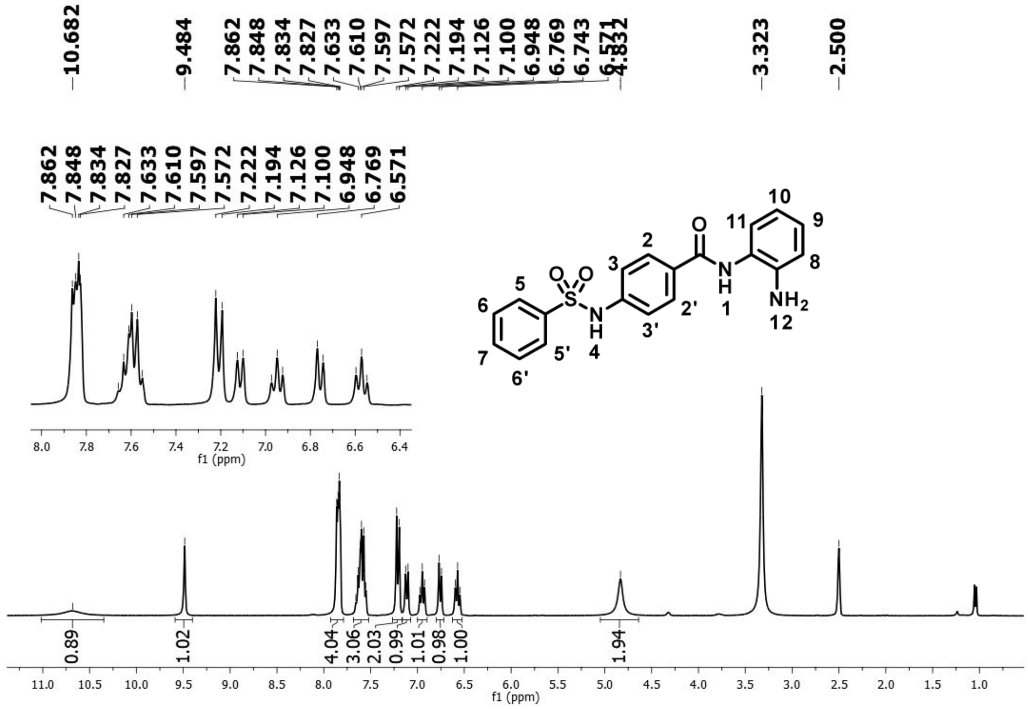

Figure S31 - 13C NMR spectra of final compound 6a (300 MHz, DMSO-d_6, δ = ppm).

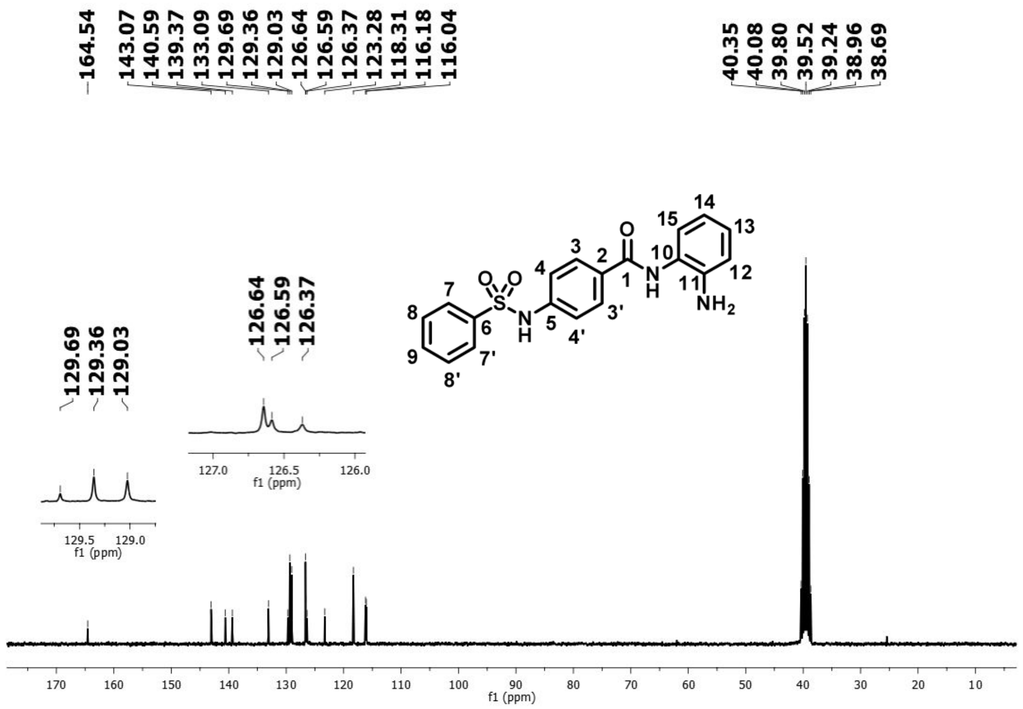

Figure S32 - 1H NMR spectra of final compound 6b (300 MHz, DMSO-d$_6$, δ = ppm).

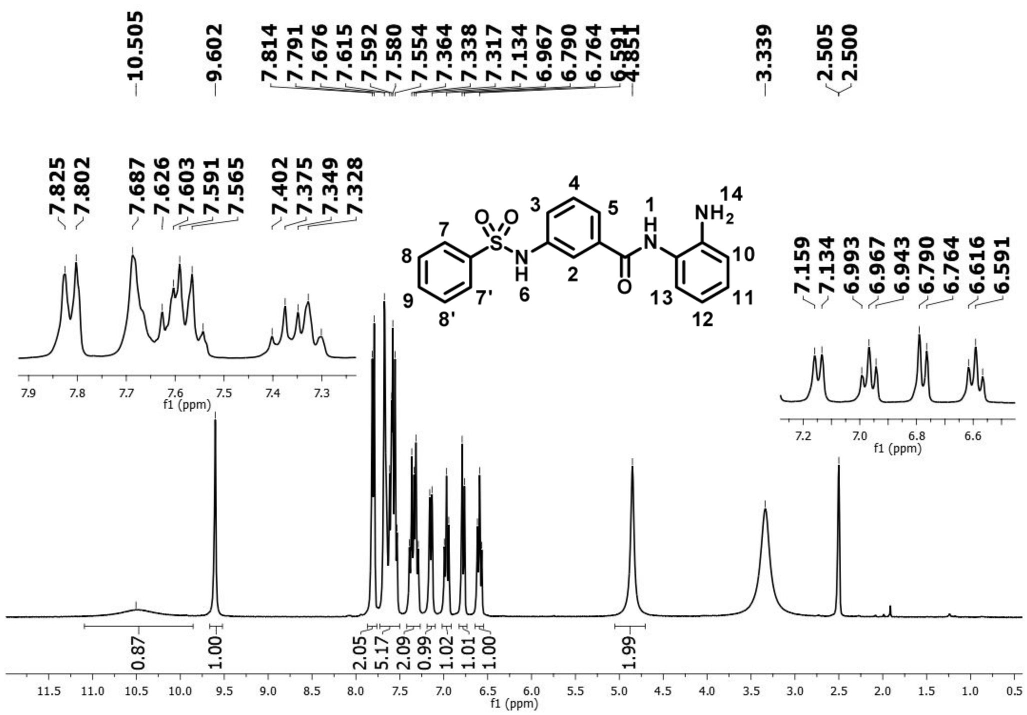

Figure S33 - 13C NMR spectra of final compound 6b (300 MHz, DMSO-d$_6$, δ = ppm).

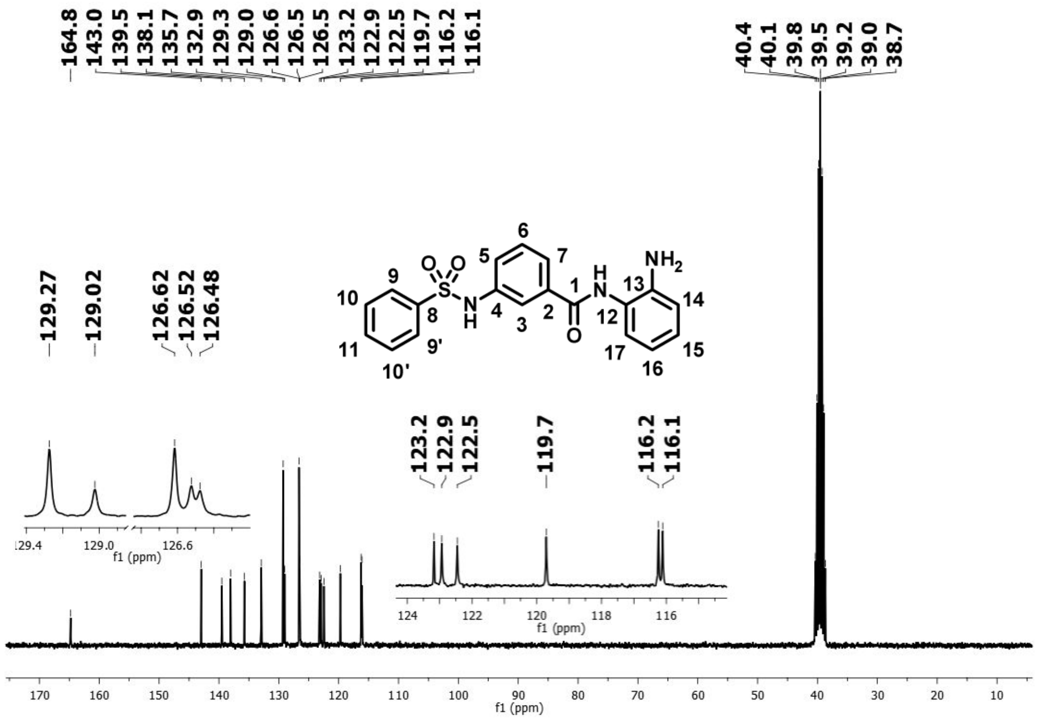

Figure S34 - HPLC spectra of the final compound 1a.

==== Shimadzu LCsolution Analysis Report ====

C:\LabSolutions\Data\Nuno\30.09.2019\P-BMEc.lcd

Acquired by	: Admin
Sample Name	: P-BMEc
Sample ID	:
Vail #	: 16
Injection Volume	: 5 uL
Data File Name	: P-BMEc.lcd
Method File Name	: ACN+0,1TFA-H2O+0,1TFA.lcm
Batch File Name	: 30.09.2019.lcb
Report File Name	: Default.lcr
Data Acquired	: 30/9/2019 21:52:07
Data Processed	: 1/10/2019 22:06:53

<Chromatogram>

C:\LabSolutions\Data\Nuno\30.09.2019\P-BMEc.lcd

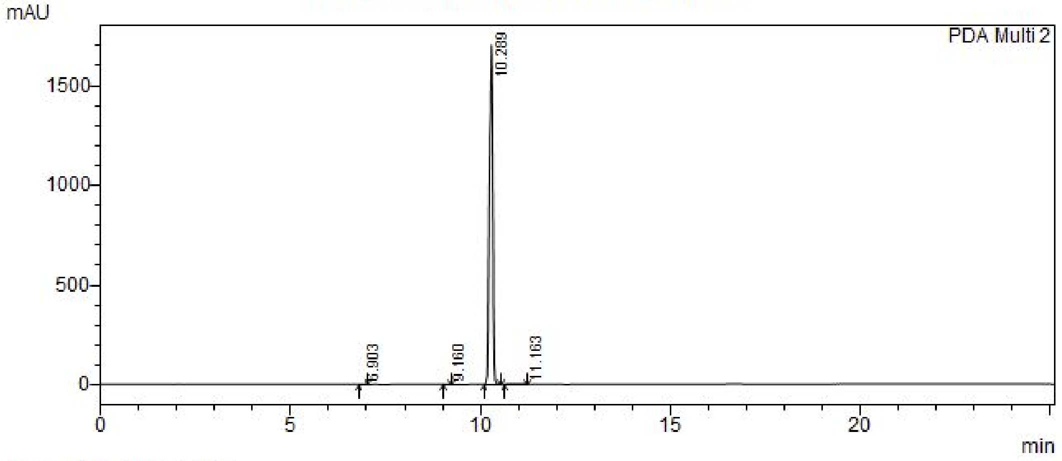

1 PDA Multi 2/261nm 4nm

PeakTable

PDA Ch2 261nm 4nm

Peak#	Name	Ret. Time	Area	Area %
1	RT6.903	6.903	3438	0.032
2	RT9.160	9.160	4454	0.041
3	RT10.289	10.289	10740102	99.614
4	RT11.163	11.163	33673	0.312
Total			10781667	100.000

Purity

Peak# : 3
Retention Time : 10.289
Compound Name : RT10.289

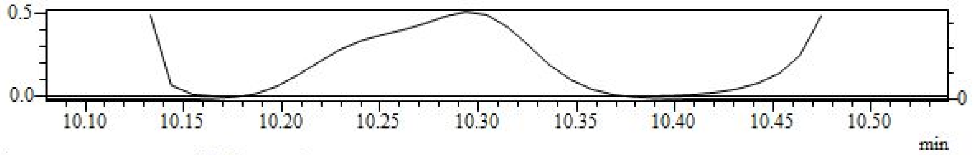

Impurity :Not Detected
Peak purity index : 1.000000
Single point threshold : 0.999978
Minimum peak purity index : 22

Figure S35 - HPLC spectra of the final compound 1b.

==== Shimadzu LCsolution Analysis Report ====

C:\LabSolutions\Data\Nuno\30.09.2019\M-BMEc.lcd

Acquired by	: Admin
Sample Name	: M-BMEc
Sample ID	:
Vail #	: 10
Injection Volume	: 5 uL
Data File Name	: M-BMEc.lcd
Method File Name	: ACN+0,1TFA-H2O+0,1TFA.lcm
Batch File Name	: 30.09.2019.lcb
Report File Name	: Default.lcr
Data Acquired	: 30/9/2019 19:13:53
Data Processed	: 1/10/2019 22:42:46

<Chromatogram>

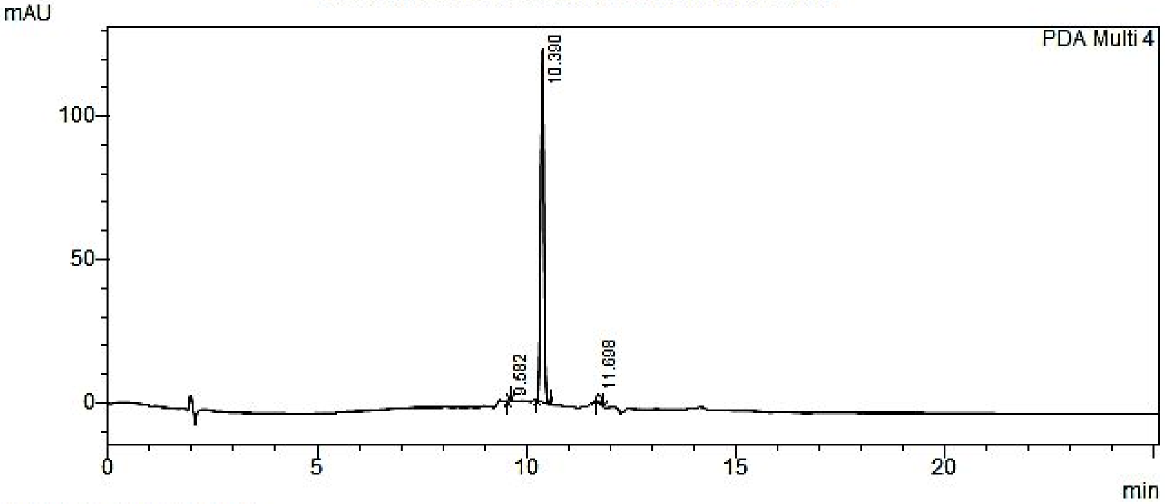

1 PDA Multi 4/290nm4nm

PeakTable

PDA Ch4 290nm 4nm

Peak#	Name	Ret. Time	Area	Area %
1	RT9.582	9.582	416	0.057
2	RT10.390	10.390	715509	97.369
3	RT11.698	11.698	18915	2.574
Total			734841	100.000

Purity

Peak# : 2
Retention Time : 10.390
Compound Name : RT10.390

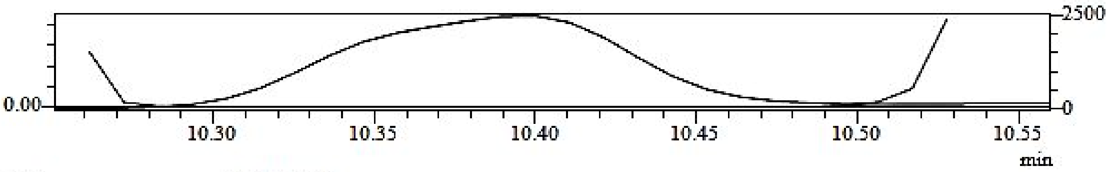

Impurity :Not Detected
Peak purity index : 1.000000
Single point threshold : 0.999977
Minimum peak purity index : 22

Figure S36 - HPLC spectra of the final compound 2a.

==== Shimadzu LCsolution Analysis Report ====

C:\LabSolutions\Data\Nuno\p-BAc.lcd

Acquired by	: Admin
Sample Name	: p-BAc
Sample ID	: p-BAc
Vail #	: 11
Injection Volume	: 5 uL
Data File Name	: p-BAc.lcd
Method File Name	: ACN+0,1TFA-H2O+0,1TFA.lcm
Batch File Name	: BatchThais03-12-17.lcb
Report File Name	: Report model LAPESSB.lcr
Data Acquired	: 10/9/2019 13:36:18
Data Processed	: 1/10/2019 18:09:09

<Chromatogram>

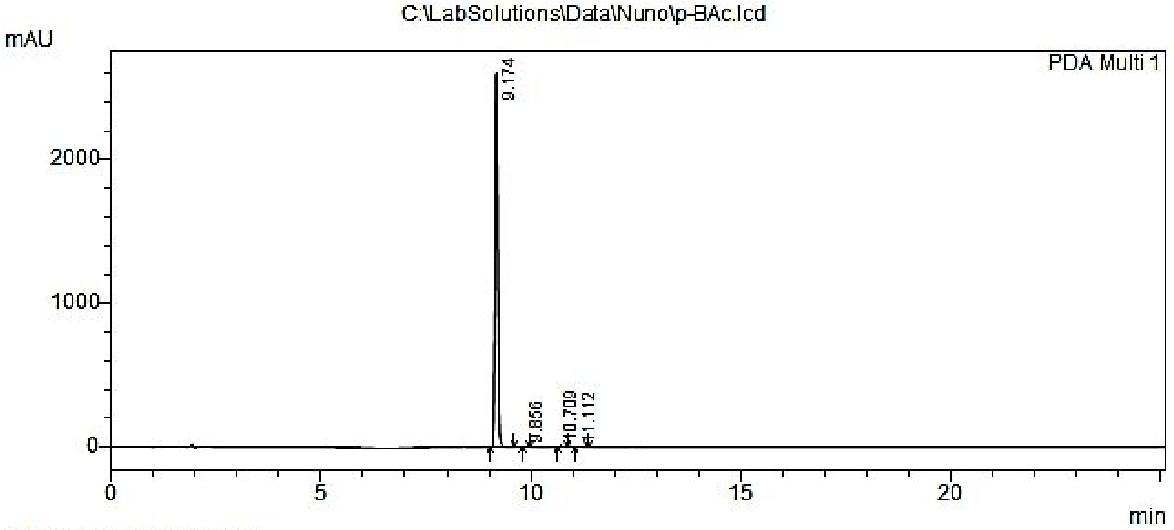

1 PDA Multi 1/239nm4nm

PeakTable

PDA Ch1 239nm 4nm

Peak#	Name	Ret. Time	Area	Area %
1	RT9.174	9.174	11307061	99.188
2	RT9.856	9.856	17713	0.155
3	RT10.709	10.709	52038	0.456
4	RT11.112	11.112	22854	0.200
Total			11399665	100.000

Purity

Peak# : 1
Retention Time : 9.174
Compound Name : RT9.174

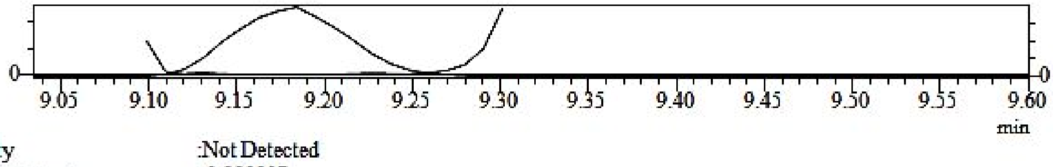

Impurity : Not Detected
Peak purity index : 0.999907
Single point threshold : 0.998486
Minimum peak purity index : 1420

Figure S37 - HPLC spectra of the final compound 2b.

==== Shimadzu LCsolution Analysis Report ====

C:\LabSolutions\Data\Nuno\30.09.2019\M-BAc.lcd

Acquired by	: Admin
Sample Name	: M-BAc
Sample ID	:
Vail #	: 14
Injection Volume	: 5 uL
Data File Name	: M-BAc.lcd
Method File Name	: ACN+0,1TFA-H2O+0,1TFA.lcm
Batch File Name	: 30.09.2019.lcb
Report File Name	: Default.lcr
Data Acquired	: 30/9/2019 20:59:24
Data Processed	: 1/10/2019 22:32:39

<Chromatogram>

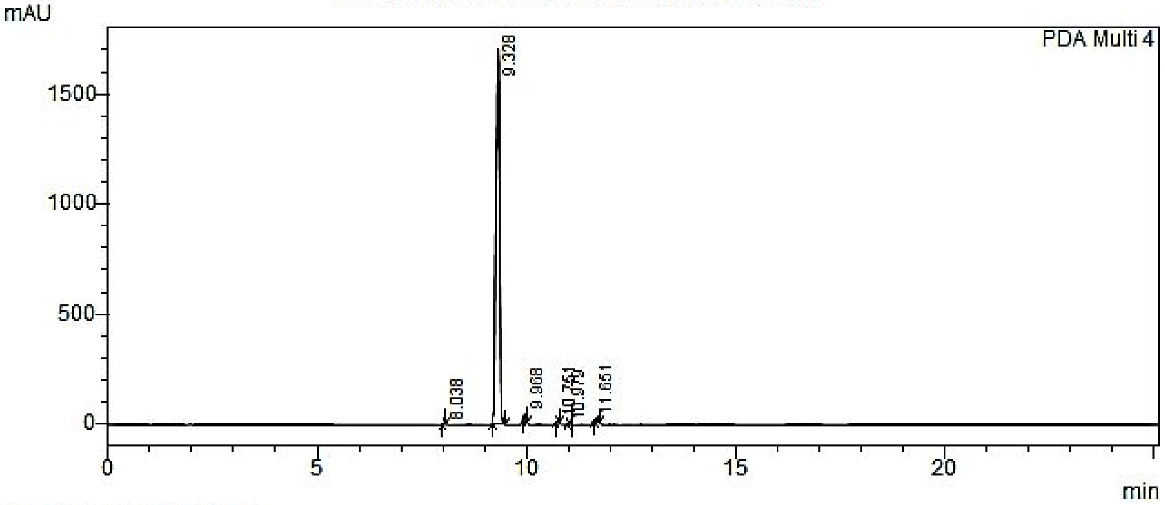

1 PDA Multi 4/240nm4nm

PeakTable

PDA Ch4 240nm 4nm

Peak#	Name	Ret. Time	Area	Area %
1	RT8.038	8.038	3163	0.030
2	RT9.328	9.328	10190677	97.532
3	RT9.968	9.968	134367	1.286
4	RT10.751	10.751	16376	0.157
5	RT10.979	10.979	154	0.001
6	RT11.651	11.651	103842	0.994
Total			10448580	100.000

Purity

Peak# : 2
Retention Time : 9.328
Compound Name : RT9.328

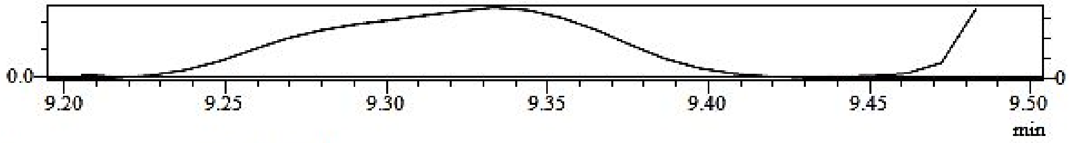

Impurity : Not Detected
Peak purity index : 0.999989
Single point threshold : 0.999989
Minimum peak purity index : 0

Figure S38 - HPLC spectra of the final compound 3a.

==== Shimadzu LCsolution Analysis Report ====

C:\LabSolutions\Data\Nuno\p-NT01c.lcd

Acquired by	: Admin
Sample Name	: p-NT01c
Sample ID	: p-NT01c
Vail #	: 12
Injection Volume	: 5 uL
Data File Name	: p-NT01c.lcd
Method File Name	: ACN+0,1TFA-H2O+0,1TFA.lcm
Batch File Name	: BatchThais03-12-17.lcb
Report File Name	: Report model LAPESSB.lcr
Data Acquired	: 10/9/2019 14:02:40
Data Processed	: 1/10/2019 17:18:07

<Chromatogram>

C:\LabSolutions\Data\Nuno\p-NT01c.lcd

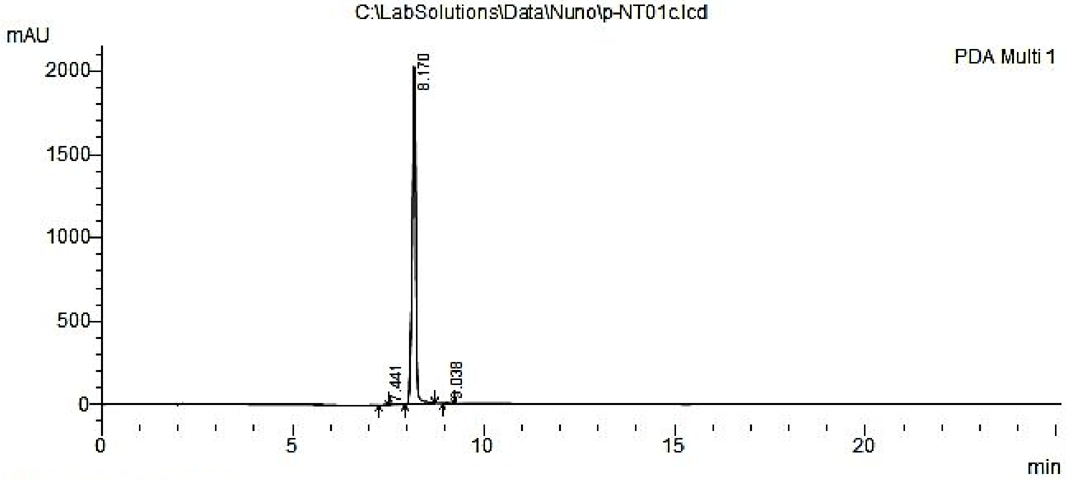

1 PDA Multi 1/256nm 4nm

PeakTable

PDA Ch1 256nm 4nm

Peak#	Name	Ret. Time	Area	Area %
1	RT7.441	7.441	5980	0.046
2	RT8.170	8.170	13113511	99.874
3	RT9.038	9.038	10535	0.080
Total			13130026	100.000

Purity

Peak# : 2
Retention Time : 8.170
Compound Name : RT8.170

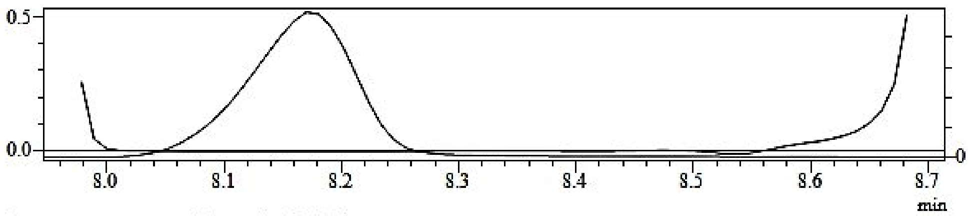

Impurity :Detected at 8.53 min
Peak purity index : 0.979129
Single point threshold : 0.989765
Minimum peak purity index : -10636

Figure S39 - HPLC spectra of the final compound 4a.

==== Shimadzu LCsolution Analysis Report ====

C:\LabSolutions\Data\Nuno\p-NT02c.lcd

Acquired by	: Admin
Sample Name	: p-NT02c
Sample ID	: p-NT02c
Vail #	: 13
Injection Volume	: 5 uL
Data File Name	: p-NT02c.lcd
Method File Name	: ACN+0,1TFA-H2O+0,1TFA.lcm
Batch File Name	: BatchThais03-12-17.lcb
Report File Name	: Report model LAPESSB.lcr
Data Acquired	: 10/9/2019 14:29:03
Data Processed	: 1/10/2019 21:15:25

<Chromatogram>

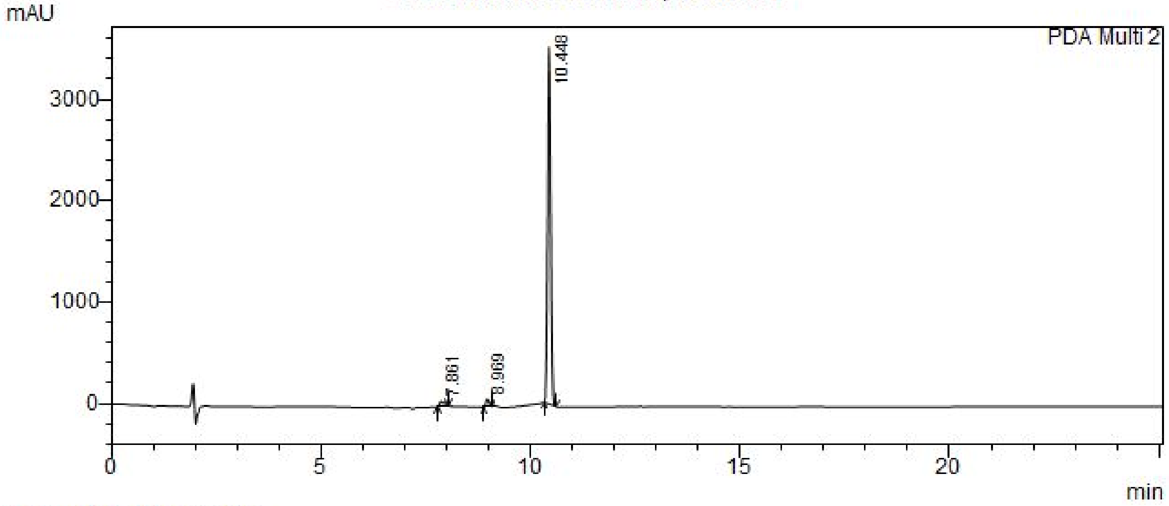

1 PDA Multi 2/215nm 4nm

PeakTable

PDA Ch2 215nm 4nm

Peak#	Name	Ret. Time	Area	Area %
1	RT7.861	7.861	424968	2.334
2	RT8.969	8.969	383713	2.107
3	RT10.448	10.448	17400036	95.559
Total			18208717	100.000

Purity

Peak# : 3
Retention Time : 10.448
Compound Name : RT10.448

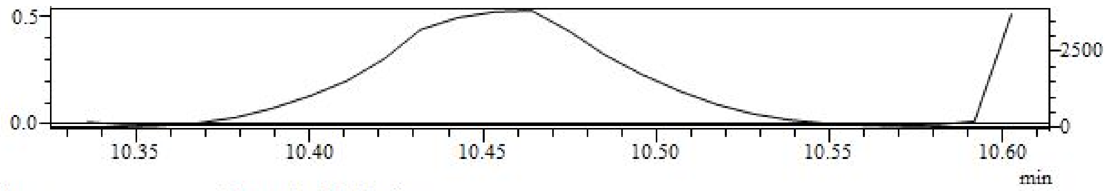

Impurity	:Detected at 10.46 min
Peak purity index	: 0.999639
Single point threshold	: 0.999990
Minimum peak purity index	: -351

Figure S40 - HPLC spectra of the final compound 4b.

==== Shimadzu LCsolution Analysis Report ====

C:\LabSolutions\Data\Nuno\m-NT02c.lcd

Acquired by : Admin
Sample Name : m-NT02c
Sample ID : m-NT02c
Vail # : 14
Injection Volume : 5 uL
Data File Name : m-NT02c.lcd
Method File Name : ACN+0,1TFA-H2O+0,1TFA.lcm
Batch File Name : BatchThais03-12-17.lcb
Report File Name : Report model LAPESSB.lcr
Data Acquired : 10/9/2019 14:55:24
Data Processed : 1/10/2019 15:15:23

<Chromatogram>

C:\LabSolutions\Data\Nuno\m-NT02c.lcd

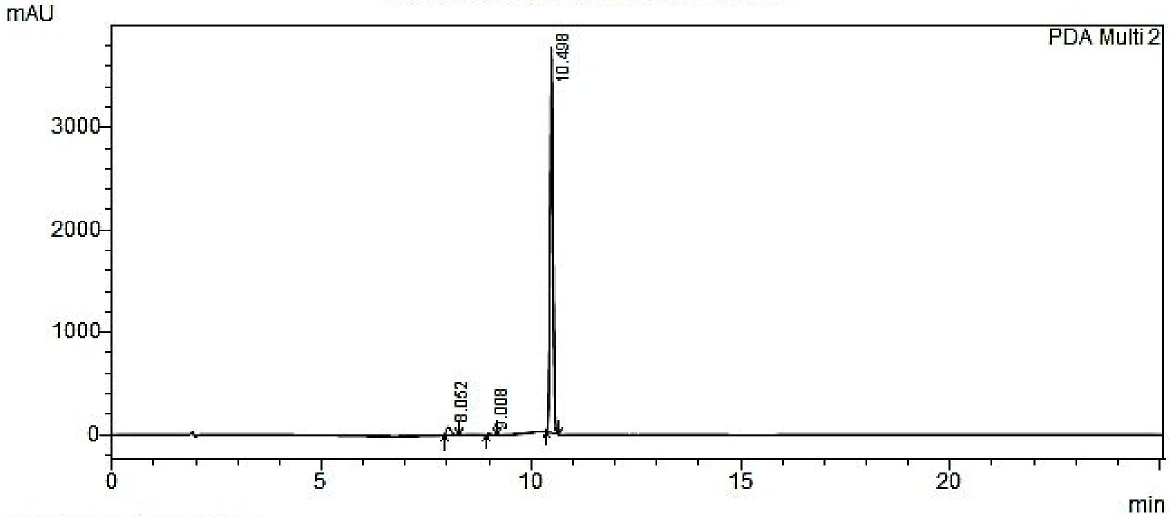

1 PDA Multi 2/235nm4nm

PeakTable

PDA Ch2 235nm 4nm

Peak#	Name	Ret. Time	Area	Area %
1	RT8.052	8.052	606724	3.162
2	RT9.008	9.008	90988	0.474
3	RT10.498	10.498	18488355	96.363
Total			19186068	100.000

Purity

Peak# : 3
Retention Time : 10.498
Compound Name : RT10.498

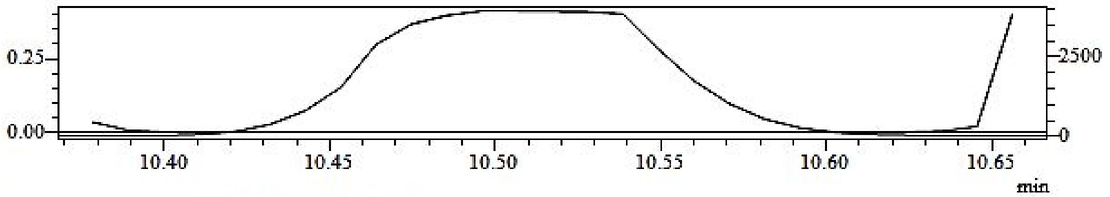

Impurity :Detected at 10.52 min
Peak purity index : 0.999745
Single point threshold : 0.999995
Minimum peak purity index : -249

Figure S41 - HPLC spectra of the final compound 5a.

==== Shimadzu LCsolution Analysis Report ====

C:\LabSolutions\Data\Nuno\p-NT03c1.lcd

Acquired by	: Admin
Sample Name	: p-NT03c
Sample ID	: Amostra
Vail #	: 1
Injection Volume	: 5 uL
Data File Name	: p-NT03c1.lcd
Method File Name	: ACN+0,1TFA-H2O+0,1TFA.lcm
Batch File Name	:
Report File Name	: relat-Denise.lcr
Data Acquired	: 10/9/2019 17:02:50
Data Processed	: 1/10/2019 21:39:09

<Chromatogram>

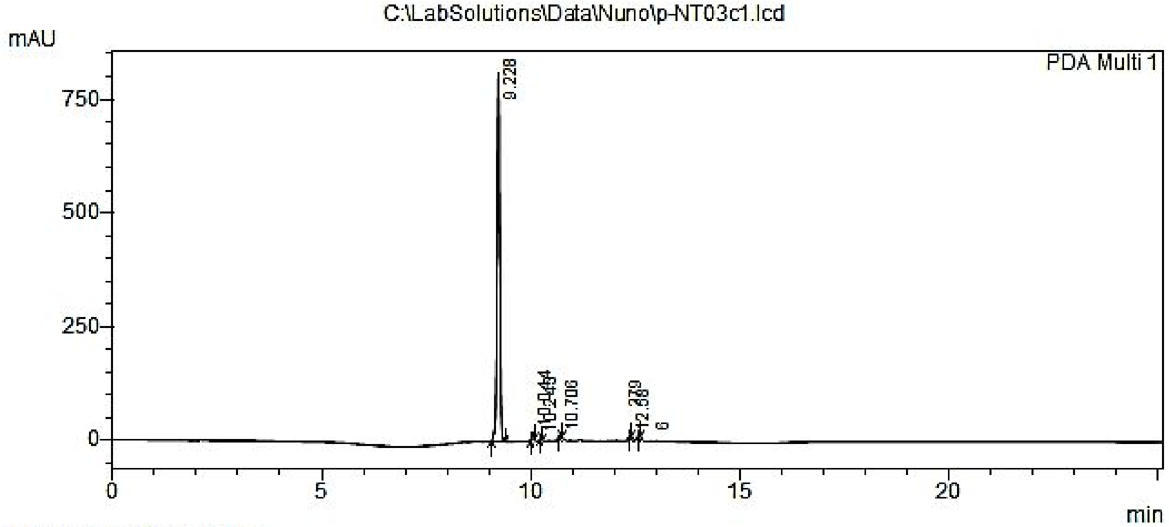

1 PDA Multi 1/240nm4nm

PeakTable

PDA Ch1 240nm 4nm

Peak#	Name	Ret. Time	Area	Area %
1	RT9.228	9.228	3672459	97.113
2	RT10.044	10.044	50201	1.327
3	RT10.243	10.243	26624	0.704
4	RT10.706	10.706	12289	0.325
5	RT12.379	12.379	9479	0.251
6	RT12.586	12.586	10595	0.280
Total			3781646	100.000

Purity

Peak#	:1
Retention Time	:9.228
Compound Name	:RT9.228

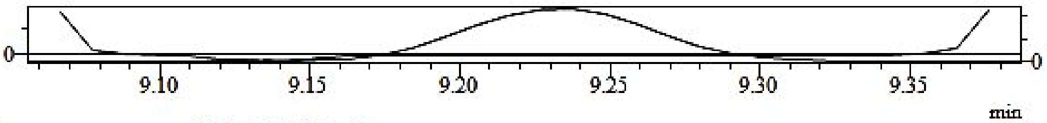

Impurity	:Detected at 9.14 min
Peak purity index	:0.955779
Single point threshold	:0.999710
Minimum peak purity index	: -43931

Figure S42 - HPLC spectra of the final compound 5b.

==== Shimadzu LCsolution Analysis Report ====

C:\LabSolutions\Data\Nuno\30.09.2019\M-NT03c.lcd

Acquired by	: Admin
Sample Name	: M-NT03c
Sample ID	:
Vail #	: 13
Injection Volume	: 5 uL
Data File Name	: M-NT03c.lcd
Method File Name	: ACN+0,1TFA-H2O+0,1TFA.lcm
Batch File Name	: 30.09.2019.lcb
Report File Name	: Default.lcr
Data Acquired	: 30/9/2019 20:33:03
Data Processed	: 1/10/2019 22:59:06

<Chromatogram>

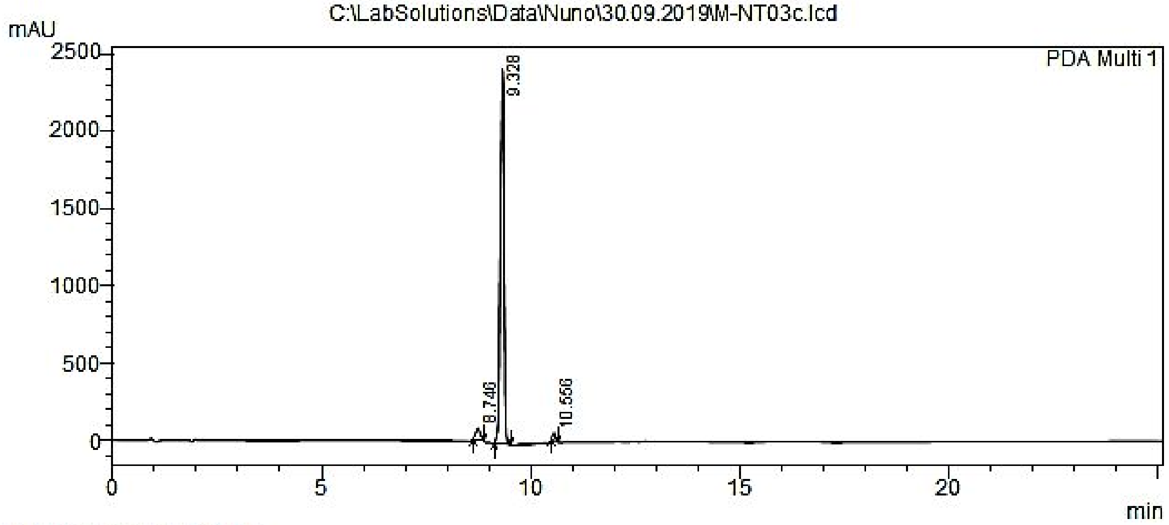

1 PDA Multi 1/210nm4nm

PeakTable

PDA Ch1 210nm 4nm

Peak#	Name	Ret. Time	Area	Area %
1	RT8.746	8.746	510528	3.199
2	RT9.328	9.328	15194130	95.197
3	RT10.556	10.556	256066	1.604
Total			15960725	100.000

Purity

Peak# : 2
Retention Time : 9.328
Compound Name : RT9.328

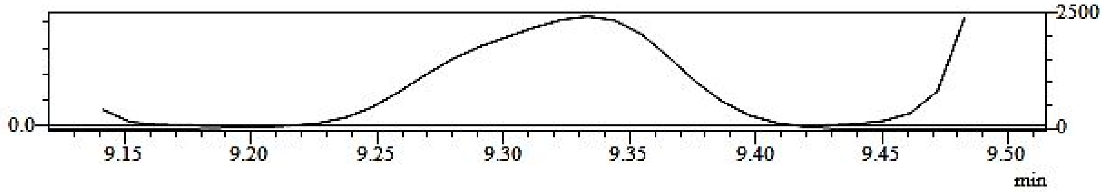

Impurity	:Not Detected
Peak purity index	: 0.999997
Single point threshold	: 0.999978
Minimum peak purity index	: 18

Figure S43 - HPLC spectra of the final compound 6a.

==== Shimadzu LCsolution Analysis Report ====

C:\LabSolutions\Data\Nuno\p-NT04c.lcd

Acquired by	: Admin
Sample Name	: p-NT04c
Sample ID	: p-NT04c
Vail #	: 15
Injection Volume	: 5 uL
Data File Name	: p-NT04c.lcd
Method File Name	: ACN+0,1TFA-H2O+0,1TFA.lcm
Batch File Name	: BatchThais03-12-17.lcb
Report File Name	: Report model LAPESSB.lcr
Data Acquired	: 10/9/2019 15:21:45
Data Processed	: 1/10/2019 21:50:12

<Chromatogram>

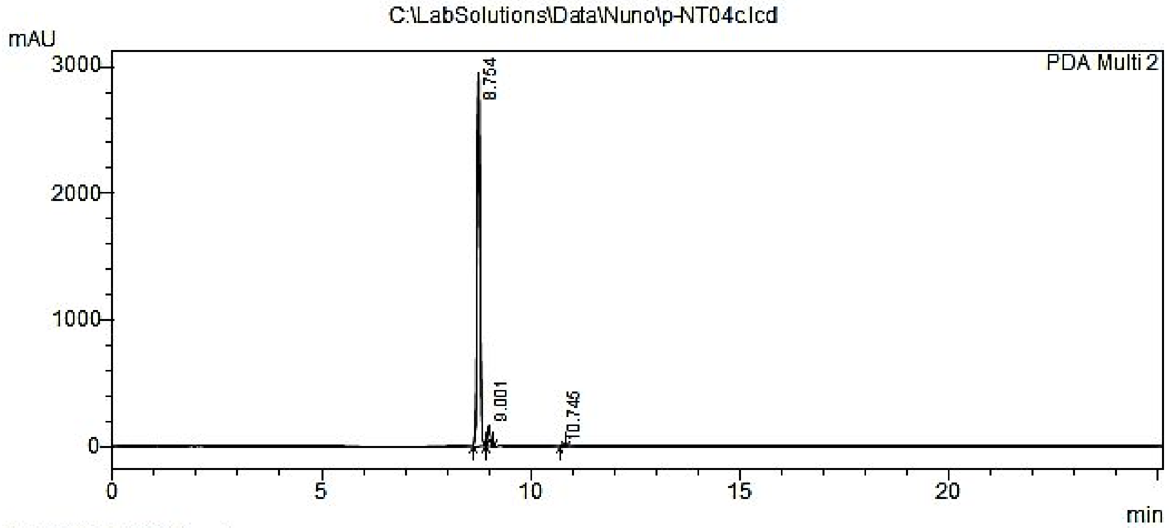

1 PDA Multi 2/261nm4nm

PeakTable

PDA Ch2 261nm 4nm

Peak#	Name	Ret. Time	Area	Area %
1	RT8.754	8.754	14420022	95.307
2	RT9.001	9.001	667822	4.414
3	RT10.745	10.745	42167	0.279
Total			15130011	100.000

Purity

Peak# : 1
Retention Time : 8.754
Compound Name : RT8.754

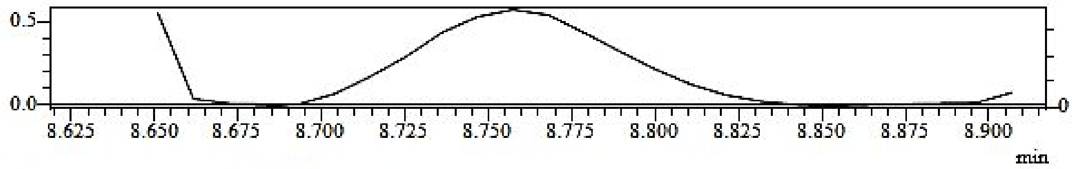

Impurity :Detected at 8.77 min
Peak purity index : 0.999732
Single point threshold : 0.999992
Minimum peak purity index : -260

Figure S44 - HPLC spectra of the final compound 6b.

==== Shimadzu LCsolution Analysis Report ====

C:\LabSolutions\Data\Nuno\3.10.2019\M-NT04c.lcd

Acquired by	: Admin
Sample Name	: M-NT04c
Sample ID	:
Vail #	: 11
Injection Volume	: 5 uL
Data File Name	: M-NT04c.lcd
Method File Name	: ACN+0,1TFA-H2O+0,1TFA.lcm
Batch File Name	: Batch_3.10.2019.lcb
Report File Name	: Default.lcr
Data Acquired	: 3/10/2019 12:54:33
Data Processed	: 3/10/2019 16:12:05

<Chromatogram>

C:\LabSolutions\Data\Nuno\3.10.2019\M-NT04c.lcd

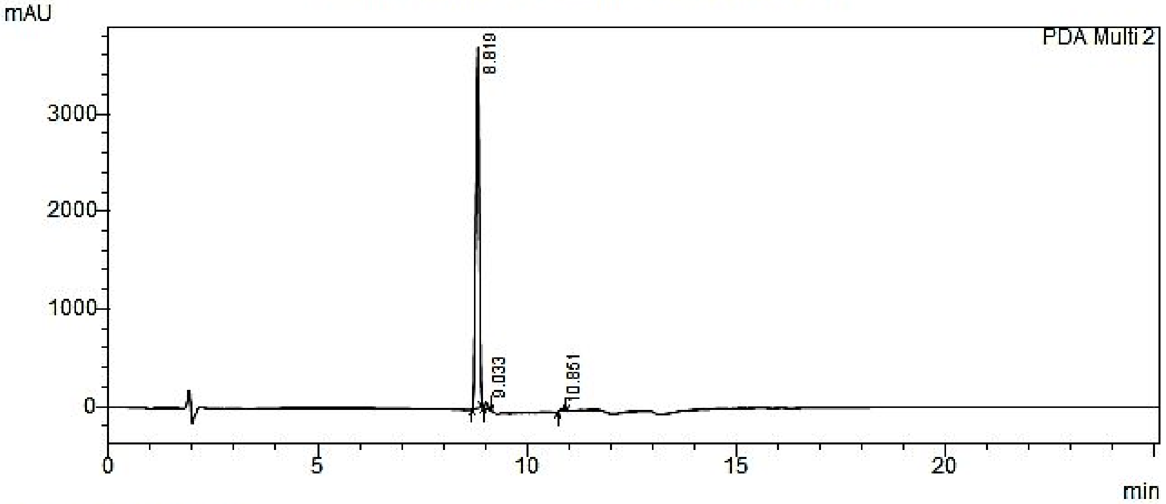

1 PDA Multi 2/214nm4nm

PeakTable

PDA Ch1 239nm 4nm

Peak#	Name	Ret. Time	Area	Area %
1		8.818	11907830	96.621
2		9.034	303903	2.466
3		10.853	112470	0.913
Total			12324204	100.000

Purity

Peak# : 1
Retention Time : 8.819
Compound Name : RT8.819

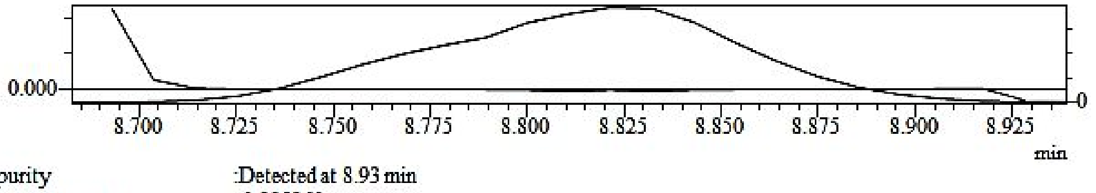

Impurity :Detected at 8.93 min
Peak purity index :0.995360
Single point threshold :0.996858
Minimum peak purity index : -1497

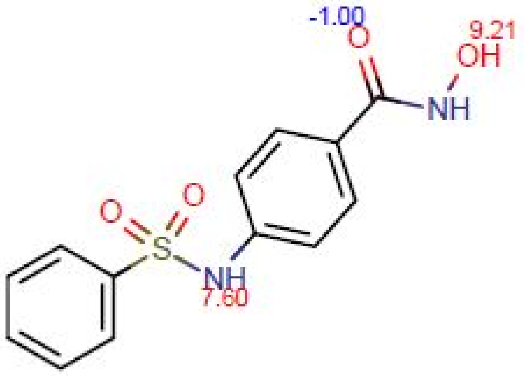

Strongest acidic pKa: 7.6

Strongest basic pKa: -1

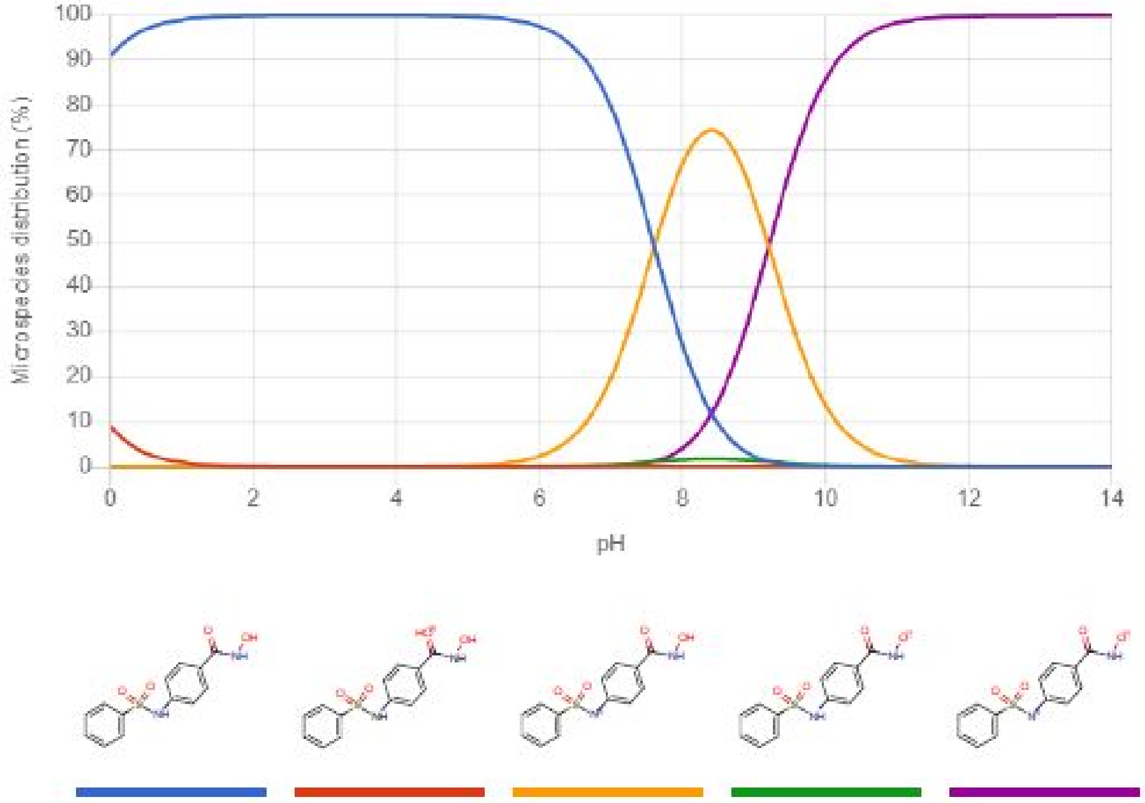

Figure S45 - pKa curves for compounds 3a. Calculated with Chemicalize software.

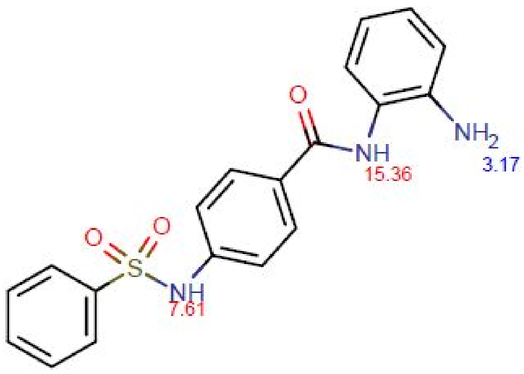

Strongest acidic pKa: 7.61

Strongest basic pKa: 3.17

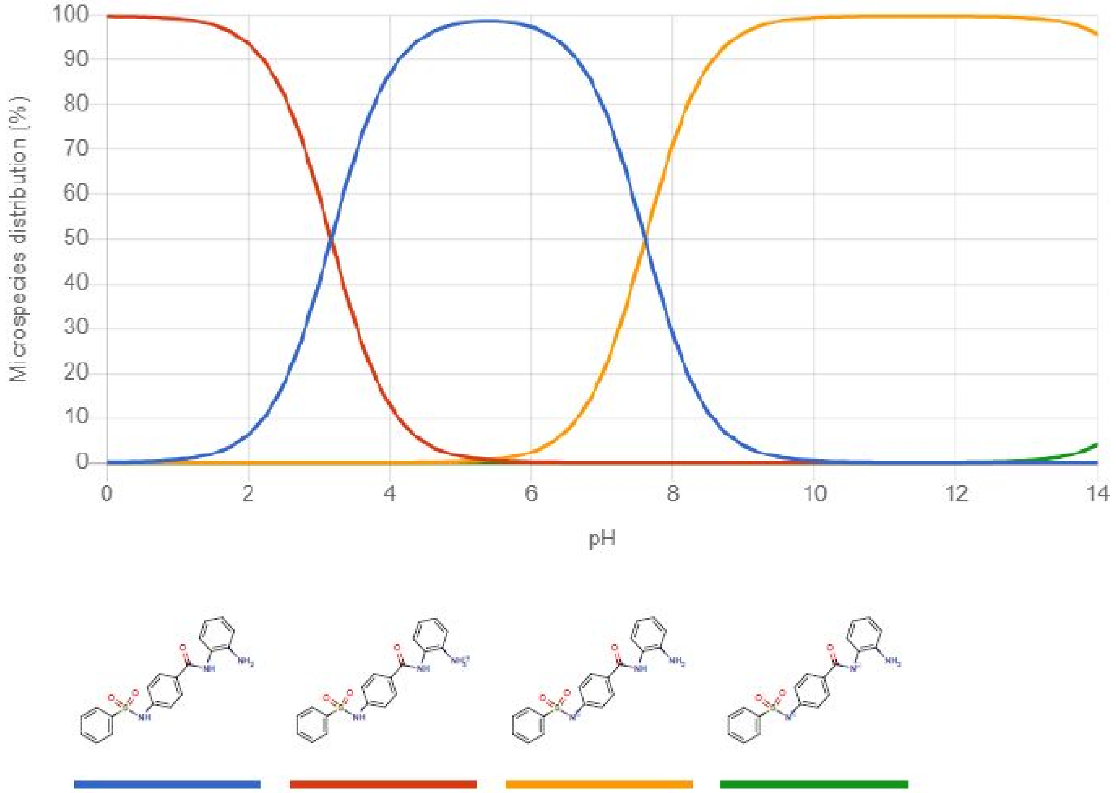

Figure S46 - pKa curves for compounds 6a. Calculated with Chemicalize software.

Table S1 - Summary of the simulation's data. Protein ligand interaction frequency separated by chemical moiety (Zn^{2+} stabilization control, ZBG: Zinc binding group, linker and cap), calculated from the cumulative trajectory of 500 ns (5x100 ns). HD: hydrophobic contacts. Root mean square deviation for protein's backbone ($RMSD_{prot}$) and ligand's heavy atoms (RMS_{lig}). Predicted binding energy calculated using MM/GBSA (dG for raw and LN for ligand efficiency) and decomposed in relevant terms (Cou: Coulombic, Lipo: lipophilic terms).

	**Dentate**	BI	MON	BI		BI	MON	BI*	BI		BI	BI	BI		BI	MON		BI	MON		BI	BI*
**Enz.**	**HDAC1**	**TSA**	**3a**	**6a**	**HDAC2**	**TSA**	**BNZ**	**3a**	**6a**	**HDAC3**	**TSA**	**3a**	**6a**	**HDAC6**	**TSA**	**3a**	**HDAC8**	**TSA**	**3a**	**HDAC10**	**TSA**	**3a**
Zn 2+	**D264**	100	100	100	**D269**	100	100	100	100	**D259**	100	100	100	**H651**	100	100	**D267**	100	100	**D265**	100	100
	**H141**	100	68	100	**H145**	79	99	---	100	**H172**	100	100	100	**D649**	100	100	**H180**	100	100	**H174**	100	97
	**D176**	100	100	100	**D181**	100	100	100	100	**D170**	100	100	100	**D742**		100	**D178**	100	100	**D172**	100	94
	**H178**	100	100	100	**H183**	100	100	100	100					**E779**		99	**H142**	100		**H134**	100	84
ZBG	**H140**	16			**R39**	32	59			**H135**		34	35	**H610**	100		**H142**	98		**H134**	99	69
	**H141**			43	**G143**	59				**G143**	49		99	**G619**		46	**H143**	60		**H135**	32	
	**G149**	96	29		**H145**		99	76	79	**H172**			32	**Y782**	93	31	**G151**		27	**Y305**	HD	HD
	**D176**			95	**H146**		74	24	72	**H173**		23					**G305**	33				
	**Y303**	41	21		**G154**	24	86		98	**Y298**	32											
Link	**G149**			88	**F155**				40	**F144**		48	45	**F620**		32						
	**F150**		40	63	**Y308**	HD		21														
Cap	**D99**			26	**D104**				25	**D93**		48	27	**F620**	27	HD	**F152**	20		**D91**	21	
	**H178**			28	**L144**	HD				**F144**	HD									**F202**	HD	
	**F150**	48	30	37	**F155**	43			37	**F200**		25	26							**W203**	HD	
	**F205**		24	25	**H183**				25	**L266**		HD										
					**F210**			HD	31													
RMS	**Prot**	0.19	0.16	0.13	**Prot**	0.20	0.13	0.23	0.14	**Prot**	0.14	0.14	0.11	**Prot**	0.08	0.14	**Prot**	0.21	0.22	**Prot**	0.12	0.28
	**Lig**	0.57	1.00	0.86	**Lig**	0.58	0.20	0.98	0.43	**Lig**	0.35	0.87	0.75	**Lig**	0.38	0.96	**Lig**	1.13	2.21	**Lig**	0.79	0.97
Energy	**dG**	-14.8	-12.6	-11.1	**dG**	-16.9	-18.8	-15.1	-21.3	**dG**	-8.4	6.7	-13.2	**dG**	-29.8	-17.8	**dG**	-7.6	-4.4	**dG**	-10.8	-2.9

	**Cou**	-38.3	-19.4	-20.1	**Cou**	-34.1	-41.2	-24.5	-33.2	**Cou**	-49.0	-24.3	-29.8	**Cou**	-97.9	-18.9	**Cou**	-60.3	-24.7	**Cou**	-42.0	-23.1
	**Hbond**	-1.0	-0.7	-1.7	**Hbond**	-0.9	-2.1	-1.1	-2.4	**Hbond**	-1.0	-0.6	-1.7	**Hbond**	-2.0	-1.1	**Hbond**	-1.5	-0.8	**Hbond**	-1.9	-0.9
	**Lipo**	-13.5	-12.6	-21.2	**Lipo**	-13.5	-23.5	-13.7	-22.8	**Lipo**	-15.5	-13.1	-21.7	**Lipo**	-17.2	-12.8	**Lipo**	-11.5	-12.0	**Lipo**	-17.0	-9.8
	**LN**	-3.6	-3.2	-2.6	**LN**	-4.1	-4.6	-3.8	-5.0	**LN**	-2.1	1.7	-3.1	**LN**	-7.3	-4.5	**LN**	-1.9	-1.1	**LN**	-2.6	-0.7

Table S2 - HDAC's chosen structures or models employed in this study and their truncated sequence positions.

Gene	PDB/Accession	Residues	Ligands (dentate)
HDAC1	5ICN	R8-A376	TSA (di)
HDAC2	3MAX	A12-L378	TSA (di), BNZ
HDAC3	4A69	A2-N370	TSA (di)
HDAC6	5EDU	A477-R835	TSA (di)
HDAC8	1T64	L14-V377	TSA (di)
HDAC10	AF-Q969S8-F1	M1-Q360	TSA (di)
